# Osteological and Soft-Tissue Evidence for Pneumatization in the Cervical Column of the Ostrich (*Struthio camelus*) and Observations on the Vertebral Columns of Non-Volant, Semi-Volant and Semi-Aquatic Birds

**DOI:** 10.1371/journal.pone.0143834

**Published:** 2015-12-09

**Authors:** Naomi E. Apostolaki, Emily J. Rayfield, Paul M. Barrett

**Affiliations:** 1 Department of Earth Sciences, University of Bristol, Bristol, United Kingdom; 2 Department of Earth Sciences, Division of Vertebrates, Anthropology and Palaeobiology, Natural History Museum, London, United Kingdom; Raymond M. Alf Museum of Paleontology, UNITED STATES

## Abstract

Postcranial skeletal pneumaticity (PSP) is a condition most notably found in birds, but that is also present in other saurischian dinosaurs and pterosaurs. In birds, skeletal pneumatization occurs where bones are penetrated by pneumatic diverticula, membranous extensions that originate from air sacs that serve in the ventilation of the lung. Key questions that remain to be addressed include further characterizing (1) the skeletal features that can be used to infer the presence/absence and extent of PSP in birds and non-avian dinosaurs, and (2) the association between vertebral laminae and specific components of the avian respiratory system. Previous work has used vertebral features such as pneumatic foramina, fossae, and laminae to identify/infer the presence of air sacs and diverticula, and to discuss the range of possible functions of such features. Here, we tabulate pneumatic features in the vertebral column of 11 avian taxa, including the flightless ratites and selected members of semi-volant and semi-aquatic Neornithes. We investigate the associations of these osteological features with each other and, in the case of *Struthio camelus*, with the specific presence of pneumatic diverticula. We find that the mere presence of vertebral laminae does not indicate the presence of skeletal pneumaticity, since laminae are not always associated with pneumatic foramina or fossae. Nevertheless, laminae are more strongly developed when adjacent to foramina or fossae. In addition, membranous air sac extensions and adjacent musculature share the same attachment points on the vertebrae, rendering the use of such features for reconstructing respiratory soft tissue features ambiguous. Finally, pneumatic diverticula attach to the margins of laminae, foramina, and/or fossae prior to their intraosseous course. Similarities in PSP distribution among the examined taxa are concordant with their phylogenetic interrelationships. The possible functions of PSP are discussed in brief, based upon variation in the extent of PSP between taxa with differing ecologies.

## Introduction

### The avian respiratory system

The avian respiratory system is composed of two immobile, dorsally fixed lungs, which are the sites of gas exchange, and (variably) nine large air sacs extending from the lungs via diverticula, which act as mechanical bellows to ventilate the lungs but that have very limited potential (5%) for gas exchange [1–4]. Lung volume remains constant during breathing, whereas the air sacs contract and expand during ventilation and occupy a substantial proportion of the body cavity [3,5,6]. In most birds, the lung is functionally and anatomically subdivided into paleopulmo and neopulmo portions. Paleopulmonic airflow is unidirectional during the course of the respiratory cycle [3,5,7]. In contrast, neopulmonic airflow is bidirectional, going toward the caudal air sacs and away from them during inspiration and expiration, respectively [7]. Most ratites have poorly developed neopulmonic [8] but well-developed palaeopulmonic parabronchi, although emus have minimally developed neopulmo portions in their lungs [5,7]. All penguins have strictly paleopulmonic lungs; i.e., they have no neopulmonic parabronchi [2–4,7]. Ducks, grebes, and loons have both neopulmonic and paleopulmonic lungs [3,7].

The air sacs are very thin and membranous in composition, and are connected to the primary as well as the secondary bronchi by means of ostia. The air sacs consist of most of the respiratory system’s volume [5,6,8,9]. In most birds, the nine air sacs are mainly categorized into cranial (anterior) and caudal (posterior) groups [3,8,10–14]. According to Powell [3], the paired air sacs along the cervical vertebrae, the unpaired air sac of the clavicular area, and the paired air sacs of the cranial thoracic area compose the cranial set while the paired air sacs of the caudal thoracic area and the paired air sacs of the abdominal area compose the caudal set [3]. The ostrich (*Struthio camelus*) has 10 air sacs due to the acquisition of a paired clavicular air sac [15].

The origin of the avian respiratory system has been discussed in many publications [16–19]. Unidirectional airflow was regarded as unique to crown Aves, but recent studies [20–22] have demonstrated its occurrence in crocodilians and squamates. Specifically, artificially ventilated lungs of *Alligator mississippiensis* exhibit a unidirectional flow of inserted fluid “through the lungs…in a strikingly bird-like pattern” ([20]:339). More elaborately, Farmer and Sanders [20] report unidirectional flow in alligator lungs that are both artificially ventilated, as well as in live animals breathing naturally with surgically implanted thermistor flow probes in individual pulmonary bronchi. Similarly, measurements of the airflow in *Crocodylus niloticus* lungs [23] show considerable similarities to those of alligators [20] and birds [8]. Schachner et al. [21] demonstrated the existence of unidirectional airflow in a specific region of the lungs/respiratory system of the savannah monitor lizard (*Varanus exanthematicus*) and Cieri et al. [22] showed that the lungs of the green iguana (*Iguana iguana*), also facilitate unidirectional flow of ventilation suggesting that “unidirectional flow is not an adaptation for expanding aerobic capacity and did not arise coincident with vigorous sustained locomotion or with endothermy” ([22]:17220). Such discoveries allow us to place the origin of unidirectional lung ventilation near to the base of Diapsida, prior to the split into Archosauromorpha and Lepidosauromorpha [22], although it is also plausible that the ability was acquired independently in the two groups. Experimental work on the domestic fowl by Brakenbury and Amaku [24,25] showed that flow patterns of ventilation do not change even when the ostia leading to the majority of the air sacs are occluded (including the abdominal sac), underscoring the importance of unidirectional airflow [24,25].

### Identification of postcranial skeletal pneumaticity in extant Aves

In extant birds, the air sac diverticula invade the postcranial skeleton during ontogeny as they expand, resulting in remodelling of both cortical and cancellous bone [4] but without damaging the bone structurally or biomechanically [26]. This postcranial skeletal pneumaticity (PSP) is expressed as the consistent pneumatization of certain groups of postcranial bones by specific diverticula [4, 6,13,27,28]. Cervical air sacs tend to pneumatize both cervical and anterior thoracic vertebrae and their associated ribs [4,6,28,29]. Thoracic vertebrae may also be pneumatized directly from lung diverticula originating from the parabronchi [2,30]. The clavicular air sac pneumatizes the humerus, sternum, sternal ribs and pectoral girdle, whereas the abdominal air sacs pneumatize the posterior thoracic vertebrae, the synsacral vertebrae, the caudal vertebrae, the pelvis, and the hind limbs (e.g., [3,4,30,31]). By contrast, the cranial thoracic air sacs do not tend to pneumatize any portion of the postcranial skeleton (e.g., [3,6]) in the species studied thus far, except a few species of budgerigar [27]. During embryonic development, pneumatic diverticula attach to the bone, inducing resorption and then penetrating the cortical bone [4,32,33], either along existing neurovascular foramina or by creating new foramina. By definition, all of these openings become pneumatic foramina once the diverticula have invaded the bone [4,28,34,35]. The diverticula then displace the bone marrow as they extend through the medullary cavity, replacing it with epithelium-lined outgrowths of the air sacs [4,33]. The invasion of some bones by diverticula during the pneumatization process is well documented, but less is known about the developmental mechanisms behind pneumatization [36].

After studying the vertebral columns of extant crocodiles, birds, and saurischian dinosaurs, O'Connor [4] and Wedel [37] proposed several criteria for distinguishing pneumatic perforations (foramina) and excavations (fossae) from non-pneumatic (i.e., vascular or neural) openings. These authors noted that a pneumatic foramen (i.e., that which encompasses a diverticulum) has a distinct, smooth and rounded margin, which is usually oval in outline, with a clearly visible hole penetrating the bone [4,37]. In birds, pneumatic foramina on bony tissues are usually much larger (at least twice the size) than purely vascular or neural foramina, with the latter measuring no more than 1 mm in diameter, having a more circular and flat rim in contrast to the more prominent lip that frames a pneumatic foramen (pers. obs.) (e.g., [4,18,28,29,32,37]). Wedel (pers. comm.) has pointed out that in very small birds, such as hummingbirds, the pneumatic foramina would be so small as to be indistinguishable from neurovascular foramina. At the other extreme, whales have vascular foramina in their vertebrae up to 3 mm in diameter (Wedel, pers. comm.; also see [38]). Thus, when studying extinct taxa, size alone cannot be used to distinguish pneumatic and neurovascular foramina: consequently, foramen wall texture, shape, and position need to be taken into account [4,28,38]. Nevertheless, there are cases where an opening may house both pneumatic and other soft tissues (e.g., adipose tissues) [28] and this observation has been verified by observations on the vertebral fossae of birds and crocodyliforms (e.g., [17,28,38,39]).

Pneumatic foramina can also be distinguished by their location and number. Non-pneumatic foramina are usually located on the ventrolateral surface of the centrum and, sometimes, on the inner wall of the neural canal, and are much less frequent in number (usually no more than two) than pneumatic foramina [4,29]. Furthermore, pneumatic foramina can form on every surface of a vertebra, particularly on the centrum, transverse process, neural arch, and neural spine (pers. obs.; also see [4,28,36–39]).

The identification of pneumatic fossae is more ambiguous because they can house a variety of tissues. For example, as mentioned earlier, the shallow excavations on extant crocodile vertebrae lack pneumatic features and contain adipose tissue, as well as acting as insertion and origination areas for musculature [28]. Similar comments apply to the excavations identified as 'fossae' in birds, although pneumatic structures also fill these depressions [28]. Nevertheless, pneumatic fossae in birds tend to be rather deep and wide, usually with laminated margins, and are smooth-edged [28,29]. They often have small foramina within their boundaries, or may lead to a cluster of smaller interconnected fossae that further penetrate the bone.

Pneumatic bones have several different characteristics from apneumatic bones [26,28,29,34,39]. A pneumatic bone is lighter as its marrow has been replaced by epithelial outgrowths of the invasive pneumatic diverticula (e.g., [6,26,36]). Such bone is also less oily in texture and lighter in color [29]. Pneumatic bones have less vascularization and possess features such as pneumatic foramina and pneumatic fossae [29]. In some cases, the pneumatic diverticulum does not completely invade the bone, but instead leaves an excavation or depression (a blind-ending fossa) on the surface of the bone [33]. Bone pneumatization is reduced in the postcranial skeleton of all diving birds such as gulls, ducks, cormorants, loons, grebes, rails, and penguins [6,36].

Generalizations regarding the variability and level of development of avian PSP, such as reduced pneumaticity in diving forms, are numerous (e.g., [5,6,36,40]) but, only recently, this subject has undergone phylogenetic scrutiny in detail. In his quantitative and comparative studies of avian pneumaticity O’Connor [4,28,36] has documented a common pattern in anseriforms (ducks, geese, swans) in which the cervical and thoracic vertebrae were pneumatized by the lungs as well as by the diverticula of the cervical and abdominal air sacs. O’Connor identified this as the ‘common anseriform pattern’ [4,28,36], with several deviations from it, interestingly noting reductions and expansions of the expression of pneumaticity in different avian clades. For example, ducks that utilize diving as a method of foraging showed reduced, or even completely absent skeletal pneumatization [36]. The correlation between pneumaticity and body size was also investigated, resulting into a positive relationship between the Pneumaticity Index (PI%—a metric that was developed for quantifying skeletal pneumaticity) and body mass ([4,36]; see also [41]). The PI% (Table 1) can be calculated by dividing the number of pneumatized anatomical units (for example, vertebrae) belonging to a particular set (vertebral column) over the total number of anatomical units of that set [4]. Use of the PI% allows quantification of the extent of pneumaticity and comparison of the degree of relative pneumaticity among species with different vertebral counts [4]. It was also observed that, as body mass increased, the extend of postcranial skeletal pneumatization was also increased [36].

**Table 1:** General status of each avian taxon studied. Tabulation of bird taxa with their orders, indicating locality, state of preservational condition of the specimen (St.pr.con.), number of specimens, pneumaticity status, Pneumaticity Index (PI%), ontogenetic stage, and registered specimen number (Reg.sp.no.). Abbreviations: D, diver; FL, flyer; FLS, flightless; ND, non-diver; P, pneumatic; SP, semi-pneumatic.

Taxa/common name	Locality	St.pr.con.	No.spec.	Life mode/Pneumaticity status	PI%	Ontogenetic stage	Reg.spec.no.
**Tinamiformes Tinamou**	Brazil, Sierra Chapada, Argentina	Complete, loose	5	FL/ND/P	69/77/92/92/92	Adults	S/1972.1.23 S/1972.2.6.7 S/1972.1496 S/1972.3.24.5 S/1972.2.16.62
**Apterygiformes Kiwi**	New Zealand	Complete, articulated	3	FLS/ND/P	23/61/77	Adult Adult Subadult	1456 1488 1458
**Dinornithiformes Moa**	New Zealand	Complete, articulated	1	FLS/ND/P	70	Subadult	Cg976
**Casuariiformes Cassowary**	N/A	Complete articulated, skull missing	1	FLS / ND / P	54	Adult	Af963
**Dromaiformes Emu**	N/A	Complete, articulated	1	FLS/ND/P	77	Subadult	Ab4163
**Struthioniformes Ostrich**	N/A Devon, UK	Complete, articulated Neck only	2	FLS/ND/P	85	Adult Subadult	Af962 Ag1174
**Rheiformes Rhea**	South America	Complete, loose	1	FLS/ND/P	100	Adult	2.5.1
**Anseriformes Duck**	N/A	Complete, articulated	2	FL/SP	77/77	Subadults	N/A
**Gaviiformes Loon**	Sennen Cove, Cornwall, UK	Complete, loose	2	FLS/D/SP	46/46	Adults	S/1996.68.1 S/1985.18.1
**Podicipediformes Grebe**	London, UK	Complete, loose	1	D/SP	61	Adult	S/1952.1.47
**Sphenisciformes Penguin**	N/A	Complete, articulated	2	FLS/D/SP	23/69	Adults	N/A

During ontogeny, the first to be pneumatized are the cervical and anterior thoracic vertebrae, by the cervical air sac diverticula [15,35]. The diverticula extending from the abdominal air sacs pneumatize the posterior thoracic vertebrae and, later in ontogeny, the synsacrum [36]. Despite the presence of ‘intermediate’ states of pneumaticity among birds, two main cases of PSP have been recorded in the small subset of bird clades that has been examined in detail: birds whose postcranial skeleton possesses high expression of pneumatic foramina and deep fossae (pneumatic birds: e.g., Struthioniformes [ostriches], Rheiformes [rheas], Casuariiformes [cassowaries]) (e.g., [3,8,15]) and birds whose skeleton has limited expression of pneumatic foramina and frequent expression of blind-ending fossae, resulting in reduced or absent PSP (e.g., [4,8,11,36]) (semipneumatic and apneumatic birds: e.g., Apterygiformes [kiwis], Sphenisciformes [penguins], Anseriformes [ducks], Gaviiformes [loons], Podicipediformes [grebes]). All ratites exhibit highly expressed PSP (this study) in most vertebral and appendicular elements (see Tables 2–5 and S1 Table and S3–S9 Tables) and, therefore, high PI%s, in which the humeri and femora are pneumatized (via the interclavicular and abdominal air sacs, respectively [2,4–6]). In addition, there are few rare cases (such as in rhea and penguin–see S1 Table, S7 Table and S10 Table) where the distal leg elements (tibiotarsus and tarsometatarsus) exhibit foramina, which are included in our PI% measurements but they are considered ambiguous with respect to pneumatization until dissection studies can confirm that they are indeed invaded by air sac diverticula. PSP in other avian clades (e.g., passerines, raptors) has not been extensively studied. The strong correlation between PSP and the presence of air sac diverticula in extant birds can be used to infer the presence of these diverticula in the absence of preserved soft tissues [4,18,28,30]. Therefore, PSP can be used as a possible indicator of these soft tissue structures in extinct taxa and thus provide information on the evolution of amniote respiratory systems.

**Table 2:** Distribution of pneumatic elements on the cervical column per taxon. Presence (+) and absence (-) of pneumatic characters found on the cervical vertebrae from specimens of the 11 taxa studied. The penguin with the pneumatised vertebrae is *Pygoscelis papua* (NHMUK unregistered). For more details see S1 Table and S3–S13 Tables. Abbreviations: PF, pneumatic foramina; F, fossae; L, laminae; Sept., septated.

Cervical Vertebrae								
Taxon/common name	No. of vertebrae	Pneumatic foramina	Laminae	Fossae	PF+L	F+L	PF+F	Sept.PF
**Tinamiformes Tinamou**	**10–15**	**+**	**+**	**+**	**-**	**-**	**+**	**-**
**Apterygiformes Kiwi**	**14**	**+**	**+**	**+**	**-**	**+**	**-**	**-**
**Dinornithiformes Moa**	**15**	**+**	**+**	**+**	**+**	**+**	**-**	**-**
**Struthioniformes Ostrich**	**15–17**	**+**	**+**	**+**	**+**	**+**	**+**	**+**
**Rheiformes Rhea**	**14**	**+**	**+**	**+**	**+**	**+**	**+**	**+**
**Casuariiformes Cassowary**	**14**	**+**	**+**	**+**	**+**	**+**	**+**	**+**
**Dromaiformes Emu**	**18**	**+**	**+**	**+**	**+**	**+**	**+**	**+**
**Anseriformes Duck**	**14**	**+**	**+**	**+**	**+**	**+**	**+**	**-**
**Gaviiformes Loon**	**15**	**-**	**+**	**+**	**-**	**-**	**-**	**-**
**Podicipediformes Grebe**	**19**	**+**	**+**	**+**	**-**	**-**	**-**	**-**
**Sphenisciformes Penguin**	**13–14**	**+**	**+**	**+**	**-**	**-**	**-**	**+**

**Table 3:** Distribution of pneumatic elements of thoracic column per taxon. Presence (+) and absence (-) of pneumatic characters found on the thoracic vertebrae from specimens of the 11 taxa studied. The penguin with the pneumatised vertebrae is *Pygoscelis papua* (NHMUK unregistered). For more details see S1 Table and S3–S13 Tables. Abbreviations: PF, pneumatic foramina; F, fossae; L, laminae; Sept., septated.

Thoracic Vertebrae								
Taxa/common name	No. of vertebrae	Pneumatic foramina	Laminae	Fossae	FP+L	F+L	FP+F	Sept.PF
**Tinamiformes Tinamou**	**6–7**	**+**	**+**	**+**	**+**	**-**	**-**	**+**
**Apterygiformes Kiwi**	**8**	**+**	**-**	**-**	**-**	**-**	**-**	**-**
**Dinornithiformes Moa**	**7**	**+**	**+**	**+**	**+**	**+**	**-**	**+**
**Struthioniformes Ostrich**	**6**	**+**	**+**	**+**	**+**	**+**	**+**	**+**
**Rheiformes Rhea**	**8**	**+**	**+**	**+**	**+**	**-**	**+**	**-**
**Casuariiformes Cassowary**	**7**	**+**	**+**	**+**	**+**	**+**	**+**	**+**
**Dromaiformes Emu**	**7**	**+**	**+**	**+**	**+**	**+**	**+**	**+**
**Anseriformes Duck**	**6–8**	**+**	**+**	**+**	**-**	**-**	**+**	**+**
**Gaviiformes Loon**	**7**	**-**	**+**	**+**	**-**	**-**	**-**	**-**
**Podicipediformes Grebe**	**8**	**+**	**+**	**+**	**-**	**-**	**-**	**+**
**Sphenisciformes Penguin**	**7–9**	**+**	**-**	**+**	**-**	**-**	**-**	**+**

**Table 4:** Distribution of pneumatic elements of synsacral column per taxon. Presence (+) and absence (-) of pneumatic characters found on the synsacral vertebrae from specimens of the 11 taxa studied. The penguin with the pneumatised vertebrae is *Pygoscelis papua* (NHMUK unregistered). For more details see S1 Table and S3–S13 Tables. Abbreviations: PF, pneumatic foramina; F, fossae; L, laminae; Sept., septated.

Synsacral Vertebrae								
Taxon/common name	No. of vertebrae	Pneumatic foramina	Laminae	Fossae	PF+L	F+L	PF+F	Sept.PF
**Tinamiformes Tinamou**	**5–6**	**+**	**-**	**-**	**-**	**-**	**-**	**-**
**Apterygiformes Kiwi**	**7**	**+**	**-**	**-**	**-**	**-**	**-**	**-**
**DinornithiformesMoa**	**13**	**+**	**+**	**-**	**-**	**-**	**-**	**-**
**Struthioniformes Ostrich**	**15**	**+**	**+**	**-**	**+**	**-**	**-**	**+**
**Rheiformes Rhea**	**10**	**-**	**+**	**-**	**+**	**+**	**+**	**+**
**Casuariiformes Cassowary**	**17**	**-**	**-**	**+**	**+**	**+**	**+**	**-**
**Dromaiformes Emu**	**5**	**+**	**-**	**-**	**-**	**-**	**-**	**-**
**Anseriformes Duck**	**11–13**	**+**	**-**	**-**	**-**	**-**	**-**	**-**
**Gaviiformes Loon**	**10**	**+**	**-**	**-**	**-**	**-**	**-**	**-**
**Podicipediformes Grebe**	**15**	**+**	**-**	**-**	**-**	**-**	**-**	**-**
**Sphenisciformes Penguin**	**9–13**	**+**	**-**	**-**	**-**	**-**	**-**	**-**

**Table 5:** Distribution of pneumatic elements of caudal column per taxon. Presence (+) and absence (-) of pneumatic characters found on the caudal vertebrae from specimens of the 11 taxa studied. The penguin with the pneumatised vertebrae is *Pygoscelis papua* (NHMUK unregistered). For more details see S1 Table and S3–S13 Tables. Abbreviations: PF, pneumatic foramina; F, fossae; L, laminae; Sept., septated; N/A, non-available.

Caudal Vertebrae								
Taxa/common name	No. of vertebrae	Pneumatic foramina	Laminae	Fossae	PF+L	F+L	PF+F	Sept.PF
**Tinamiformes Tinamou**	**5**	**-**	**-**	**-**	**-**	**-**	**-**	**-**
**Apterygiformes Kiwi**	**6**	**-**	**-**	**-**	**-**	**-**	**-**	**-**
**Dinornithiformes Moa**	**N/A**	**N/A**	**N/A**	**N/A**	**N/A**	**N/A**	**N/A**	**N/A**
**Struthioniformes Ostrich**	**7**	**+**	**-**	**-**	**-**	**-**	**-**	**-**
**Rheiformes Rhea**	**4–5**	**+**	**-**	**-**	**-**	**-**	**-**	**+**
**Casuariiformes Cassowary**	**8**	**-**	**-**	**-**	**-**	**-**	**-**	**-**
**Dromaiformes Emu**	**7**	**-**	**-**	**-**	**-**	**-**	**-**	**-**
**Anseriformes Duck**	**5–7**	**+**	**-**	**-**	**-**	**-**	**-**	**-**
**Gaviiformes Loon**	**-**	**-**	**-**	**-**	**-**	**-**	**-**	**-**
**Podicipediformes Grebe**	**-**	**-**	**-**	**-**	**-**	**-**	**-**	**-**
**Sphenisciformes Penguin**	**7–9**	**-**	**-**	**-**	**-**	**-**	**-**	**-**

### Identifying PSP in extinct non-avian dinosaurs and pterosaurs

Postcranial pneumatic features have been documented in extinct non-avian archosauriforms for more than a century [42,43]. The anatomical and positional similarities of these features to those of modern birds, and the assumption that they were formed by homologous developmental processes, implies that they can provide evidence for the presence of at least some avian-like respiratory soft tissues (diverticula) in a variety of extinct archosaur taxa [28]. The most conservative phylogenetic optimizations indicate that PSP arose on three occasions within Ornithodira (within Pterosauria, Theropoda and Sauropodomorpha), although it seems likely that there might have had a single origin in the common ancestor of ornithodirans with subsequent loss in ornithischians [44–46]. It has also been suggested that PSP may represent an ancestral archosaurian characteristic [16,17], although this suggestion requires further study and is difficult to test [45].

#### Ornithodira: Theropoda

Early theropods, like the Late Triassic taxa *Herrerasaurus* and *Staurikosaurus*, exhibit well-developed laminated fossae (fossae framed by laminae) on their dorsal vertebrae [45]. Their cervical and caudal vertebrae lack pneumaticity. It has been suggested that laminae may indicate the presence of pneumatic diverticula [39], but the presence of laminae and fossae alone has been considered ambiguous evidence for an avian-like respiratory system [38,45]. Most theropods (e.g., abelisaurids, tetanurans) possess fully developed and variable PSP, including complex features like laminated fossae, laminated foramina, and foramina within fossae [47,36]. It has been demonstrated that throughout phylogeny, gradual expression of vertebral pneumatization begins in the dorsal series and expands to the cervical and sacrocaudal series [41]. Other non-PSP-related evidence from non-avian dinosaurs suggests they possessed avian-like ventilation [48,49]. The uncinate processes, specialized gastralia, sterna, and pelvic girdles in non-avian theropods provided attachment points for the same muscles that facilitate avian-style breathing in extant birds [48,49] and the gastralia, in particular, may have assisted theropods in aspiration [50]. Cursorial avian species, like ostriches and emus, do not possess highly developed pectoral muscles that are important for flying, and, therefore, the lever-arm action provided by the uncinate processes is of no use [48]. Non-avian maniraptoran dinosaurs possessed long uncinate processes [48], signifying an enhanced musculoskeletal mechanical advantage. As studies have demonstrated in extant avian species [48], such an advantage is functionally important in unidirectional ventilation. A caveat here is that alligators, crocodiles, varanids, and iguanas employ a unidirectional flow during ventilation [20–23] but lack uncinate processes.

#### Ornithodira: Sauropodomorpha

Postcranial (especially axial) pneumaticity is also present in sauropodomorphs [18,37–39,46]. These features are first seen in basal taxa (‘prosauropods’), where they are poorly expressed, but become more elaborate in eusauropods. The characters used to infer PSP vary from the presence of simple fossae on the external surfaces of the vertebral centra to internal honeycomb-like structures [18]. Many ‘prosauropods’ (e.g., *Plateosaurus*, *Thecodontosaurus*, *Eucnemesaurus*) show expression of laminated fossae (and rarely foramina) in their posterior cervical and anterior dorsal vertebrae, which become more complex in derived taxa close to the origin of sauropods [51]. Most of the fossae observed in prosauropods are blind-ending, so they do not provide strong evidence for invasive pneumatization [38]. Non-neosauropod eusauropods (e.g., *Tazoudasaurus*, *Antetonitrus)* possess pneumatic foramina and fossae in their vertebral columns and, especially, mamenchisaurids (e.g., *Mamenchisaurus*, *Omeisaurus*, *Shunosaurus*) exhibit complex pneumatization, potentially acquired independently of that seen in neosauropods ([18,52]). Neosauropods, i.e., Diplodocoidea and Macronaria, show increasing expression of complex pneumatic features (laminated pleurocoels, laminated fossae, foramina within shallow fossae) and a gradual remodelling of the vertebrae into complex lattice-like structures (camerate and camellate aeration) [18,37,38,46,53–56].

#### Ornithodira: Pterosauria

Pterosaur bones have been examined for almost as long as those of non-avian dinosaurs and recent work [44,57] has demonstrated the presence of PSP in the cervical and dorsal vertebrae and ribs of Late Triassic and Early Jurassic pterosaurs (e.g., *Raeticodactylus filisurensis*, *Eudimorphodon* sp., *Dimorphodon macronyx*) suggesting the possible presence of avian-like air sacs. Pneumatization of the axial column is also common in Jurassic and Cretaceous taxa (e.g., *Anhanguera santanae*; [44]).

### Aims of this study

Most research focusing on PSP has addressed the association between fossae, foramina, and invasive air sac diverticula by documenting their presence and variability in the vertebral columns of various avian, non-avian dinosaur, and pterosaur taxa. The inference of invasive air sacs is often considered the *sine qua non* for deducing the presence of avian-like respiratory features in extinct taxa. However, modern birds also possess diverticula that do not invade the postcranial skeleton, but intervene between soft tissues, such as muscles and organs. Moreover, this inference is not always secure, as Milani [58] and Perry [59] have shown that lizards, such as varanids and chameleons, have lung diverticula that do not invade the postcranial skeleton. Conversely, there are fish, such as *Pantodon*, which have diverticula extending from the swim bladder that invade the skeleton, most significantly the vertebral column [60,61], so the interactions between diverticula and osteology are sometimes unexpected. The presence/absence of these non-invasive diverticula is conjectural in fossil material due to the absence of osteological correlates [45,46,57]. Unfortunately, this deficit obscures the early evolution of avian-like respiratory systems, as it is likely that the first pneumatized archosaurs achieved the first steps toward full pneumatization by evolving air sacs that were not invasive (e.g., [36,45,62]) or, alternatively, that these first archosaurs had bony tissues invaded by lung diverticula instead of air sacs.

Vertebral laminae are often overlooked in studies of PSP, although it has been suggested that they are associated with diverticula [39,45]. Anatomical studies on extant birds (e.g., [4,6,8–10,12–14,16,28,36]) have not considered the detailed interrelationships between components of the air sac system and the laminae that consistently frame pneumatic fossae and foramina. We hypothesize that laminae may act as attachment sites for air sacs and diverticula close to their points of entry into bones, as well as supporting the non-invasive components of the air-sac system. Many extinct archosaurs (e.g., pseudosuchians, ornithischians) lack unambiguous evidence of pneumatic foramina, indicating that they lacked invasive air sac diverticula; nevertheless, these taxa often possess complex and well-developed vertebral lamination and deep vertebral fossae [45]. We chose *Struthio camelus*, the ostrich, as our primary taxon, mainly because of its size and the availability of materials, as well as its classification as a palaeognath, thus being less modified in light of flight adaptation than other avian clades (see also S1 File).

The aims of this study are: i) to determine the nature of the soft tissues that are associated with the vertebral laminae in *Struthio camelus*; ii) to investigate whether the presence of laminae and pneumatic structures (both soft tissue and hard tissue) are correlated; iii) to test whether the presence of laminae alone can be a good indicator of the presence of air sac diverticula.

In order to do this, we document the relationships between soft-tissue features (e.g., muscles, nerves, and pneumatic diverticula) and vertebral anatomy, primarily laminae and their associated fossae in the ostrich neck. We sought to determine if fossae and laminae can be used as osteological correlates of air sac diverticula in extant birds and, if so, what the implications of this relationship might be for understanding the structure and function of the non-avian dinosaur respiratory system. Finally, we assess whether there is a correlation between the occurrence of PSP in selected avian families and their phylogenetic relatedness.

### Institutional Abbreviations

University of Bristol, School of Earth Sciences (BRSUG), Bristol City Museum and Art Gallery (BRSMG), University of Bristol School of Veterinary Sciences (BRSUV), Natural History Museum, London and Tring (NHMUK).

## Materials and Methods

### Vertebral laminae

Vertebral laminae are osseous ridges connecting two or more vertebral landmarks and may also form the boundaries of vertebral fossae and foramina. They are present in many extant and extinct archosaurs [45,55,56]. Wilson and colleagues [55,56] documented 19–27 possible vertebral laminae in sauropod dinosaurs, many of which can also be found in other dinosaurs (including extant birds), crurotarsan archosaurs and pterosaurs, and developed a consistent terminology for these structures. Most archosaurs exhibit a common series of laminae that extend between the centrum and neural arch, and between various neural arch processes (e.g., centrodiapophyseal laminae, pre- and postzygodiapophyseal laminae), but sauropods and non-avian theropods possess many more that are not present in other archosaur groups (e.g., [18,28,36,38,41,44]). In sauropods, non-avian theropods, and occasionally birds, laminae of the same type may have anterior, middle, and posterior expressions, form lattice-like interconnections, or the vertebrae may even possess 'stranded', 'segmented', and 'accessory' laminae (e.g., [55,56]). Examples of these include the prespinal and postspinal laminae, which are positioned on the anterior and posterior surfaces of the neural spine, respectively. The standard abbreviations used below for these laminae and fossae are listed in S14 Table (following [55,56]).

### Osteological observations

The location and distribution of axial PSP was assessed in 11 avian families (Struthionidae, Rheidae, Casuariidae, Dromaiidae, Dinornithidae, Apterygidae, Tinamidae, Anatidae, Spheniscidae, Gaviidae, and Podicipedidae). These clades were chosen for their diverse locomotory modes (cursorial, semi-aerial, semi-aquatic).

Twenty-one specimens were examined with an emphasis on their cervical columns: two ostriches (*Struthio camelus*—one skeleton BRSMG Af 962 and one fresh neck specimen BRSMG Ag1174 series, used for dissection), one moa (*Emeus crassus—*BRSMG Cg 976), one emu (*Dromaius novaehollandiae*—BRSMG Ab 4163), one cassowary (*Casuarius galeatus—*BRSMG Af 963), one rhea (*Rhea americana—*NHMUK 2.5.1), three kiwis (*Apteryx australis haasti—*NHMUK 1456, *Apteryx australis lawri—*NHMUK 1488, *Apteryx oweni—*NHMUK 1458), five tinamous (*Crypturellus obsoletus—*NHMUK S/1972.1.23, *Crypturellus undulatus—*NHMUK S/1972.2.6.7, *Eudromia elegans—*NHMUK S/1972.1496, *Nothura maculosa—*NHMUK S/1972.3.24.5 and *Rhynchotus rufescens—*NHMUK S/1972.2.16.62), two unregistered penguins (*Pygoscelis papua*—NHMUK and *Pygoscelis antarcticus*—BRSUV), two ducks (*Anas* gen.—BRSUV- unregistered and *Melanitta* gen.—BRSMG Af 974), two loons (*Gavia adamsii—*NHMUK S/1996.68.1 and *Gavia stellata—*S/1985.18.1), and one grebe (*Podiceps major—*NHMUK S/1952.1.47).

The following information was recorded for each specimen (see Table 1): size measurements (total height and total length wherever applicable: see S2 Table), preservational state (e.g., complete, articulated, well-preserved, partially damaged); Pneumaticity Index (PI%) [4,28] and the specimen's resultant pneumaticity status (i.e., pneumatic, semi-pneumatic, apneumatic) based on the ratio of pneumatized bones (anatomical units [AUs]) in a given set over the total number of the bones of that set (vertebrae-vertebral column); ontogenetic stage (i.e., subadult, adult); presence/absence of pneumatic features relative to vertebral landmarks (position, orientation, size); and locomotor mode (i.e., non-volant, volant, diver, non-diver). Thirteen AUs (see Tables 2–5 and S1 Table) were designated for comparison between taxa with morphological differences in their vertebral columns, namely: cervical vertebrae (CV), dorsal (thoracic) vertebrae (DV/TV), synsacral vertebrae (SSV), caudal vertebrae (CAV), femur (FM), humerus (HM), scapula (SC), coracoid (CC), sternum (ST), ribs (RB, both dorsal and sternal ribs), furcula (FC), tibiotarsus (TT), and tarsometatarsus (TM). Pneumatic taxa are defined as those with PI>90%, semi-pneumatic with PI<90% and apneumatic with PI of 0% (Table 1 and S1 Table).

The distribution and presence of pneumaticity was also tabulated for all 11 taxa for each vertebral region (see Tables 2–5, S1 Table and S3–S13 Tables).

The presence of pneumaticity on either side of a bilaterally symmetrical AU (e.g., present on a left dorsal rib, but absent on the corresponding right dorsal rib) was considered sufficient to allow scoring as present in that AU.

For bones, muscles and osteological pneumatic features we follow the anatomical nomenclature of Baumel et al. [63] and Ghetie et al. [64].

### Dissection and observations on soft tissue and vertebral structures

The research described herein complied with protocols approved by the University of Bristol Ethics of Research Committee and adhered to the legal requirements of the country (UK) in which the research was conducted. ***No permits were required for the described study*, *which complied with all relevant regulations*.** No special permits or permit numbers were needed for the acquisition of the ostrich neck material. The ostrich neck was obtained directly from farmed ostriches that were culled for meat. The ostrich specimen came from MNS Ostriches Limited, an ostrich farm in Devon, UK. The ostriches are legally and humanely killed for meat production. The guidelines state that 'humanely' corresponds to be stunned electrically and then have their throats cut. This is performed by trained abattoir workers, and not by farmers or researchers. The heads and necks are by-products of the farming industry.

The dissection was carried out on a skinned 70 cm long neck of *Struthio camelus*, from a subadult farmed individual of about 6 months old that had been frozen since 2007. The ostrich neck was delivered to us decapitated and severed from the rest of the body, thus any anterior (i.e., cervicocephalic) or posterior (i.e., cervicothoracic) air sac diverticula were not present, resulting in incomplete and partially damaged air sacs and their diverticula on the anteriormost and posteriormost ends of the neck. Being in such a condition, the neck was not suitable for injecting with latex or any other appropriate material, prior to the dissection, in order to completely determine the exact morphology, position, and extent of the cervical air sacs and their diverticula along the neck. Nevertheless, the neck was not ruptured in any other places and the air sac diverticula were not damaged during the dissection. All types of tissues were found in place and unaltered, and their identifications were verified against relevant sources including Baumel [63] and Ghetie [64]. The available neck was composed of 15 cervical vertebrae (BRSMG Ag1174.1 –Ag1174.15). The atlas and axis were not present and the 17^th^ vertebra had been cut in half.

Predetermined groups of subcutaneous dermal and muscular tissues were systematically removed until the vertebrae were exposed without destroying the cervical air sac diverticula. The surface muscle groups were removed layer by layer, longitudinally across the neck. Once the vertebral connective muscles were reached, the air sac membranes were located and stretched with the aid of tweezers. Connective tissues were carefully removed to ensure that the air sac membranes would suffer the least damage possible.

After removal of the intervertebral tissues, the points at which the diverticula invaded the cervical vertebrae were recorded. Following this, all soft tissues were removed from the vertebrae and the positions of the pneumatic foramina, fossae, and laminae were recorded and compared with those obtained from other avian taxa.

Following tissue removal, the vertebrae were completely defleshed to facilitate measurements and photography. This was accomplished by bathing the vertebrae in simmering water at 200°C for three hours per day for two days, to macerate the residual soft tissue for further removal. The bones were then bleached to remove all remaining soft tissue traces, using 4 l of water with 800 ml household bleach and 100 ml of dishwashing surfactant. Fat residues were removed by immersion in cold water containing two spoonfuls of an enzymatic clothing detergent. The dissection and cleaning processes were recorded through notes, sketches, and digital photographs.

## Results

### Observations on the *Struthio camelus* cervical column

The ostrich neck is composed of 17 vertebrae, but the axis and atlas were missing from our specimen (BRSMG Ag1174.1–15), leaving 15 cervicals (CV3–17) in total. All measurements, unless otherwise stated, signify diameter.

**Fig 1:**
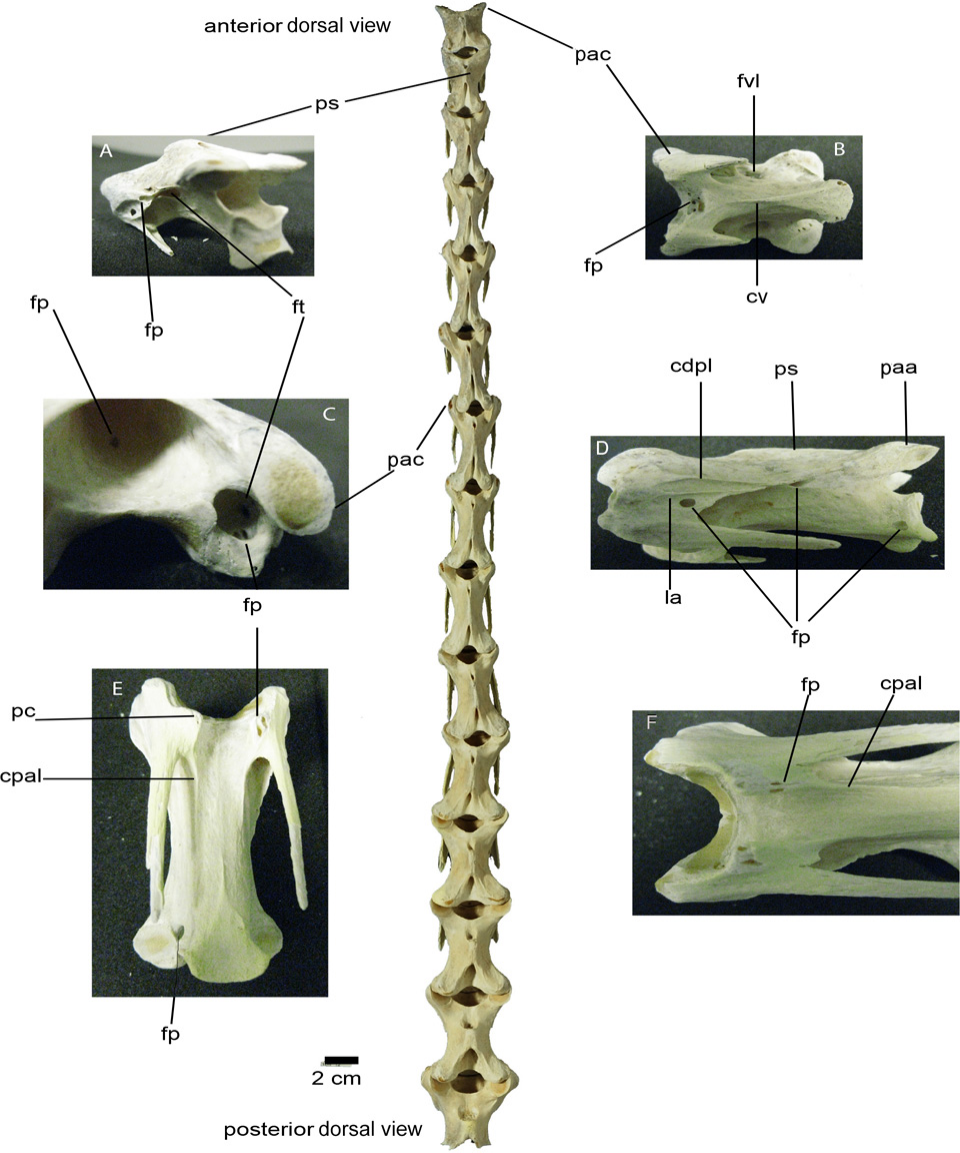
Dorsal view of ostrich (*Struthio camelus*) cervical column. **(A)** Posterolateral view of CV4 (BRS MG Ag1174.2), revealing the *foramen pneumaticum* ventral to the *lamina arcocostalis*, the *foramen pneumaticum* ventral to the processus transversus, and the *foramen transversarium*; note the vertebral foramen located posterior to the *processus spinosus*; **(B)** Ventral view of CV3 (BRSMG Ag1174.1) from which the *foramen vertebrale laterale*, the *crista ventralis* that runs along the *corpus*, and the pneumatic foramina on the anterior-most margin of the vertebral foramen can be viewed; **(C)** Left anteroventral view of a cervical vertebra (CV10: BRSMG Ag1174.8) exposing the pneumatic foramen on the inner wall of the vertebral foramen and the multiple pneumatic foramina within the costotransverse ring; **(D)** Left ventrolateral view of CV5 (BRSMG Ag1174.3) showing the centrodiapophyseal lamina, the *lamina arcocostalis* just above the pneumatic foramen on the arcocostal surface's posterior-most margin, and a pneumatic foramen on both the *corpus* along the line of the neurocentral suture and on the posterolateral surface of the cotyle; **(E)** Ventral view of CV11 (BRSMG Ag1174.9) exposing the centroparapophyseal laminae, two pneumatic foramina on the ventral side of the left *processus costarius* and one on the right side of the cotyle; **(F)** Ventral view of CV9 (BRSMG Ag1174.9) showing pneumatic foramina on both hypapophyses and both centroparapophyseal laminae. The scale bar corresponds to the main cervical column. The main cervical column photograph was taken by Simon Powell. Abbreviations: cv *crista ventralis*; cdpl centrodiapophyseal lamina; cpal centroparapophyseal lamina; pac *processus articularis cranialis*; paa *processus articularis caudalis*; fp *foramen pneumaticum*; ft *foramen transversarium*; fvl *foramen vertebrale laterale*; la *lamina arcocostalis*; pc *processus costarius*; ps *processus spinosus*.

CV3 (BRSMG Ag1174.1; length: 4.2 cm, height: 3 cm, width: 3.3 cm) is mediolaterally and dorsoventrally compressed but anteroposteriorly elongated (Fig 1B). The neurocentral suture is faintly visible. The neural spine, prezygapophyses and postzygapophyses are well developed. The postzygapophyses bear poorly developed but distinguishable epipophyses; the diapophyseal ends are not prominently expressed but the transverse processes and the parapophyses are well formed. The costotransverse rings are present and there are no foramina on the dorsal side of the transverse processes. On the lateral sides of the centrum, pleurocoels (i.e., deep and very wide fossae occupying most of the lateral sides of the vertebral centrum) lead to deep (10 mm) fossae (one on each side) beneath the transverse processes. The ventral margin of this large fossa is a lamina-like ridge that connects the medial wall of the arcocostal lamina with the centrum. In addition, within each of the deep lateral fossae, a pneumatic foramen (2 mm) pierces the centrum in a mediodorsal direction and extends into the interior of the vertebra. On the anteroventral side of the lateral fossa, a cluster-like network of depressions is present, with each depression leading to either a small fossa or foramen (with each opening being approximately 1–3 mm).

Moving into the costotransverse rings, there are several openings (foramina) leading into the centrum dorsomedially. These openings are formed between sheaths of bone, forming bridge-like connections (i.e., like a cluster). On the inner wall of the costotransverse ring are multiple small, 1–3 mm pneumatic foramina. Three small (1 mm) foramina are present along the ventromedial margin of the right prezygapophysis. Additionally, on the anteroventral side of the centrum, between the parapophyses and posterior to the articular condyle, there are four small (1 mm) foramina. On the dorsal surface of the centrum, one pneumatic foramen (2–3 mm) is positioned at the posterior-most end of the vertebral foramen. The vertebra possesses spinoprezygapophyseal (**sprl**) and spinopostzygapophyseal (**spol**) laminae, but no other laminae.

CV4 (BRSMG Ag1174.2; length: 5.5 cm, height: 3 cm, width: 3 cm) is mediolaterally compressed with all basic landmarks being present (Fig 1A). A pneumatic foramen (4 mm) is present on the lateral side of the left arcocostal lamina. It is positioned on the posterior-most end of the arcocostal flange and its ventral margin is part of a lamina-like ridge that extends along the arcocostal lamina, connecting the anterior with the posterior margins of the flange. This foramen and the arcocostal lamina are positioned within a shallow excavation on the arcocostal flange. The foramen's dorsal border is framed by the arcocostal lamina. Between the transverse processes and the centrum is a deep fossa measuring 5 mm in depth and 8 mm in length. The fossa is anteriorly directed leading to a camellate network that contains four small (2 mm) pneumatic foramina leading farther inside the vertebra.

On the interior dorsal surface of the costotransverse ring walls there are three pneumatic foramina of 2–3 mm diameter. In addition, on the anterior part of the centrum on the right side, there is a narrow pneumatic foramen (8 mm long) that is dorsally directed and masked from its dorsal margin. On the left side, there is a fossa whose margins are part of the arcocostal lamina that extends along the costotransverse ring. The fossa is 6 mm long, directed anteriorly and might enclose foramina (which are not clearly visible). Directly dorsal to that, there is a small (1 mm) foramen on the posterior-most end of the transverse process flange. This is positioned anteroventral to the lamination located at the middle of the ventral-most margin of the neural arch. On the ventrolateral side of this lamina there is a longitudinal lamination connecting the anterior-most end of the postzygapophysis with the uppermost arcocostal lamina of the ring. Ventrally and anterior-most on the centrum there is a fossa (10 mm) with two pneumatic foramina (1 mm) around it. Midway on the ventral surface of the vertebral foramen is a narrow foramen (5 mm long) leading into the centrum. Both anterior and posterior ends of the vertebral foramen are deeply excavated and closed without any indications of aeration. The neurocentral suture is barely visible except along its anterior-most end where the fossae/foramina/laminae complex is prominently expressed.

CV5 (BRSMG Ag1174.3; length: 6.5 cm, height: 3.1 cm, width: 2.8 cm) is dorsoventrally compressed (i.e., longer than tall) with all basic landmarks present, and the neural spine is short and mediolaterally flattened. The costotransverse rings are fully formed and the ribs are present. The **sprl** and **spol** are clearly defined. On the left side there is a 3 mm long, narrow pneumatic foramen, which is triangular in outline, anteromedially directed, and whose margins are formed by two laminae. The first lamina connects the postzygapophysis with the centrum (centropostzygapophyseal—**cpol**) and continues anteriorly to meet the two arcocostal laminae (20 mm and 15 mm long) ending on the exterior surface of the costotransverse ring. These two arcocostal laminae originate from the diapophysis. The second lamina is a bony ridge extending along the centrum, which meets the arcocostal lamina anteriorly. Below the ventral-most arcocostal lamina there is a circular pneumatic foramen (3 mm) just before the posterior-most margin of the arcocostal flange. On the ventral side, hypapophyses extend medially from the anterior-most origins of the cervical ribs. A foramen (1 mm) is also present on the ventromedial side of the left parapophysis. On the right side a 1 mm pneumatic foramen whose dorsal margin is the centro-arcocostal lamina lies on the lateral facet of the neural arch. The two arcocostal laminae of the right side are 20 mm and 10 mm long respectively, and both emerge from the right diapophysis. In addition, there is a pneumatic foramen (2 mm) located on the posterior-most margin of the arcocostal flange and below the posterior-most arcocostal lamina. The anterior and posterior sides of the neural spine are deeply and narrowly excavated and blind ending. The neurocentral suture is barely visible.

CV6 (BRSMG Ag1174.4; length: 7 cm, height: 3 cm, width: 3 cm) is mediolaterally compressed (i.e., longer than tall), with all basic landmarks present. The spine is short and flattened on both lateral sides and the costotransverse rings are well formed. The diapophyses are distinct, but not very pronounced, and the parapophyses are well formed and bear weakly expressed hypapophyses on their ventromedial sides. On the left lateral side of the centrum, a 0.5 mm foramen is present. The foramen's dorsal border is a lamina that originates from the centrum and meets the convergence point of the two arcocostal laminae. The length of the lamina is 10 mm before it meets the two arcocostal laminae at its anterior-most end, which are both 20 mm long. Ventral to the lowest arcocostal lamina is a semi-enclosed pneumatic foramen, about 2 mm in diameter. Furthermore, on the medial walls of the costotransverse ring are four pneumatic foramina whose diameters are about 5 mm each facing: a) anteriorly for those foramina located on the costotransverse ring's inner surface, and b) anteromedially for those foramina located on the anterior end of the centrum. On the right side of the vertebra, the oblique posterior margin of the arcocostal flange has uneven edges, and there is only one short (2 mm long) arcocostal lamina extending from the diapophysis. Within the inner surface of the costotransverse ring there are two pneumatic foramina: one (1 mm) on the central surface of the ring and one (5 mm) on the upper medial surface of the ring, directed posteriorly within the transverse process. The lamina emerging from the arcocostal lamina extends and stops at the centre of the centrum (and always has a posterodorsal inclination). Exactly below the end of this lamina lies a 0.5 mm diameter foramen, directed anteriorly within the centrum. Two laminae are present along the lateral surface of the centrum, directed obliquely posterodorsally, which are approximately 20 mm long. On the posterior-most margin of the dorsal side of the right prezygapophysis is a 3 mm shallow fossa. Its lateral-most margin is part of a 15 mm long lamina that connects the prezygapophysis with the upper lateral surface of the neural arch at the base of the neural spine. The neural spine is flat on its lateral sides and the posterior excavation of the spinal fossa is deeper and narrower than the anterior excavation. Furthermore, two mildly expressed lamina-like ridges extend along the ventral side of the centrum. These are 30 mm long and converge midway until they reach the centre of the centrum.

CV7 (BRSMG Ag1174.5; length: 7.5 cm, height: 3 cm, width: 3 cm) has the same features as the previous vertebrae. There is a pneumatic foramen, 3 mm, on the left central lamina. Anteriorly, the central lamina reaches the arcocostal lamina. On the right side, there are no pneumatic foramina. On the upper medial surface of the costotransverse ring there are two pneumatic foramina (4 mm each) dorsally directed towards the transverse process and prezygapophysis. Another short (5 mm) arcocostal lamina emerges from the broad diapophysis. As a result, shallow canals are formed between these arcocostal laminae.

CV8 (BRSMG Ag1174.6; length: 7 cm, height: 3 cm, width: 3 cm) has all basic landmarks present. The prezygapophyses bear ridges along their dorsal surfaces. On the right lateral side of the neural spine, near its dorsal-most apex is a 3 mm pneumatic foramen with a medially directed opening that is narrow and situated parallel to the spinal margin. The passage communicates with both the anterior and posterior spinal fossae. On the same side there is a fossa (possibly enclosing a foramen) of 2 mm on the anterior part of the centrum, just anterior to the opening of the costotransverse ring. The fossa is obliquely directed anteroposteriorly, and a part of its lower margin contacts a lamina that originates from this point and extends posteriorly along the right lateral side of the centrum, stopping 15 mm from the condyle. The dorsal-most arcocostal lamina is 30 mm long. It extends from the diapophysis to the centrum-neural arch border. The lowermost lamina is 15 mm long and, as seen in some of the previous cervical vertebrae, it originates as part of the posterior edge of the arcocostal flange and continues obliquely posterodorsally until it converges with the upper arcocostal lamina. As in the other cervical vertebrae, there are foramina on the inner walls of the costotransverse rings (Fig 1C); one of them lies on the posterior end of the inner wall of the parapophysis. The foramen is anteriorly directed inside the parapophysis and is 3 mm in diameter. On the left costotransverse ring is a 3 mm pneumatic foramen on the anterior-most side of the centrum, within the ring. In addition, on the left side there are three arcocostal laminae in almost parallel formation, with a foramen (1 mm) on the ventral-most bony shaft, formed between the two lower arcocostal laminae. Also, anterior to the left costotransverse ring, on the proximal-most side towards the condyle, there are two foramina, each 0.5 mm, which are posteriorly directed inside the centrum. Their margins are poorly defined. On the left hypapophysis there are two foramina, both 1 mm, which are connected by a septum. They are aligned along the anterior facet of the hypapophysis. In addition, four small foramina are present along the posterior facet of the hypapophysis, each 0.5 mm in diameter, and located just ventromedial to the rib.

CV9 (BRSMG Ag1174.7; length: 7.3 cm, height: 3.5 cm, width: 3.4 cm) has all basic landmarks and is mediolaterally compressed. The neurocentral suture is apparent and closed (Fig 1D). On the left side there is a pneumatic foramen (3 mm) directed anteromedially within the centrum. The foramen's margins are sharp and well defined. A pneumatic foramen (2 mm) penetrates the inner wall on the upper side of the left costotransverse ring. In addition, a pneumatic foramen invades the posterior lateral surface of the centrum, as well as the inner ventral surface of the costotransverse ring. These foramina range from 2–4 mm in diameter. There are five foramina in the left ring and one foramen in the right ring. Those pneumatic foramina located in the left ring form an interconnected septated cluster. There is also a pneumatic foramen inside the vertebral foramen on the inner ventral surface. The foramen is about 3 mm in diameter, narrow, and directed ventrally inside the centrum.

CV10 (BRSMG Ag1174.8; length: 7.7 cm, height: 3.5 cm, width: 3 cm) retains all of the basic characteristics of the aforementioned vertebrae. On the left side, a pneumatic foramen (3 mm) invades the centrum and its dorsal margin is bordered from the arcocostal lamina that extends along the centrum. Three arcocostal laminae on the arcocostal ring's exterior surface converge into one lamina. They are 15 mm, 20 mm, and 25 mm long, respectively. On the right side, a smaller pneumatic foramen penetrates the centrum (2 mm). In addition, two arcocostal laminae converge at a point just before they meet this central pneumatic foramen. From each posterior end of the hypapophyses extends a 15 mm long lamina along the ventrolateral side of the centrum. This central lamina begins from the anterior-most section of the centrum, in the shaft between the parapophyses, and extends for 30 mm until it bifurcates 20 mm before the posterior-most end of the cotyle. The two laminae follow the lateral and distal margins of the cotyle. Pneumatic foramina are also present in the inner dorsal, medial, and ventral walls of the costotransverse ring. In the left ring, there are one ventral, two lateral, and three dorsal pneumatic foramina, all having an anterior invasive direction, ranging from 1–3 mm. In the right costotransverse ring, there are one ventral, one dorsal and two lateral pneumatic foramina, all of 0.5 mm. On each hypapophysis, there is a pair of septated pneumatic foramina that are each 2 mm directed dorsolaterally within the hypapophyses. One more pneumatic foramen lies on the posterior-most margin of the left postzygapophysis. It is 1 mm wide and anteriorly directed inside the postzygapophysis. All mediolaterally compressed and, therefore, elongated cervical vertebrae bear well-formed ribs that reach up to 40 mm in length and that are directed posteriorly and slightly laterally (Fig 1E).

CV11 (BRSMG Ag1174.9; length: 7.5 cm, height: 3.5 cm, width: 3.4 cm) has no pneumatic foramina on the left lateral side, but there is one foramen (0.5 mm on the right side that has a prominent anterior margin. The foramen is deep and directed anteriorly. Dorsal to this, lies the posterior end of the centroarcocostal (also known as centrodiapophyseal) lamina. There are pneumatic foramina in the inner dorsal wall of the costotransverse rings (2–3 mm). More specifically, there are four pneumatic foramina (each 2 mm) aligned in an oblique row on the ventrolateral surface of the right ring's inner wall, directed medially-anteromedially. The hypapophyses are slightly damaged, revealing septated, camellate trabeculae (an aerated network created by air sac diverticula) within the vertebra. On the left side, three arcocostal laminae exist, being 17 mm, 25 mm, and 30 mm long, respectively. They do not converge, but there is a depression between the lower two laminae, forming a recessed shaft between them (a characteristic that is variably expressed in cervical vertebrae). On the right costotransverse ring there are two laminae with a deep shaft between them. They are parallel to each other and terminate on the posterior side of the arcocostal flange; the ventral-most lamina shifts from posterior to posterodorsal direction until it meets the upper lamina. The postzygapophyses are broad and dorsoventrally flattened. In addition, the centrohypapophyseal laminae (**chpl**) are present as well. Another attribute that starts to be more expressed from this vertebra onwards is that the neural spine is mediolaterally flat but tall (dorsally elongated), which stands in contrast to the previous vertebrae.

CV12 (BRSMG Ag1174.10; length: 7.5 cm, height: 4 cm, width: 3.5 cm) bears all basic landmarks and its pre- and postzygapophyses are dorsoventrally flat (Fig 1F). The left side of the vertebra has no pneumatic foramina, and the lateral surface of the costotransverse ring shows three laminae (20 mm, 25 mm, and 25 mm long, respectively). These three laminae unite at the middle of the centrum. There is a 20 mm long lamina exactly below the centrum, directed obliquely anteroposteriorly. On the ventral side, there are two foramina on the anterior-most end of the centrum directly beneath the condyle. These foramina are aligned along the horizontal axis of the vertebra and are 1 mm and 2 mm, respectively. The right lateral side of the centrum does not bear foramina. On the inner ventral surface of the vertebral foramen is a foramen (3 mm) that penetrates the bone in a ventral direction. On the left inner wall of the vertebral foramen, two foramina (1 mm and 2 mm) penetrate the bone in an anterolateral direction. Inside the left costotransverse ring, six pneumatic foramina cover the inner ventrolateral surface, ranging from 1–4 mm in size, which are directed anteriorly within the bone. Two of them merge, forming a small fossa leading to further network-like cavities within. In addition, a dorsal pneumatic foramen penetrates the transverse process. The right costotransverse ring houses a dorsal foramen penetrating the bone anterolaterally and a lateral (1 mm) foramen on the central wall penetrating it medially; three more foramina penetrate the inner wall of the parapophysis being 1 mm, 2 mm, and 2 mm, respectively. There is one more foramen (2 mm) on the inner wall of the costotransverse ring. No laminae appear to exist within the rings. The vertebral foramen bears shallow fossae (each 3 mm) on the right lateral wall. The ventral surface of the centrum possesses laminae extending along its anteroposterior plane. Finally, the diapophyses of this vertebra are more strongly expressed with rugose surfaces; they are triangular in shape and extend posteriorly with the arcocostal laminae aligned in parallel with the distal ends of the diapophyses, thus forming a greater triangular area where they all meet on the central suture.

CV13 (BRSMG Ag1174.11; length: 8 cm, height: 4.3 cm, width: 4 cm) has all previous features and basic landmarks, including the parallel arcocostal laminae. There is a deep pneumatic foramen on the dorsal inner wall of the vertebral foramen that is covered by a fold in its anterior margin. The foramen is 3 mm in diameter, and 10 mm anterior to this is another foramen (15 mm) that communicates internally with the previous one. The posterior-most of the two foramina is directed anteriorly within the bone, whereas the anterior-most penetrates the bone in a dorsal direction. Further along the vertebral foramen, on its inner lateral side, are five foramina (each 1 mm in diameter), and on the anterior and ventral inner walls are three foramina (each 1 mm) that penetrate the centrum. A 3 mm long shaft depresses the middle of the cotyle. On the right costotransverse ring, facing it anteriorly, a 5 mm wide pneumatic foramen penetrates the transverse process deeply in a posterior direction. On the ventral inner wall of this ring, two foramina (each 3 mm) are present and are directed anteroventrally. A foramen (2 mm wide) invades the inner lateral wall of the right costotransverse ring in a laterodorsal direction. In the left ring is a 10 mm wide pneumatic foramen exactly ventral to the posterior margin, penetrating the transverse process vertically. There is also a 2 mm foramen on the lateral wall of the centrum, two foramina (each 1 mm wide) on the inner wall of the ring, and three foramina (each 1–2 mm) penetrating the posterior aspect of the left parapophysis. On the right side, at the far posterior end of the centrum lies a 2 mm wide pneumatic foramen that penetrating medially on the lateral side, just anterior to the lower margin of the cotyle. No lamination is associated with the foramen.

CV14 (BRSMG Ag1174.12; length: 8 cm, height: 4.5 cm, width: 4.5 cm) has a shorter and anteroposteriorly inclined neural spine, with its anterior margin being taller than its posterior one. The pre- and postzygapophyses are dorsoventrally compressed. On the anterolateral side of the left diapophysis there is a fossa (3 mm in depth) leading to a foramen (1 mm). There are four main arcocostal laminae on the left ring. The first two (dorsal-most) are parallel to each other and their lengths are 25 mm each. The two ventral-most laminae are oblique, non-parallel, and converge at the end of the ring's flange. Their lengths are 10 mm and 15 mm, respectively. In addition, on the same side there are three small foramina (each 1 mm in diameter) on the inner dorsal wall of the left ring, penetrating the transverse process. On the posterior and posteroventral facets of the left parapophysis inner wall there are multiple clusters of foramina ranging from 1–3 mm in width, which seem to communicate with each other within the bone. On the right ring, there are multiple small foramina (each 1 mm wide) on the posterior and ventral inner sides. On the right lateral side at the convergence of the one (and only) arcocostal lamina with the posterior end of the costotransverse flange there is a small foramen (1 mm diameter) that is directed posteriorly. The lateral and ventral facets of the vertebral foramen have each a foramen. The lateral foramen is 1 mm wide and the ventral foramen is 2 mm in width. The lateral foramen invades the bone in a posteromedial direction, while the ventral foramen invades it in a dorsal direction. On the posterior-most end of the dorsal facet of the neural canal, at the middle of the dorsal margin of the condyle, lies a circular fossa (3 mm) that leads into a foramen. The foramen is positioned within the fossa's inner left lateral side. Lastly, the hypapophyses' medial curvature is greater than in the previous vertebrae.

CV15 (BRSMG Ag1174.13; length: 8 cm, height: 4.5 cm, width: 4.5 cm) has an obliquely elevated spine (see CV14). On the left side, three arcocostal laminae (20 mm, 25 mm, and 20 mm long, respectively) lie on the costotransverse ring's lateral surface. The first two are parallel while the third is dorsally directed. There is a foramen (1 mm wide) on the lateral side of the vertebra, located posterior to the left parapophysis. The foramen's ventral margin is a ridge that extends posteriorly along the cervical rib. Ventral to and posterior to the transverse process is an anteroposteriorly long and narrow pneumatic foramen (10 mm long). It is slightly oblique and deep with a mediodorsal direction. No lamina is associated with the foramen. From the middle of the dorsal and ventral margins of this foramen, bony septa-like extensions emerge and reach each other from their opposite sides. Multiple foramina are present on the dorsolateral inner surface of the left costotransverse ring. They range from 2–3 mm in width and, within these foramina, deeper excavations and foramina are revealed that aerate the bone internally. In addition, similar cluster-like networks of four foramina (2–3 mm in diameter) are present on the posteroventral surface of the same ring. On the right costotransverse ring, a narrow and oblique pneumatic foramen (10 mm long) penetrates deep into the transverse process. Further along the dorsal surface are around nine foramina ranging from 1–3 mm that aerate the transverse process in a dorsolateral direction. On the ventral wall of the ring, posteroventral to the right parapophysis are three pneumatic foramina (1–2 mm in width) leading to further pneumatization within their cavities. Inside the ring, on the ventral inner wall there is a 3 mm wide foramen backed by a 3 mm wide fossa. The foramina within the ring are located on a roughened surface. On the ventral inner wall of the vertebral foramen are two pneumatic foramina lying beside each other (2 mm and 3 mm). No laminations are associated with the latter foramina. On the posterior and lateral sides of the centrum, just before the posterior margin of the cotyle, is a 1 mm wide foramen. On the lateral walls of the spinal process, there are multiple fossae (2–3 mm) and a foramen (1 mm wide) positioned on the anterior and posterior ends. They invade the spine and are associated with the left spinopostzygapophyseal lamina (**spol**). Finally, there is a 1 mm wide foramen on the anterior recess of the spine, which seems to invade it.

CV16 (BRSMG Ag1174.14; length: 8 cm, height: 5.5 cm, width: 5.5 cm) has well-defined landmarks and is characterised by reduced rib length (15 mm). The vertebra possesses multiple foramina (sizes range from 1–4 mm) within each ring, internally connected into a complex network. This network forms a rough surface that results from numerous septated and interconnecting foramina. They cover the dorsal and lateral inner walls of the costotransverse rings. On the ventral surface of each parapophyses several foramina (measuring up to 10 mm each) lie within fossae, and these complexes are anteroposteriorly directed within the parapophyses. Their margins are prominent. The postzygapophyses and the transverse processes are deeply aerated further within the centrum. On the left lateral side, ventral to the two arcocostal laminae, lies a 5 mm wide foramen that is directed posteromedially and has well defined margins. Posterior to the posterior end of the arcocostal flange are two pneumatic foramina (each 2 mm wide) that are directed medially. On the medial surface of the right spinal wall is a 0.5 mm foramen with its dorsal margin formed by the right spinopostzygapophyseal lamina (**spol**). Furthermore, on the right lateral side, there is a pneumatic foramen (1 mm wide) at the convergence of the arcocostal laminae that is directed medially within the centrum. A camerated opening is present ventral to the right transverse process, forms a narrow chasm (directed dorsomedially) and extends posteroventrally for 10 mm. No laminae are associated with this formation. On the ventral inner side of the costotransverse ring, there are numerous foramina (each 0.5 mm in width) that are directed ventrally-anteroventrally within the parapophyses. On the posterior-most margin of the centrum, before the lateral right margin of the cotyle, there are two shallow fossae that are each 2 mm. The arcocostal laminae on each ring are parallel to each other and measure 20 mm and 15 mm long, respectively. On the ventral side of the vertebral foramen are three foramina (3 mm, 2 mm, and 1 mm in width, respectively) each following an anteroposterior direction. The central lamina along the ventral side is not present on this vertebra. Centrohypapophyseal laminae (**chpl**) are also present, and it is evident that the hypapophyses are also internally aerated.

CV17 (BRSMG Ag1174.15; length: 5.5 cm, height: 6 cm, width: 6 cm) is expanded mediolaterally, but the posterior part of the vertebra is missing. The cut surface reveals the internal trabeculated network. The estimated total original length of the vertebra is approximately 7–9 cm. Despite the missing posterior part, this vertebra exhibits well-defined landmarks. The neural spine is tall and the distance between the prezygapophyses is broad. The hypapophyses are very well formed, rather short and broad, and oriented anteroventrally and laterally. On the ventral side between the **chpl** are two foramina, which are 1 mm and 2 mm, and are directed posteromedially. In addition, mediolateral to the left **chpl** is a 3 mm wide foramen that is directed dorsally within the centrum. The foramen's margins form a lamina-like network that bifurcates around the foramen and then reunites further posteriorly along the centrum. A fossa (2 mm wide) lies on the midline of the condyle ventral surface and leads to two laterally directed foramina (each 1 mm) that are connected via a septum. The left lateral side shows a ventromedially directed pneumatic foramen (3 mm) that is located posterior to the costotransverse flange. There is a deep opening ventral to the transverse process, which is 5 mm in diameter, 15 mm long, and aerates dorsally-posterodorsally the centrum and transverse process. As always, the diapophyses bear two arcocostal laminae each, whose lengths are 20 mm and 10 mm, respectively. The lateral margin of the left prezygapophysis has a 1 mm wide foramen directed obliquely ventromedially. On the right side, exactly posterior to the conjunction of the arcocostal laminae on the centrum, is a cluster of foramina (20 mm across) that reveals further inner camellations and lead into smaller fossae and foramina. These are connected ventrally via septa with another narrow foramen (20 mm long), which is obliquely directed and has further camellations that lead to posteroventral inner aerations of the centrum. A long, narrow foramen beneath the transverse process is also present in this vertebra. It is 20 mm long, has well defined expressed dorsal margin, and extends deeply in a dorsal direction within the transverse process. On the ventral surface of the vertebral foramen lie numerous pneumatic foramina ranging in width from 1–3 mm. Finally, on the ventromedial side of the left parapophysis lies a 2 mm wide foramen, which is dorsally directed, that reveals the internal trabeculated network. All vertebrae except CV16 and CV17 possess a faint lamina, identified as the *crista ventralis*, extending along the middle ventral side of the centrum.

The mounted skeleton of an adult *Struthio camelus* individual (BRSMG Af962) was also studied, but could only be examined from its right side. The neural arches are firmly attached to the centra, with only minor fissures apparent. The locality for the specimen is unknown, but the skeleton is in excellent condition; all of the bones are in place, except for the second sternal rib on the right side and the fifth sternal rib on the left side, which are missing. Its dimensions are 2.2 m in height and 1.3 m in length. As viewed from the right, CV2 (axis) and CV13–18 bear external evidence of pneumaticity. CV2 has a pneumatic foramen ventral to the arcocostal lamina on the costotransverse ring exterior surface. This lamina extends for about 20 mm in a posteroventral direction. The cervical ribs are well formed, extend posteriorly and are attached to the posterior ends of the diapophyses; from CV3 onward the ribs become increasingly elongated. The same observation applies to the neural spines. Posterior to every neural spine is a deep and narrow excavation that is directed anteriorly within the spine, forming a triangular 3D shape with obliquolateral sides (the sides are directed medially). The dorsal prominences of the margins resemble laminae-like ridges. The spinopostzygapophyseal laminae (**spol**) are present. The excavations at the back of the neural spine seem to be aerated because they contain pneumatic foramina.

On CV13, a foramen is positioned ventromedially on the diapophysis. The foramen is directed slightly posteriorly and it is approximately 2–3 mm in diameter. Next to that foramen and anterior to the hypapophysis there is a larger hole (possibly a break) that reveals two, 1–2 mm wide each, pneumatic foramina, showing that the cervical vertebrae were internally pneumatized by diverticula. Furthermore, four arcocostal laminae extend posteriorly from the diapophyses. The diapophyses curve ventromedially. The costotransverse rings are well formed and the transverse processes are prominently expressed along the length of the vertebra. The ridge-like arcocostal laminae on the lateral sides appear to show depressions on their anterior-most margins. There is also an opening (1 mm wide) on the anterodorsal facet of the right prezygapophyses, a trait that can possibly be considered as another indication of air sac invasion. On CV14, there is a foramen (2 mm wide) ventral to the ventralmost arcocostal lamina. Anteriorly, a fossa (5 mm long) extends dorsally along the anterior side of the neural spine. The fossa is 2 mm deep but it does not communicate with the postspinal fossa. Laminations do not extend from its anterior margins. On CV17, there are three pneumatic foramina (10 mm in diameter and 10–20 mm deep) on the lateral side of the centrum. On the dorsal surfaces of CV14–18, there are two shallow fossae, one on each side originating from the prezygapophysis. They are approximately 2–3 mm deep, and the fossae margins are laminae that connect (a) the spine with the prezygapophyses and (b) the postzygapophyses with the prezygapophyses. A general observation was that the cervical vertebrae are not as pneumatic as the dorsal vertebrae with the latter possessing many more foramina and fossae than the cervicals.

### Summarized observations

Three main skeletal features (foramina, fossae, and laminae) associated with PSP were recorded in the avian taxa examined.

#### Vertebral foramina

In summary, foramina created by the penetration of diverticula into the cortical bone ranged in diameter from 0.5–10 mm. Foramina pierced the bones in a variety of positions. The foramina were found both singly and in clusters with numbers ranging from 2–9 with the openings interconnected by septa. The margins were frequently associated with vertebral laminae or the foramina were positioned within a fossa.

**Fig 2:**
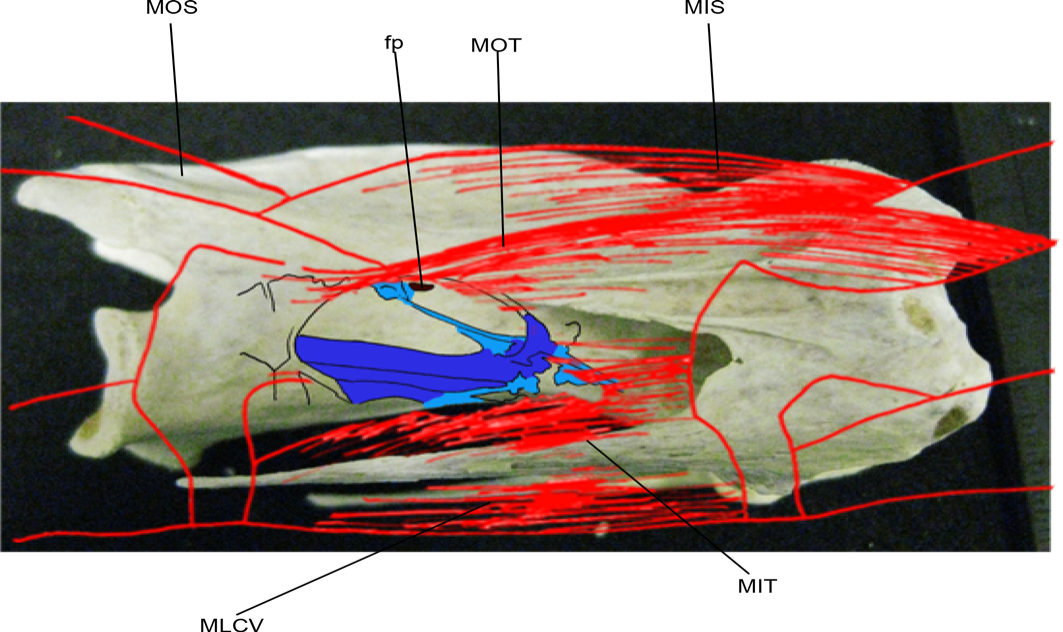
Schematic representation of air sac complex with its associated muscles. Right lateral view of a *Struthio camelus* (ostrich) mid-cervical vertebra, showing a local fraction of the air sac diverticular complex and the muscle groups (red) associated with it and the vertebra. The black lines represent the various borders of the diverticular membrane extensions; the purple shows the air sac's lateral vertebral diverticulum along the corpus; the light blue depicts the membranes that extend from the diverticulum, anchoring on the *corpus* and invading the brown *foramen pneumaticum*. Abbreviations: fp *foramen pneumaticum*; MOS *M*. *obliquospinalis*; MOT *M*. *obliquotransversalis*; MIS *M*. *interspinalis*; MLCV *M*. *longus colli ventralis*; MIT *M*. *intertransversalis*.

Positions in which foramina were identified include:

i) The dorsal surface of the transverse processes (present in that location only in rhea and ducks).
ii) The lateral or ventral surface of the centrum, termed the *foramen vertebrale laterale* or *foramen vertebralis ventralis*, respectively. Such foramina were present in all examined taxa, except the grebe and loons.
iii) Within the bounding wall of the vertebral foramen (their characteristics fall under pneumatic and not neural or vascular specifications), positioned either dorsally, reaching into the neural arch, or ventrally, reaching into the centrum. Those foramina reaching into the centrum sometimes communicated with the *foramen vertebralis ventralis*. Ostrich (Figs 1 and 2) and rhea vertebrae possessed these features.
iv) Anteroventral or posteroventral to the transverse process, usually forming small clusters of foramina. These features were present in all of the taxa examined.
v) On either side of the neural spine. Such foramina were present in most birds examined except loons, the grebe, penguins, tinamous, and kiwis.
vi) Within the posterior depression of the neural canal, being present either singly or in clusters (found in all birds examined).
vii) On the inner wall of the costotransverse ring (termed the *foramen transversorium* within the *ansa costotransversaria*) as single, paired or clustered foramina (found in all taxa examined).
viii) Near to the *lamina arcocostalis*. In all cases the foramen margins were formed by vertebral lamina(e) (found on all the large ratites, i.e., ostrich, rhea, moa, and cassowary).
ix) On the neurocentral suture line. This condition occurs rarely in the ostrich and rhea.
x) At the anterior and posterior ends of the centrodiapophyseal laminae (found in all ratites).
xi) On the *crista ventralis* (hypapophysis) in ratites and penguins.
xii) Near or adjacent to the centrohypapophyseal lamina in ratites.
xiii) On the ventral surface of the corpus between the *processus costarius* (parapophysis) in the large ratites.
xiv) On the lateral or ventral sides of the condyle (*facies terminalis cranialis*) in the ratites and ducks.
xv) On the ventral surface or the posterior rim of the cotyle (*facies terminalis caudalis*) in the large ratites.
xvi) On the surface of either the prezygapophyses and/or postzygapophyses in the large ratites.
xvii) Between the neural arch and the postzygapophyses in the large ratites.

Observations (xiv) and (xv) demonstrate that pneumatic foramina are located just anterior to the *foramen intervertebrale*. This occurred more often on the thoracic and synsacral vertebrae.

#### Vertebral fossae

Fossae are shallow or deep depressions on the vertebrae that do not enter the bone cortex. In the taxa examined, they range from 5–30 mm in width and depth. Large fossae are present on the synsacral vertebrae of the large ratites. Usually, fossae house foramina and/or are adjacent to vertebral laminae, or are defined by the development of prominent laminae. The margins of the fossae vary in prominence, from shallow changes in the slope of the vertebral surface, to gently curved ridges, through to the development of distinct, sheet-like vertebral laminae. As observed by the dissection of the ostrich neck, the margins of these fossae serve as muscular and/or air sac membrane attachments (described in more detail below).

Observations showed that fossae were located:

i) On the lateral sides of the corpus in all of the birds examined, except the grebes.
ii) Ventral to the *processus transversus* in all of the birds examined.
iii) Between two arcocostal laminae, or between one such lamina and the arcocostal posterior flange in large ratites.
iv) On either side of the *arcus vertebrae* (found only in the ostrich, rhea, emu, and cassowary).
v) Between the spinodiapophyseal and the prezygodiapophyseal laminae in the ostrich, rhea, and emu.
vi) On the lateral surfaces of the *processus spinosus* in all of the birds examined.
vii) Anterior to the *processus transversus* at the point where it connects with the centrum (found in all birds examined, except the grebe and the loons).

In addition, relatively large fossae were found to encompass smaller fossae within them. This usually occurs around the junction of the transverse processes with the corpus, within the spinal fossa(e), or within the inner walls of the costotransverse ring.

#### Summary of pneumatic features

We identify seven pneumatic phenomena in birds (Tables 2–5, S1 Table and S3–S13 Tables). Apart from the three expected pneumatic features (pneumatic foramina, fossae, and laminae) that were commonly present in most AUs of the vertebral column in most birds studied, four more were recorded in the form of combinatory elements. These were: (i) pneumatic foramina associated with laminae, (ii) fossae associated with laminae ('laminated fossae'), (iii) fossae that incorporated foramina within them, and (iv) septated foramina. The latter characteristic appeared as a cluster of foramina interconnected by thin sheaths of bone. These sheaths are not to be confused with laminae. These seven features were found in the various aforementioned positions (see section 'Vertebral fossae', above). Presence of a pneumatic element in even one vertebra was sufficient to score it as present (+) for the entire AU.

In summary (Tables 2–5 and S3–S7 Tables), the large ratites (ostrich, rhea, emu, cassowary) possess numerous pneumatic foramina, fossae, and vertebral laminae and also exhibit many complex combinations of these characters (e.g., presence of sub-fossae, setting of foramina within fossae, and close associations of laminae and foramina/fossae). All of these features are present, but developed less strongly, in the moa, and are also present, though weakly expressed, in the kiwis and tinamous. All features are most frequently present in the cervical and thoracic vertebrae, whereas the synsacral and caudal vertebrae exhibit reductions in the expression of the more complex character combinations, but the three basic features (foramina, fossae, and laminae) are retained.

Although kiwis and penguins are generally considered apneumatic, it appears that this is not strictly the case. Kiwis possess some pneumatic foramina, as well as fossae, laminae, and 'laminated fossae' on their cervical vertebrae (Table 2 and S8 Table), while their thoracic and synsacral vertebrae exhibit foramina (it is not possible to ascertain whether they are vascular, neural, or pneumatic; dissection is required). Another notable observation is the presence of the three basic pneumatic characters plus one complex character in the cervical column of all the examined penguins (Tables 2–5 and S10 Table) even though they are deep divers and have a robustly constructed skeleton. The expression of these features is limited and is not present in all vertebrae. The moa does not exhibit all of the vertebral characters considered, but it does possess many of them (see Tables 2–5 and S4 Table), and damaged cervical vertebrae reveal extensive trabeculae formed by the bony septa that subdivide camellated bone due to aeration from an invading pneumatic diverticulum. The vertebral characters of poor flyers (loons, grebe, and tinamous) are similar in many respects to those of the non-flying penguins and kiwi. These taxa are also similar in terms of body size.

Foramina, fossae, and laminae are most frequently present in the areas where the transverse processes meet the centrum, in the spinal canal and adjacent areas, and within the inner surfaces of the costotransverse ring. Strong associations between laminae and either foramina or fossae are found most frequently among large ratites, especially within the cervical and thoracic regions of the vertebral column. The presence of septated foramina is variable and does not appear to be related to either the size or locomotor mode of the birds. Rare but noteworthy occurrences of foramina, fossae, and laminae are found on the epipophyses (*tuberculum dorsale*), prezygapophyses and postzygapophyses, hypapophyses (*crista ventralis*), parapophyses (*processus costarius*), and on the ventral surfaces of the cervical and thoracic vertebrae. These rare occurrences are more often observed in the cervical and (less frequently) thoracic vertebrae of the ostrich, rhea, emu, kiwi, and tinamou. The vertebral laminae observed most frequently in association with pneumatic foramina are the prezygodiapophyseal (**prdl**), prezygoparapophyseal (**prpl**), arcocostal, centrohypapophyseal (**chpl**), spinopostzygapophyseal (**spol**), and centrodiapophyseal (**cdpl**) laminae [54]. Fossae are usually associated with the spinoprezygapophyseal (**sprl**), spinopostzygapophyseal, postzygodiapophyseal (**podl**), and postspinal laminae.

### Association of soft tissues and vertebral structures in the ostrich neck

**Fig 3:**
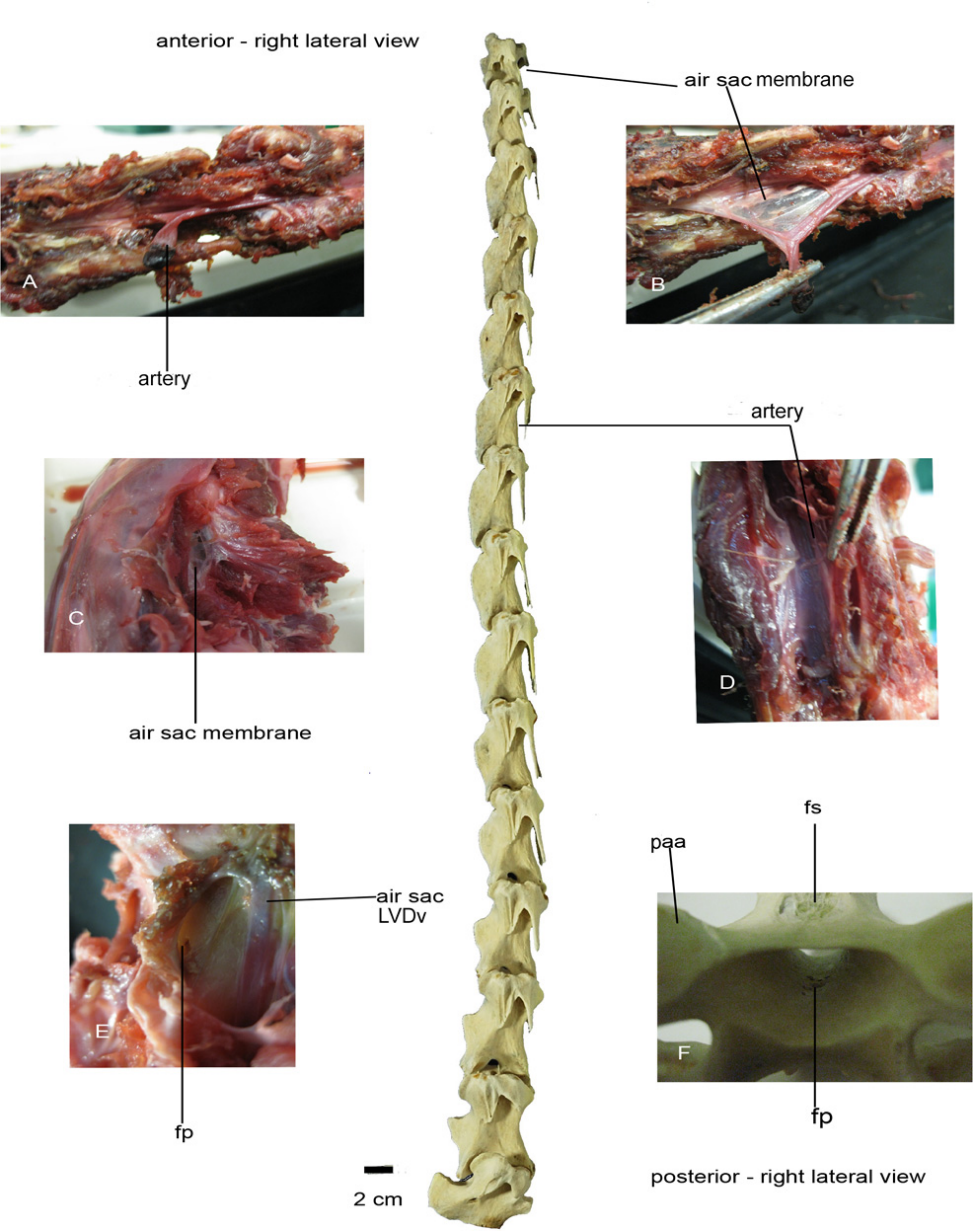
Right lateral view of the ostrich neck with flesh depictions. **(A)** Anterolateral view of the anterior part of the neck revealing the free end of the cervical artery; **(B)** Same for (a) but with the artery extended revealing its associated semi-transparent diverticular membrane that attaches within the muscle layers; **(C)** Partially dissected ostrich neck with some of the exterior muscle layers stretched out showing the intertwined air sac membranes; **(D)** Image exposing the interior surface of the cut-open cervical artery located on the ventral side of the cervical column; **(E)** Lateral view of a cervical vertebra exposing the air sac lateral vertebral diverticulum and its transparent extensions invading the *foramen pneumaticum* positioned dorsally to the lateral vertebral diverticulum; **(F)** Posterior view of a cervical vertebra showing a cluster of 3–4 pneumatic foramina within the foramen vertebrale inner ventral wall. Abbreviations: paa *processus articularis caudalis*; fs *fossa spinalis*; fp *foramen pneumaticum*; LVDv lateral vertebral diverticulum. The scale bar corresponds to the main cervical column.

**Fig 4:**
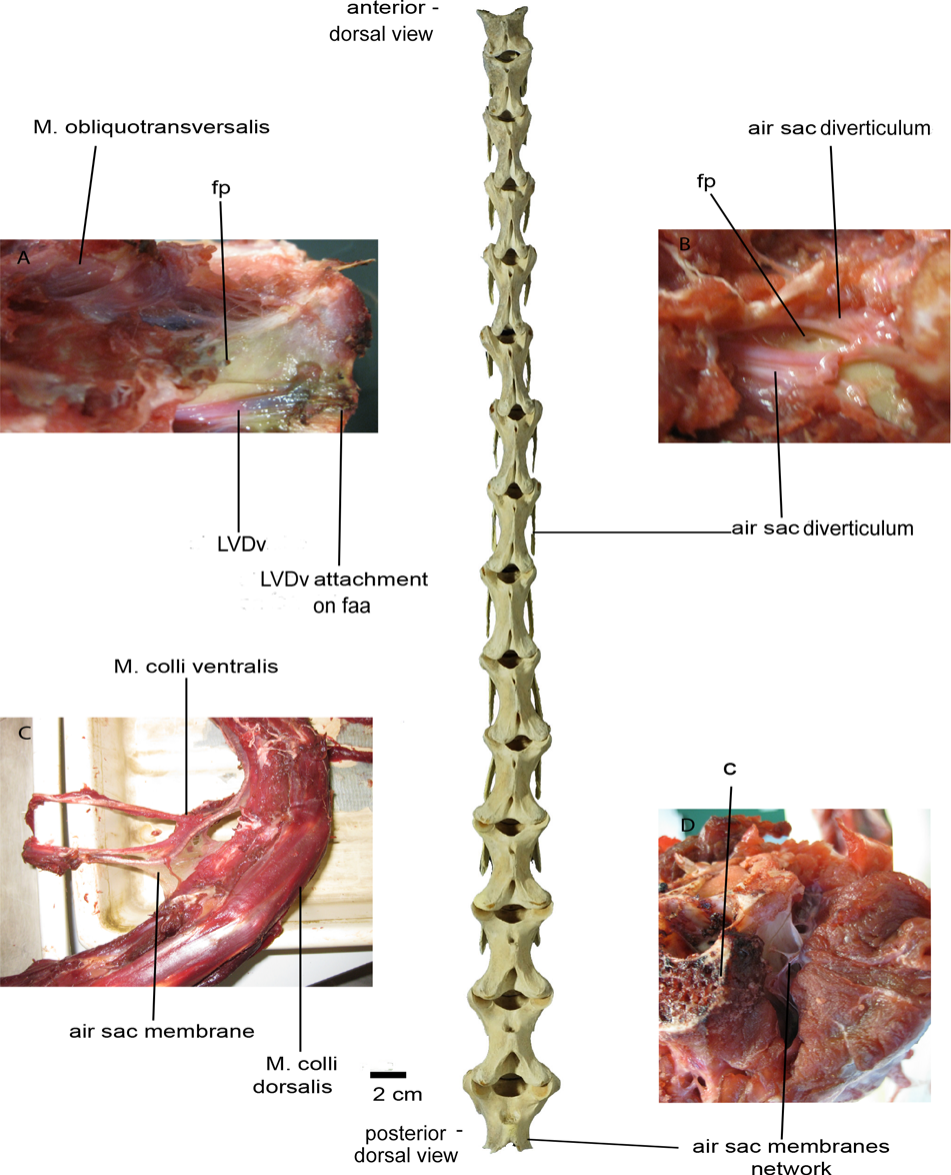
Dorsal view of the dissected ostrich neck with dissection captions. **(A)** Anterolateral view of a cervical vertebra revealing a *foramen pneumaticum* on the *corpus* and the purple air sac's lateral vertebral diverticulum (LVDv) that extends along the *corpus* and attaches on the posterior margin of the *facies terminalis caudalis*. Note the obliquotransverse muscle (*M*. *obliquotransversalis*) anterodorsally to the foramen as well as the transparent air sac diverticular extensions that attach on the foramen's margin and invade it; **(B)** Another close-up from another cervical vertebra showing a pneumatic foramen directly above the air sac diverticulum (LVDv) and below the air sac membrane attachment; **(C)** Part of the ostrich neck before the removal of the exterior muscles. Note the transparent air sac membrane that folds beneath the muscle complex. The membrane system is a continuous network that engulfs the bone and is covered by the muscular system; **(D)** Right posterolateral view of the last cervical vertebra revealing the air sac membrane network anchoring laterally to the muscular system and medially to the *corpus*. Scale bar corresponds to the main cervical column. Abbreviations: c *corpus*; fp *foramen pneumaticum*; faa *facies articularis caudalis*; LVDv lateral vertebral diverticulum.

Associations between the cervical air sacs (*saccus cervicales*), their diverticula (*diverticula vertebralia*; [65]), cervical muscles, and osteological features (sites of air sac/muscular attachments, pneumatic foramina, fossae, and laminae) were examined during the ostrich dissection. Ridge-like bony rugosities are present on the exterior surfaces of the costotransverse process, the costotransverse ring, the arcocostal surface, the anterior surfaces of the prezygapophyses, diapophyses, and parapophyses, as well as on the dorsoposterior surface of the postzygapophyses. Four main muscle groups are identified attaching to these rugosities and the surfaces of the *processus spinosus*: (a) *M*. *obliquospinalis*, (b) *M*. *obliquotransversalis*, (c) *M*. *interspinalis*, and (d) *M*. *intertransversalis* (Fig 2). The *M*. *obliquospinalis* extends posteriorly and obliquely from both lateral surfaces of the *processus spinosus* until it reaches the next vertebra and anchors itself on the same points. The *M*. *obliquotransversalis* is attached to the *corpus* lateral surfaces and extends posteriorly to attach to the *processus transversus* of the posterior vertebra. The *M*. *interspinalis* is attached to the dorsal surface of the *processus spinosus* and extends posteriorly to the next. Finally, the *M*. *intertransversalis* extends from one *processus transversus* to the next. O'Connor [28] identified and summarized the different diverticular portions of the air sacs that span along and through the vertebral column and girdle systems of birds. Here in our study, the main, paired cervical air sac extends along the dorsal side of the cervical column, measuring about 10 mm in diameter, and its main originating diverticula are approximately 5 mm in diameter. The cervical air sacs extend circumferentially around the cervical vertebrae via their interconnected diverticula, reaching the ventral side of the column. According to O'Connor [28], the diverticula extending laterally along the centra and through the vertebrarterial canal are the lateral vertebral diverticula (LVDv). Moreover, the shorter and “dorsally directed outpocketing originating from supramedullary or supravertebral diverticula that expand within the epaxial musculature” ([28]:1205) are the intermuscular diverticula (IMDv) and “the longitudinal system variably occupying the extradural space within the vertebral canal” are the supramedullary diverticula (SMDv) ([28]:1205). Furthermore, the “short, segmental expansions from the SMDv that occupy a position on the dorsal surface of the vertebral neural arches” are the supravertebral diverticula (SVDv) ([28]:1205) and, finally, “the short, segmental connections between two or more longitudinal diverticular networks (e.g., between the LVDv and SMDv)” are called anastomosing diverticula (AnDv) ([28]:1205). The LVDv (also known as *canalis intertransversarius*; [26]) extend along the entire length of the neck (Figs 3B, 3E, 4A and 4B), passing through the *foramen transversarium* of each vertebra (i.e., through the *ansa costotransversaria*). They extend via membranes that engulf the entire circumference of each bone's surface including the ribs, i.e., the main air sac diverticula give rise to membranous extensions that surround the vertebrae.

**Fig 5:**
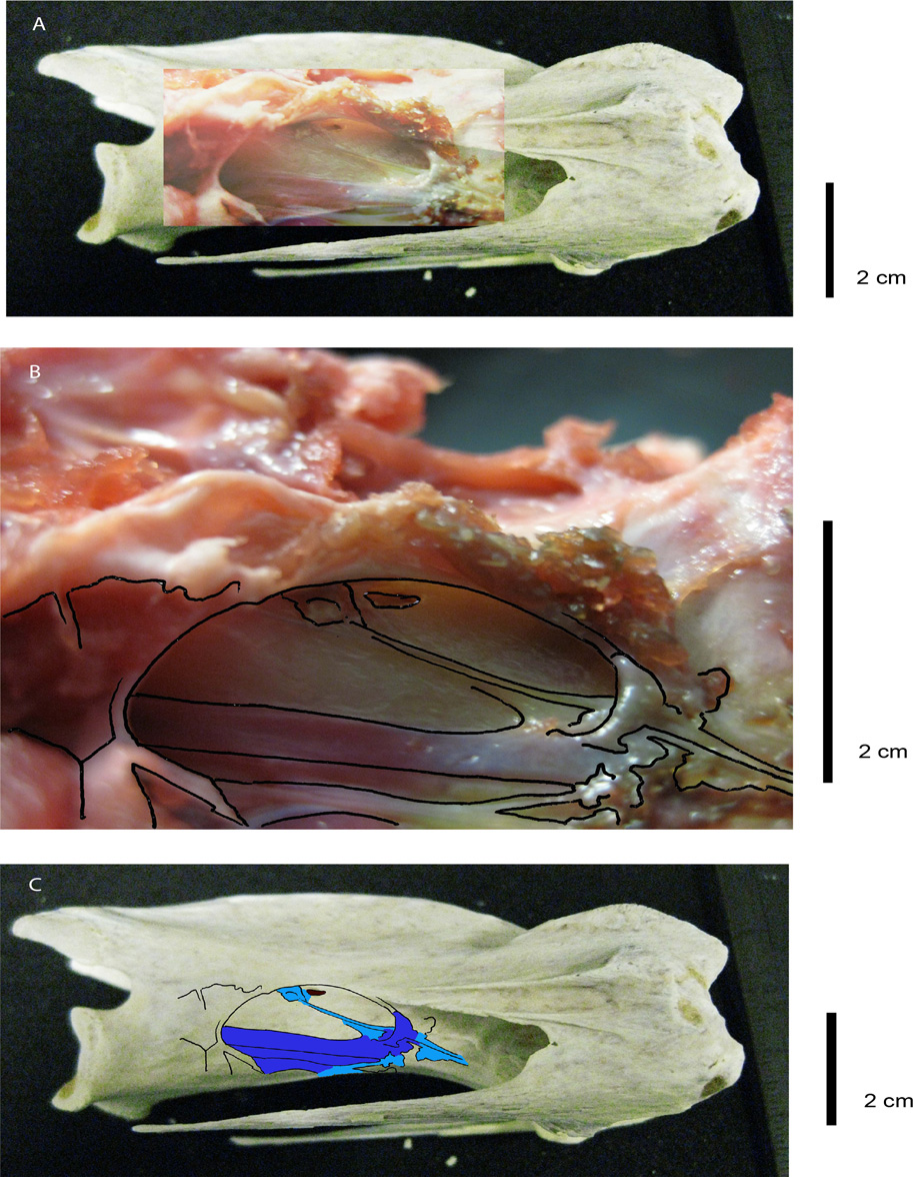
Triple schematic of ostrich vertebra-air sac-flesh tissue system. **(A)** Depiction of a semi-transparent layer of the actual close-up of the air sac diverticulum system with its surrounding tissue placed on top of the cervical vertebra; **(B)** Magnified air sac diverticulum and its membranous extensions. The **black** lines indicate the various folds and borders of the air sac diverticulum and its membranes as they expand on the bone; **(C)** Only the bony tissue with the schematic representation of the air sac diverticulum/membrane system without the surrounding flesh tissue. Same colour codes apply as described in Fig 2. Please note that the air sac is not inflated and thus intraspecific variation is highly probable.

On the dorsal and ventral sides of the cervical column, the diverticular membranes regroup and form stiffer tube-like structures that run along the entire column (Figs 3D, 4C and 4D). The dorsal intermuscular diverticular structures (IMDv) are relatively rigid, while those positioned ventrally are very flexible. The intermuscular air sac diverticula are attached to the muscle fibers originating from the vertebrae and it is difficult to find a clear separation between the bone/air sac/muscle complex. The diverticula can be seen to attach to the postzygapophyses in their posterodorsal portions via sheets of thin connective tissue, while being also attached to the *M*. *interspinalis* and *M*.*obliquospinalis*. Nerves and blood vessels are also present alongside the air sacs and their expanding diverticula. Air sac diverticular membranes from IMDv (Fig 3C) intertwine with the *M*. *obliquotransversalis* and *M*. *intertransversalis*, connect to the LVDv that, in turn, extend and cover the *facies terminalis caudalis* (Fig 4A) and *lamina arcocostalis* (Figs 3E and 4B), and extends dorsally to the *processus spinosus* (Fig 4D). The lateral vertebral diverticulum is also attached to the *M*. *intertransversalis* on the lateral side of the *processus transversus*. Posterior to the vertebra, the LVDv is anchored onto the posterolateral margin of the *foramen vertebrale* via membranous extensions. A pneumatic foramen is exposed at the end of the centrodiapophyseal lamina on the *arcus vertebrae*. The foramen is 2 mm in diameter and is invaded by the LVDv diverticulum that is attached to the *foramen vertebrale* (Figs 4A, 4B and 5). This diverticulum also attaches directly to the prezygoparapophyseal lamina.

A thinner membrane originates (AnDv) and extends from this diverticular tissue to the main vertebral body, covering the *corpus* and connects to the supramedullary diverticulum (SMDv) that covers the wall of the *foramen vertebrale*.

Another pneumatic foramen is present on the inner (medial) and posterior side of the *ansa costotransversaria*. The foramen is covered by LVDv extensions that also invade it. The foramen is large enough (10 mm) to possess well-expressed bony margins to which the diverticulum is attached via membranous extensions. The foramen extends posteromedially for 10 mm into the bone. This portion of the air sac diverticulum is connected to the *M*. *intertransversalis* that passes from the *ansa costotransversaria*. Removing the *M*. *obliquotransversalis* from the anterolateral side of the vertebra reveals the presence of intermuscular diverticular tissue that is connected to the muscle from beneath. Pneumatic foramina (1 mm in width) are also located laterally on the main vertebral body and are covered by a thin membranous film originating from nearby diverticular extensions. On the dorsal surface of the vertebrae, the supravertebral diverticula (SVDv) forms a network of thin membranes clustered within the posterior cavity of the bifurcated *fossa spinalis*. Further dissection revealed that the ventral part of the SVDv was connected to both the *M*. *obliquotransversalis* and the *M*. *longus colli ventralis*. CV5 exhibits a 2 mm wide pneumatic foramen positioned ventrolaterally on the *corpus* that is directed mediodorsally and anteriorly. The SVDv has a number of extensions there, anchored around the ventral margin of the foramen, continuing with further extensions around the margins of the foramen that also invade it. Furthermore, on CV4 and CV6, the ventral portion of the lateral vertebral diverticulum anchors and extends from the ventral side of the *facies terminalis cranialis* continuing around the anterior edges of the diapophyses and *processi costarii*. The dorsal portion of the SVDv on the *processus spinosus* is covered by the *M*. *obliquospinalis* and *M*. *interspinalis*. Further observations from CV7–15 show that the *M*. *obliquotransversalis* is covered externally by a thin air sac diverticular membrane (IMDv) that, in turn, connects that muscle with the *M*. *intertransversalis* as the membrane is uniformly distributed around the vertebra. The cervical muscles are attached to the roughened lateral surfaces of the *facies terminalis cranialis*, *facies terminalis caudalis* and diapophyses. The air sac diverticula are anchored in many of the same attachment points and cover the muscles associated with these origins. Therefore, the muscle-air sac diverticular system lies beneath the subcutaneous muscles. Inner folds of the ventral air sac system are covered by the *M*. *intertransversalis*. The ventral membrane extensions cover internally only a part of the *M*. *longus colli ventralis*. In addition, the *M*. *intertransversalis* also expands and attaches ventrally and externally onto the cervical ribs.

The membranous air sac diverticulum, an expansion of the LVDv, which is attached laterally to the *corpus* does not penetrate the neurocentral suture, but is firmly attached to it. The air sac diverticulum that extends over the lateral surface of the vertebra anchors for about 15 mm along the bone surface and extends anteriorly and medially below the *processus costarius* and posteriorly extends and occupies the *ansa costotransversaria*, forming a network of diverticula that expands within the interior of this ring and pneumatizes it. The diverticula extend dorsolaterally from a ventral direction to cover every possible area of the cervicals, thereby associating air sacs with muscles and osteological landmarks. The membranous diverticular network is apparent within the *ansa costotransversaria* and is covered by muscles in almost all directions. Connections with existing laminations cannot be observed with ease in most of the cervicals. However, all existing laminae on the ostrich cervical vertebrae serve as points of anchorage for both muscle and air sac attachments in the same places under the same pattern described above.

## Discussion

### Preliminary overview of avian pneumaticity

The results of this research demonstrate not only variable expression of pneumatic features, but also the relationships between them. Our observations of potential pneumaticity are not conclusive as there is a considerable amount of variability in the expression of pneumaticity even among ratites. It is evident that, by analogous comparison, the non-volant ratites such as rhea, ostrich, emu, cassowary, moa, and kiwi, as well as the semi-volant ratites like tinamous, have more pneumatic skeletal (vertebral and appendicular) elements than the volant ducks, the non-volant divers (penguins), and the semi-volant and semi-aquatic (loons, grebe) birds (Tables 1–5 and S1 Table and S3–S13 Tables). The observation of skeletal material sheds some light and verifies the acquisition of pneumatic foramina by avian taxa that are considered [3–6,46, 66] to be semi-pneumatic or even completely apneumatic [36] (penguins, loons). Thus, ducks, kiwis, penguins, loons, and grebes require further study (CT scans, dissections) to explore their putative pneumatic features. Having taken general measurements (i.e., total height and total length) from all examined avian specimens (see S2 Table), we find an increase in both the complexity and occurrence of pneumaticity in ratites as body size increases, an observation that has also been documented in non-avian theropods [41]. While this study does not address ratios of body size and Pneumaticity Index among the examined taxa, it does provide a clear and simple representation of the pneumatic data that can be used as a baseline for future quantitative studies on vertebral pneumatization (see Tables 2–5 and S1–S13 Tables).

### Association of vertebral pneumatic features with air sac diverticula

Vertebral laminae may act as stress/strain absorbers [53,67] and do function as points of attachment for muscles and other tissues [18,37,38,54,67]. Our observations on ostrich necks indicate that the vertebral laminae are more extensively expressed when adjacent to a pneumatic foramen, suggesting that laminae also act as important anchorage points for the air sac diverticula to attach to the bone before they invade it. The length of the laminae varies according to the size of the vertebra. It is possible that the laminae serve as structural supports for the vertebra, compensating for the loss of cortical and cancellous bone due to the formation of foramina and fossae (e.g., [18,28,38]).

Our observations on the dissected ostrich neck and the avian osteological specimens indicate diverse expressions of pneumatization and reveal a set of complex associations between air sac diverticula, osteological pneumatic traits, and muscles. The air sac diverticular membranes are often attached to the lateral surface of the centrum, sometimes directly on the neurocentral suture (the individual was subadult), and, as aforementioned, in many cases, they are attached to the laminae. Penetration was not clearly visible, but the membranes firmly adhered to the suture. This observation supports those of previous studies (e.g., [9,13,27,28,33]) that, on a microscopic level, the cells of the air sac diverticula 'push through' the osteocytes during post-hatching ontogeny, invading and aerating the bone internally. Furthermore, the motor nerves were intertwined with the muscles that were, in turn, associated with the air sac membranes. This may indicate that muscle fibres responsible for neck flexion and constriction contribute to the expansion and compression of any associated air sac diverticula. If this is true, then the expansion and compression of the pneumatic diverticula would not only be mediated centrally through general pulmonary ventilatory activity, but also by local mechanical influences from the associated musculature. However, this concept has not been tested experimentally, nor has the significance of such potential functions been addressed.

Osseous rugosities (Fig 1D) on the arcocostal surface in the large ratites provide attachments for the intertransverse and obliquotransverse muscles along the vertebrae, and serve as attachment points for the air sac diverticula (e.g., LVDv, SVDv) and their membranous extensions. A point of clarification here is that, in general, the air sac diverticula were quite flexible but we observed that the diverticular extensions and membranes that expand from the main diverticula and become attached to the osseous rugosities and laminae become more rigid before invading the bone via foramina. The rough-textured protrusions lie between the centrodiapophyseal and arcocostal laminae and often bear pneumatic foramina, thus verifying during the ostrich dissection that both the muscles and membranous air sac diverticula utilize the same anchorage points. Based on our observations of the locations of pneumatic foramina on the cervical vertebrae, we verify that the air sac diverticula expand on the bone's surface in nearly every direction and penetrate the bone by attaching themselves to the various laminae and fossae/foramina described in this study, corroborating previous work by O'Connor [28,36]. In addition, the camellate network-like structures in the entrance of the transverse foramina, the spinal posterior fossa, and the inner wall of the costotransverse ring, indicate that the thin septa (and the septated foramina) serve as firm points of adhesion for the diverticula before they invade the bone. This leads to the logical conclusion that, during the early stages of avian ontogeny, several weeks after hatching [34,35], the air sacs expand their membranous extensions forming the diverticula, which, in turn, seek stable anchorage on the bone's laminae. For example, ostrich and emu cervical vertebrae often bear more than one arcocostal lamina. They can be described as laterally expressed, longitudinal ridge formations, often having a pneumatic foramen between or on them. Such a feature was expressed to a limited extent, when present, in smaller ratites (i.e., kiwi and tinamou) and was absent in non-ratites (duck, loon, grebe, and penguin). This evidence supports weakly a possible relationship between body size and the degree of PSP.

As noted earlier, pneumatic foramina, with and without septations, were present on the vertebrae and/or appendicular elements of birds with reduced pneumaticity, such as kiwi, penguin, grebe, and loon (Tables 1–5, S1 and S3–S13 Tables). Kiwis are ratites that are strictly non-diving and obligatory cursorial birds, as they have entirely lost the ability to fly. Penguins are marine foragers that need to be able to dive deep without the buoyancy constraints imposed by water. According to Meister's study [66], the long bones of penguins (mainly femora) are stiff and very dense, but not wider in diameter than in other birds of similar size. They have enormously thick compacta and correspondingly limited marrow volume. Their long bones have a thick periosteum, which is strongly adhered to the inner cancellous layers of the bone. Habib [68] also notes that the thick cortical bone of penguins serves both for ballast and strength under extreme loading conditions such as manoeuvring in deep water. Both Meister and Habib [66,68] have observed that these characteristics are not present in volant birds. Therefore, penguins require robust bones to circumvent buoyancy issues. Perhaps then, the presence of pneumatic features in penguins and other limitedly pneumatic taxa might only be a superficial trait without any intraosseous aeration occurring in their limb bones and vertebrae, or it could be a shared primitive trait retained from a flying ancestor.

### Phylogenetic interpretations and implications for pneumaticity in extinct taxa

**Fig 6:**
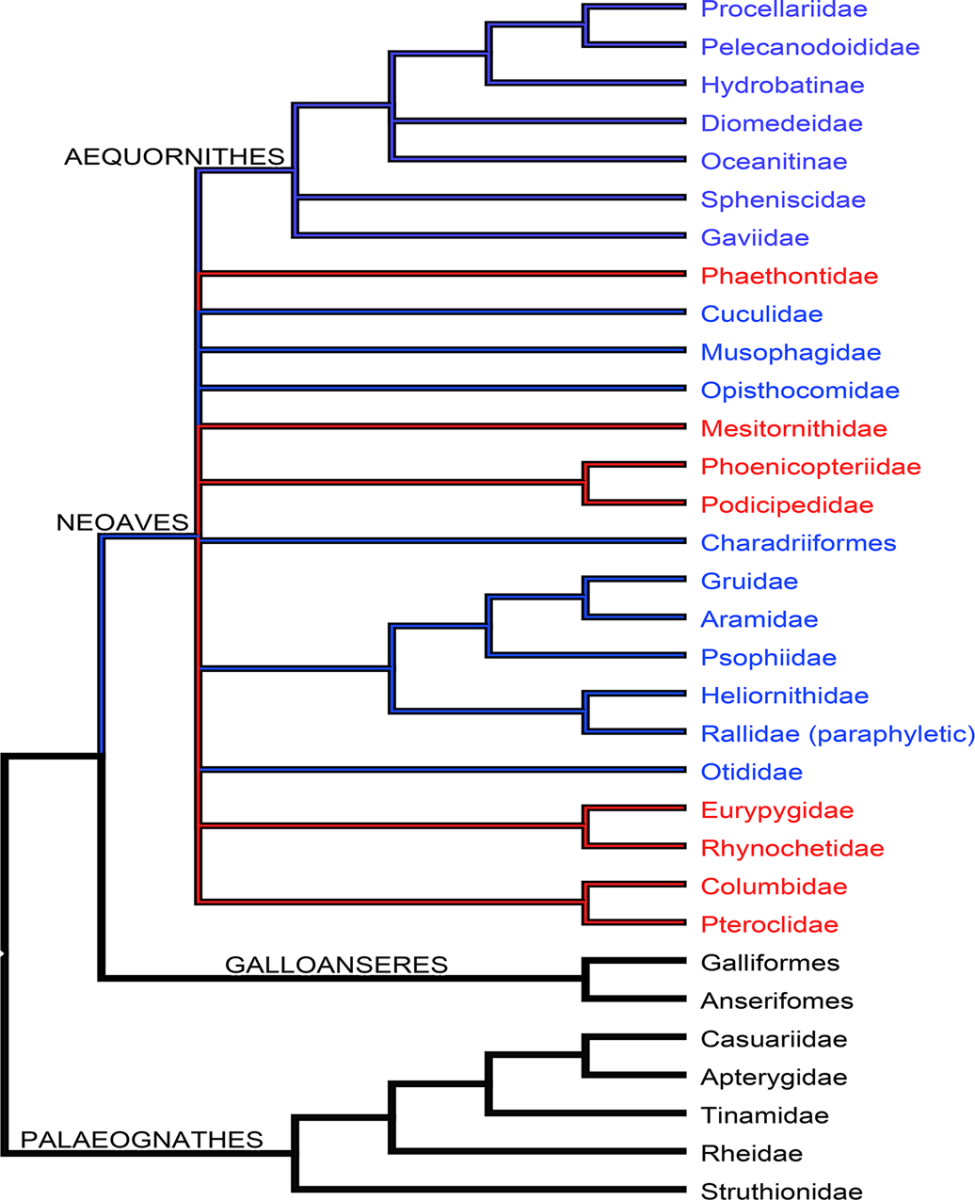
Phylogenetic interrelationships of the avian taxa in this study. Part of a consensus phylogenetic tree [69] based on combined genetic and morphological data showing the interrelationships of the avian taxa included in this study. All clades and corresponding taxa except those in black (Galloanseres and Palaeognathes) are within Neoaves. Those labelled red are Metaves within the Neoaves.

All ratites exhibited extensive PSP, including not only the vertebral column, ribs, and girdle elements, but also the humeri, femora, and even, possibly, distal appendicular elements in some specimens (see S1 Table), but the latter rarely occurs [6,36]. A recent phylogenetic analysis [69] of bird interrelationships recovered a consensus tree based on both morphological and molecular data (Fig 6). Within Palaeognathae, Casuariidae and Apterygidae are sister clades and closer to each other than they are to Tinamidae, Rheidae, and Struthionidae. The earliest neornithine divergence, that of the palaeognaths, is among the best-resolved phylogenetic relationships within the avian tree and the Galloanserae (Anseriformes+Galliformes) is considered to be the successive sister group to all other Neoaves [69]. It may not stand as compelling morphological evidence to support a close phylogenetic relatedness between Anseriformes and Palaeognathes but it is worthy to note, based on our observations on limited avian skeletal material, that the ducks share more pneumatic elements with the ratites than with the other Neoaves examined. For example, ducks and most ratites share laminated foramina, laminated fossae and foramina within fossae in their cervical vertebrae while these features are absent in the loons, the grebe and the penguins (Tables 2–5 and for more details see S3–S13 Tables). In Neognathae, Podicipedidae (grebes) are sister clade to Phoenicopteriidae (flamingos) and not as closely related as previously thought to Gaviiformes (loons) and Sphenisciformes (penguins) [70,71]. Tinamous, kiwis, and moas have high PI% (Table 1 and S1 Table) and symplesiomorphically share pneumatic elements in their cervical vertebrae, few pneumatic traits in their thoracic vertebrae, and even fewer in their synsacrals. Consequently, it is predicted that the last common ancestor of these three taxa had foramina, fossae, and laminae on its cervical vertebrae. Moreover, *Struthio*, *Rhea*, *Dromaius*, *and Emeus* (moa) share most, if not all, pneumatic traits in their cervical, thoracic, and synsacral vertebrae. The sister clades *Struthio* and *Rhea* share the same pneumatic traits between them, as do *Dromaius* and *Casuarius*. Thus, the distribution of the pneumaticity data in the ratites is in agreement with their phylogenetic affinities (Fig 6). Therefore, patterns of pneumaticity may have a strong phylogenetic signal in ratites.

Anseriformes, Gaviiformes, Podicipediformes, Sphenisciformes, Tinamiformes, and Apterygiformes occupy different environmental niches and exhibit different modes of life, with the exception of loons and grebes that live in similar environments and adopt the same foraging strategies [29]. Their pneumatic characteristics must have been inherited from a distant common ancestor before the split between Palaeognathae and Neognathae. If the foramina, fossae, and laminae serve as features for weight reduction in aerial locomotion, what could the purpose be of retaining the pneumatic features after losing the ability to fly [72] in the strictly cursorial ratites? The most plausible explanation could be that they simply retained these inherited plesiomorphic pneumatic characteristics, although a reduction in limb bone mass may have been advantageous during the evolution of cursorial locomotion in some taxa (see also [41]).

Theropod lineages with large body sizes have been shown to exhibit increased PSP [41], suggesting that mass reduction might have been one of the factors that has affected the early stages of evolution of pneumaticity. However, the body size limit for extensive pneumatization in theropods was lower, especially to those clades that were more closely related to birds (maniraptorans). Thus, a limited association between body size and PSP appeared before the avian origins and should not be accepted as a prerequisite to an adaptation for flight [41]. Benson et al. [41] proposed that changes in osteological density of “small, non-volant maniraptorans resulted in energetic savings as part of a multi-system response to increased metabolic demands”([41]:1). Acquisition of extensive PSP in small-bodied maniraptorans may have indicated avian-like endothermy but considering that some fish have PSP [60,61] emerging from gas bladder diverticula, and that some varanids and chameleons have pneumatic diverticula that do not invade their bony tissues, the correlation between endothermy and the presence of either PSP or pulmonary diverticula is ambiguous, not least because it is uncertain when endothermy appeared in theropods/birds. The findings of Benson et al. [41] confirm previous research (e.g., [30,47]) that demonstrated the existence of extensive pneumaticity in abelisaurids and allosaurids, theropods that could reach moderately large sizes within a short period. The presence of extensive vertebral pneumatization and lamination in sauropods [18,37,39] further supports arguments about adaptation for mass-reduction, since overall vertebral volume could be comprised 60% by air [37], as well as for reducing the need for structural support during the evolution of their long necks [67,73].

Moreover, in extant volant birds, the presence of a respiratory system composed of non-compliant gas exchanging lungs and compliant air sacs [12,73] enables a higher oxygen uptake under resting conditions than seen in similarly-sized mammals [74], thus facilitating birds in their evolutionary steps of achieving high-altitude flight. We hypothesize that vertebral pneumatization, as a result of penetrating diverticula from the gas exchanging lung-air sac system, serves the purpose of lightening the skeleton by reducing the cortex thickness [75] and bone density [28] since marrow is replaced by air, thus aiding flying birds to achieve high altitude flight [36].

Having observed vertebral laminae and their close association with other pneumatic features (e.g., pneumatic foramina, fossae) and cervical air sac diverticula we argue that the laminae, although they may represent only ambiguous evidence for the presence of a heterogeneous respiratory system, serve a combined function in many cases, acting as the attachment points for both muscles and pneumatic diverticula, thus agreeing with observations made by previous studies (e.g., [4,13,14]). A final observation of ours that also agrees with previous work on birds (e.g., [4,28]) is that the air sac diverticula become less compliant and almost rigid before they invade the bone via pneumatic foramina (Fig 4A and 4B).

Wedel [38] has argued that “Two problems with the identification of laminae that are relevant to the question of pneumaticity are how well developed a ridge of bone must be before we call it a lamina, and whether laminae are primarily additive structures formed by the deposition of new bone, or are simply bone that is left over following the formation of fossae” ([38]: 210). We propose that the principal function of vertebral laminae may have been either to partition pneumatic diverticula on the neural arch and centrum, or to act as insertions for the neck and trunk musculature. Moreover, the increase in laminar complexity and the appearance of four novel laminae early in sauropod evolution implies an important structural role [37]. Furthermore, laminae have also been found on the vertebrae of salamanders, like the plethodontid *Aneides lugubris* where plates of bone on its dorsal vertebrae connect the centrum with the parapophyses [76]: although these laminae are not homologous to those found in archosaurs, the existence of such similar osteological features indicates the structural importance of extensive vertebral ossification in tetrapods [38]. The arrangement of vertebral laminae on the neural arch may reflect muscular, tendinous, or ligamentous stresses during development, as is often the case with trabeculae in the bone of living animals [4,27,28]. Evidently, bone aeration can be facilitated by minimizing the development of the cortical bone during ontogeny via diverticular expansion (which leads to creation of broad fossae and light bones) (e.g., [73,52]) and/or by penetrating the cortical tissue via diverticula, thus forming foramina on the exterior surface and trabeculae within the cancellous bone tissue (e.g., [39,73,38]).

## Conclusions

a) Fossae, foramina, and laminae are present within the vertebral column either as single structures or as parts of character combinations, leading to the recognition of seven pneumatic categories (Tables 2–5, and S3–S13 Tables). It is important to note that the osseous septa connecting the clustered foramina, wherever present, are associated with anchoring the diverticula before they invade the bone via foramina.
b) The distribution of pneumatic characters and the associations among them and with the air sac system are taxonomically and individually variable. Avian taxa previously considered to be postcranially apneumatic show minimal or moderate expression of potentially pneumatic features, which was probably inherited from a pneumatic common ancestor. Nevertheless, appropriate tests of soft and hard tissue relationships in avian specimens with minimal or absent pneumaticity are essential to confirm these suggestions.
c) Ratites display higher degrees of PSP than the neognaths examined (Tables 1–5 and S1–S13 Tables).
d) The presence of laminae should not be used as evidence for the existence of PSP unless careful examination of the vertebrae shows the presence of other established pneumatic features (foramina, fossae). Although laminae, foramina, and fossae imply the presence of air sacs, the reverse (i.e., the presence of air sacs does not necessitate the formation of PSP) is not equally likely, especially in diving birds. Therefore, the presence of laminae does not necessitate the presence of PSP, since laminae are not always associated with foramina or fossae.
f) Membranous air sac extensions and their associated muscles share the same attachment points. Air sac diverticula attach to laminae or foramen/fossa margins before invading the bone (Figs 2–5).
g) Among extant taxa, the pulmonary-air sac system is unique to Aves (e.g., [3,18]) but its expression in the form of PSP is absent or only minimally present in some avian clades (e.g., penguins, loons, and grebes). Thus, we can be confident of the association between PSP and pneumatic diverticula, although the purpose of the presence of PSP with relation to the respiratory function is still unknown.

## Supporting Information

S1 Fig Left lateral view of ostrich air sacs. Left lateral view of the air sacs (filled with latex) of a 3-month-old ostrich after removal of the pectoral limb, lateral body wall and laterally positioned thigh muscles. The femur has also been opened longitudinally to expose the air sac housed within the bone. 1, lung; 2, cervical air sac; 3, lateral component of clavicular air sac; 4, cranial thoracic air sac; 5, caudal thoracic air sac; 6, femoral diverticulum of abdominal air sac; 7, perirenal diverticulum of abdominal air sac; 8, abdominal air sac; 9, gastric diverticulum of clavicular air sac. A.J. Bezuidenhout, H.B. Groenewald and J.T. Soley, personal observations, 1998 [15].
(DOCX)

S2 Fig Ostrich. *Struthio camelus* (BRSMG Af962).
(DOCX)

S3 Fig Moa. *Emeus crassus* (BRSMG Cg976).
(DOCX)

S4 Fig Emu. *Dromaius novaehollandiae* (BRSMG Ab4163).
(DOCX)

S5 Fig Cassowary. *Casuarius galeatus* (BRSMG Af963).
(DOCX)

S6 Fig Rhea. *Rhea americana* (NHMUK 2.5.1).
(DOCX)

S7 Fig Tinamous. (a) *Crypturellus obsoletus* (NHMUK S/1972.1.23); (b) *Crypturellus undulatus* (NHMUK S/1972.2.6.7); (c) *Eudromia elegans* (NHMUK S/1972.1496); (d) *Nothura maculosa* (NHMUK S/1972.3.24.5); (e) *Rhynchotus rufescens* (*NHMUK* S/1972.2.16.62).
(DOCX)

S8 Fig Kiwis. (a) *Apteryx australis haasti* (NHMUK 1456); (b) *Apteryx australis lawri* (NHMUK 1488); (c) *Apteryx oweni* (NHMUK 1458).
(DOCX)

S9 Fig Ducks. (a) *Anas* sp. indet. (BRSUV unregistered); (b) *Melanitta* sp. indet. (BRSMG Af974).
(DOCX)

S10 Fig Penguins. (a) *Pygoscelis papua* (NHMUK unregistered); (b) *Pygoscelis antarcticus* (BRSUV unregistered).
(DOCX)

S11 Fig Loons. (a) *Gavia adamsii* (NHMUK S/1996.68.1); (b) *Gavia stellata* (NHMUK S/1985.18.1).
(DOCX)

S12 Fig Grebe. *Podiceps major* (NHMUK S/1952.1.47).
(DOCX)

S1 File Air sac system in ostrich. (DOCX)

S1 Table Pneumaticity states of appendicular elements of the 11 avian taxa based on the presence (+)/absence (-) of pneumatic foramina. PI%* is derived as the number of pneumatic elements (i.e. vertebral and appendicular elements with pneumatic foramina) over the number of total elements (after [4]). Wherever elements come in pairs in an animal, pneumatization stands present for the whole element even if it is present in one of the two elements in that pair. Abbreviations: HM, humerus; FM, femur; SC, scapula; CC, coracoid; ST, sternum; RB, ribs; FC, furcula; TT, tibiotarsus; TM, tarsometatarsus.
(DOCX)

S2 Table Size measurements (total height and total length) obtained from the 11 avian taxa. The measurements are in centimeters (cm). For the mounted specimens, total height corresponds to the straight distance from top of the skull to the ground and total length is the straight distance from anterior (i.e. tip of the beak) to posterior (i.e. the end of the last caudal/pygostyle) of the animal. In loose specimens (i.e. disarticulated/not mounted) the measured length is an approximation. Ostrich* refers only to the BRSMG mounted specimen.
(DOCX)

S3 Table Proportion of vertebrae exhibiting pneumatic features in ostrich (*Struthio camelus—*BRSMG Af962). (DOCX)

S4 Table Proportion of vertebrae exhibiting pneumatic features in moa (*Emeus crassus—*BRSMG Cg 976). (DOCX)

S5 Table Proportion of vertebrae exhibiting pneumatic features in emu (*Dromaius novaehollandiae*—BRSMG Ab4163). (DOCX)

S6 Table Proportion of vertebrae exhibiting pneumatic features in cassowary (*Casuarius galeatus—*BRSMG Af963). (DOCX)

S7 Table Proportion of vertebrae exhibiting pneumatic features in rhea (*Rhea americana—*NHMUK 2.5.1). (DOCX)

S8 Table Proportion of vertebrae exhibiting pneumatic features in kiwis (*Apteryx australis haasti—*NHMUK 1456, *Apteryx australis lawri—*NHMUK 1488, *Apteryx oweni*—NHMUK 1458). (DOCX)

S9 Table Proportion of vertebrae exhibiting pneumatic features in tinamous (*Crypturellus obsoletus—*NHMUK S/1972.1.23; *Crypturellus undulatus—*NHMUK S/1972.2.6.7; *Eudromia elegans—*NHMUK S/1972.1496; *Nothura maculosa—*NHMUK S/1972.3.24.5; *Rhynchotus rufescens—*NHMUK S/1972.2.16.62). (DOCX)

S10 Table Proportion of vertebrae exhibiting pneumatic features in penguins (*Pygoscelis papua*–NHMUK; *Pygoscelis antarcticus*–BRSUV only has laminae throughout its cervical vertebrae). (DOCX)

S11 Table Proportion of vertebrae exhibiting pneumatic features in ducks (*Anas* sp indet.–BRSUV; *Melanitta* sp. indet.—BRSMG Af974). (DOCX)

S12 Table Proportion of vertebrae exhibiting pneumatic features in loons (*Gavia adamsii—*NHMUK S/1996.68.1; *Gavia stellata—*S/1985.18.1). (DOCX)

S13 Table Proportion of vertebrae exhibiting pneumatic features in grebe (*Podiceps major—*NHMUK S/1952.1.47). (DOCX)

S14 Table List of lamina abbreviations used in the main text (following [55], [56]). (DOCX)
