# Supplementary material for: Osteological and Soft-Tissue Evidence for Pneumatization in the Cervical Column of the Ostrich (Struthio camelus) and Observations on the Vertebral Columns of Non-Volant, Semi-Volant and Semi-Aquatic Birds
Source: PLoS One. 2015 Dec 9;10(12):e0143834. doi: 10.1371/journal.pone.0143834 (PMC4674062; doi:10.1371/journal.pone.0143834)
Supplement: S1 Fig — Left lateral view of the air sacs (filled with latex) of a 3-month-old ostrich after removal of the pectoral limb, lateral body wall and laterally positioned thigh muscles. The femur has also been opened longitudinally to expose the air sac housed within the bone. 1, lung; 2, cervical air sac; 3, lateral component of clavicular air sac; 4, cranial thoracic air sac; 5, caudal thoracic air sac; 6, femoral diverticulum of abdominal air sac; 7, perirenal diverticulum of abdominal air sac; 8, abdominal air sac; 9, gastric diverticulum of clavicular air sac. A.J. Bezuidenhout, H.B. Groenewald and J.T. Soley, personal observations, 1998 [15]. (DOCX) [file pone.0143834.s001.docx]

**S1 Fig. Left lateral view of ostrich air sacs.**

**
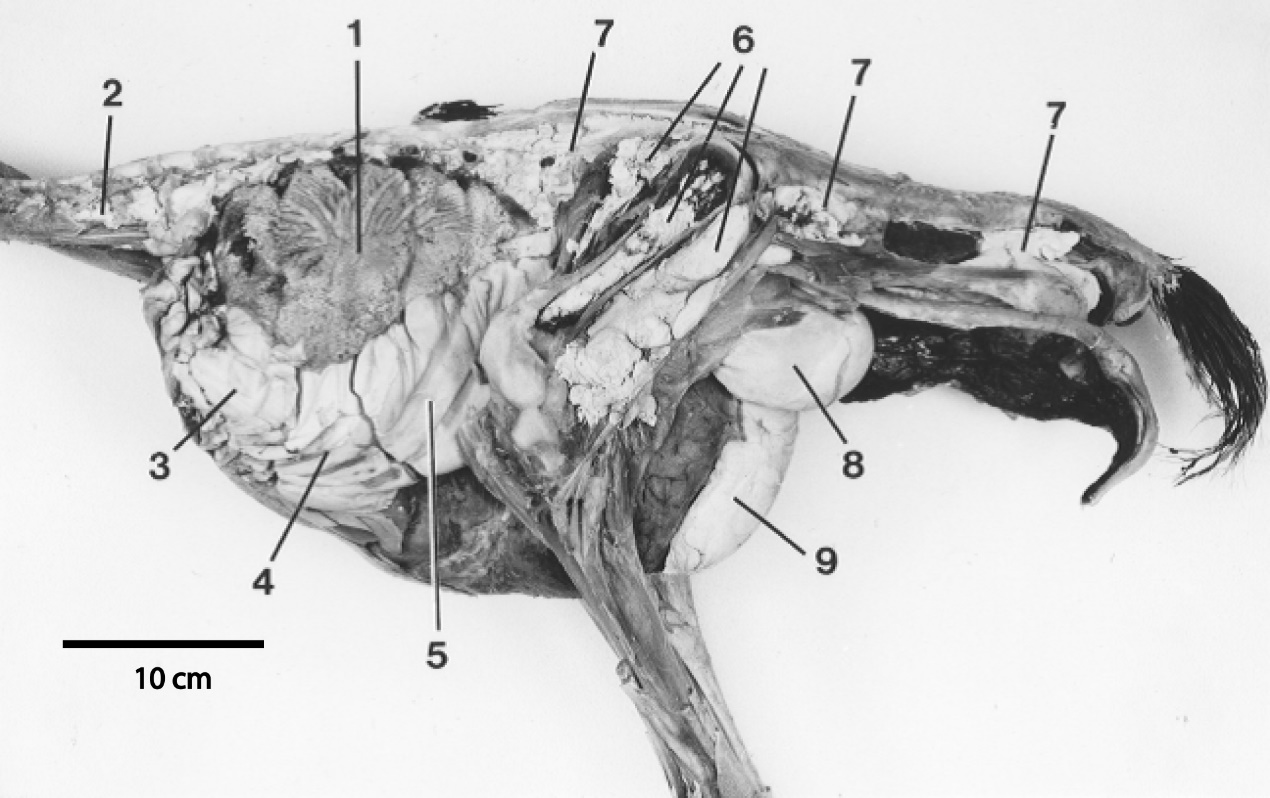
**

Left lateral view of the air sacs (filled with latex) of a 3-month-old ostrich after removal of the pectoral limb, lateral body wall and laterally positioned thigh muscles. The femur has also been opened longitudinally to expose the air sac housed within the bone. 1, lung; 2, cervical air sac; 3, lateral component of clavicular air sac; 4, cranial thoracic air sac; 5, caudal thoracic air sac; 6, femoral diverticulum of abdominal air sac; 7, perirenal diverticulum of abdominal air sac; 8, abdominal air sac; 9, gastric diverticulum of clavicular air sac. A.J. Bezuidenhout, H.B. Groenewald and J.T. Soley, personal observations, 1998 [15].
