# Supplementary figures and images for: Osteological and Soft-Tissue Evidence for Pneumatization in the Cervical Column of the Ostrich (Struthio camelus) and Observations on the Vertebral Columns of Non-Volant, Semi-Volant and Semi-Aquatic Birds
Source: PLoS One. 2015 Dec 9;10(12):e0143834. doi: 10.1371/journal.pone.0143834 (PMC4674062; doi:10.1371/journal.pone.0143834)

**Supporting Information**

**S2 Fig. Ostrich**. *Struthio camelus* (BRSMG Af962).


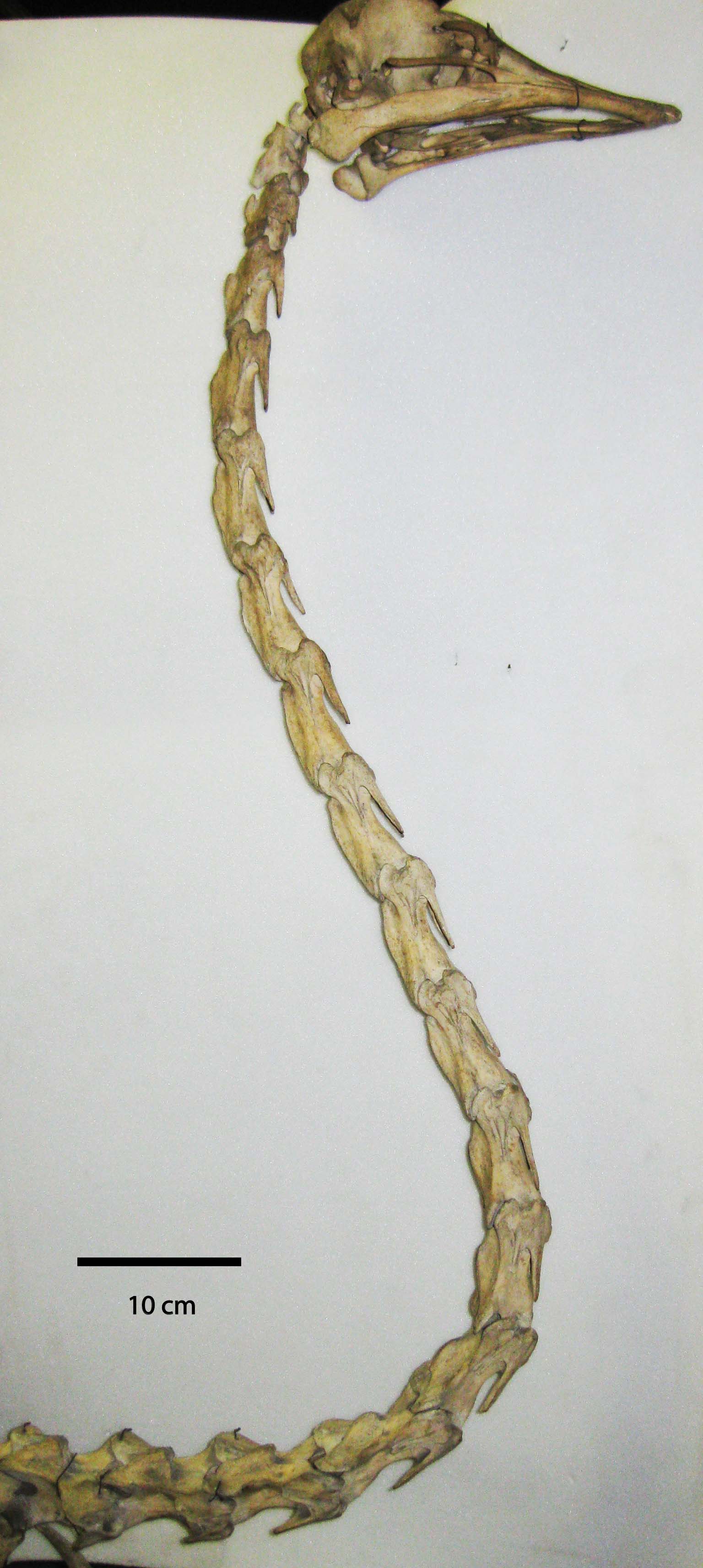

Supplement: S2 Fig — Struthio camelus (BRSMG Af962). (DOCX) [file pone.0143834.s002.docx]

**Supporting Information**

**S3 Fig. Moa**. *Emeus crassus* (BRSMG Cg976).


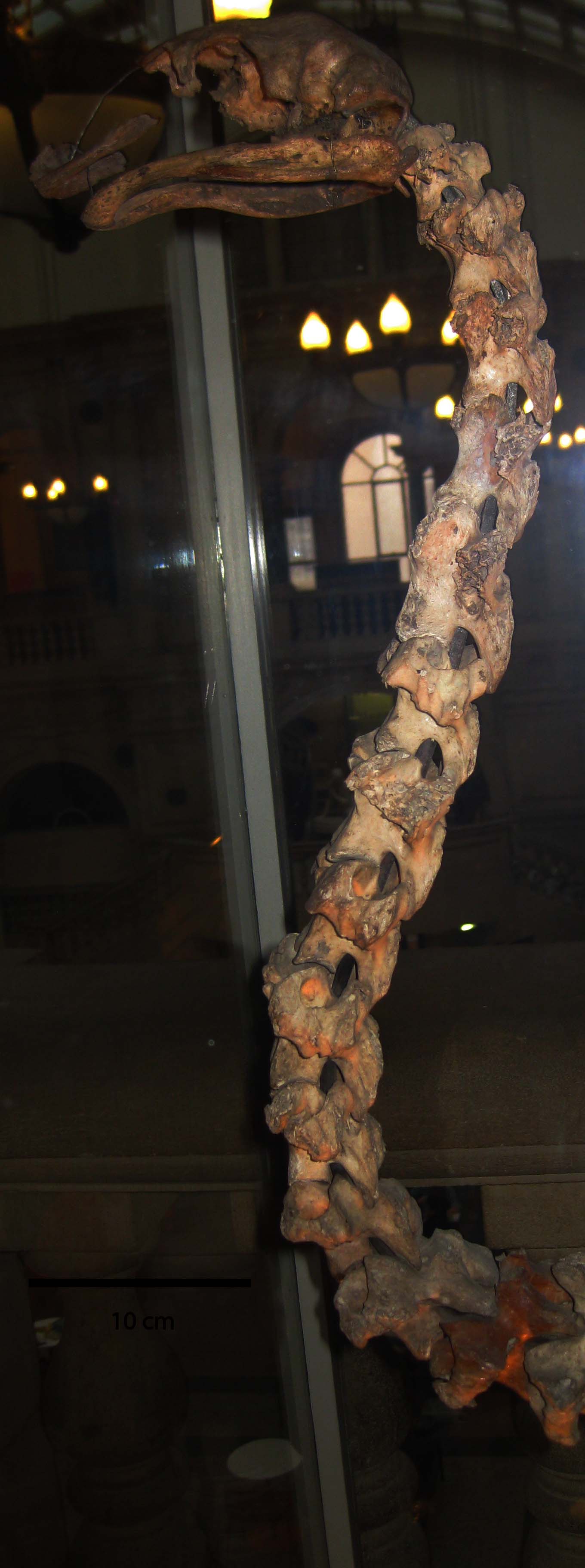

Supplement: S3 Fig — Emeus crassus (BRSMG Cg976). (DOCX) [file pone.0143834.s003.docx]

**Supporting Information**

**S4 Fig. Emu.** Dromaius novaehollandiae (BRSMG Ab4163).


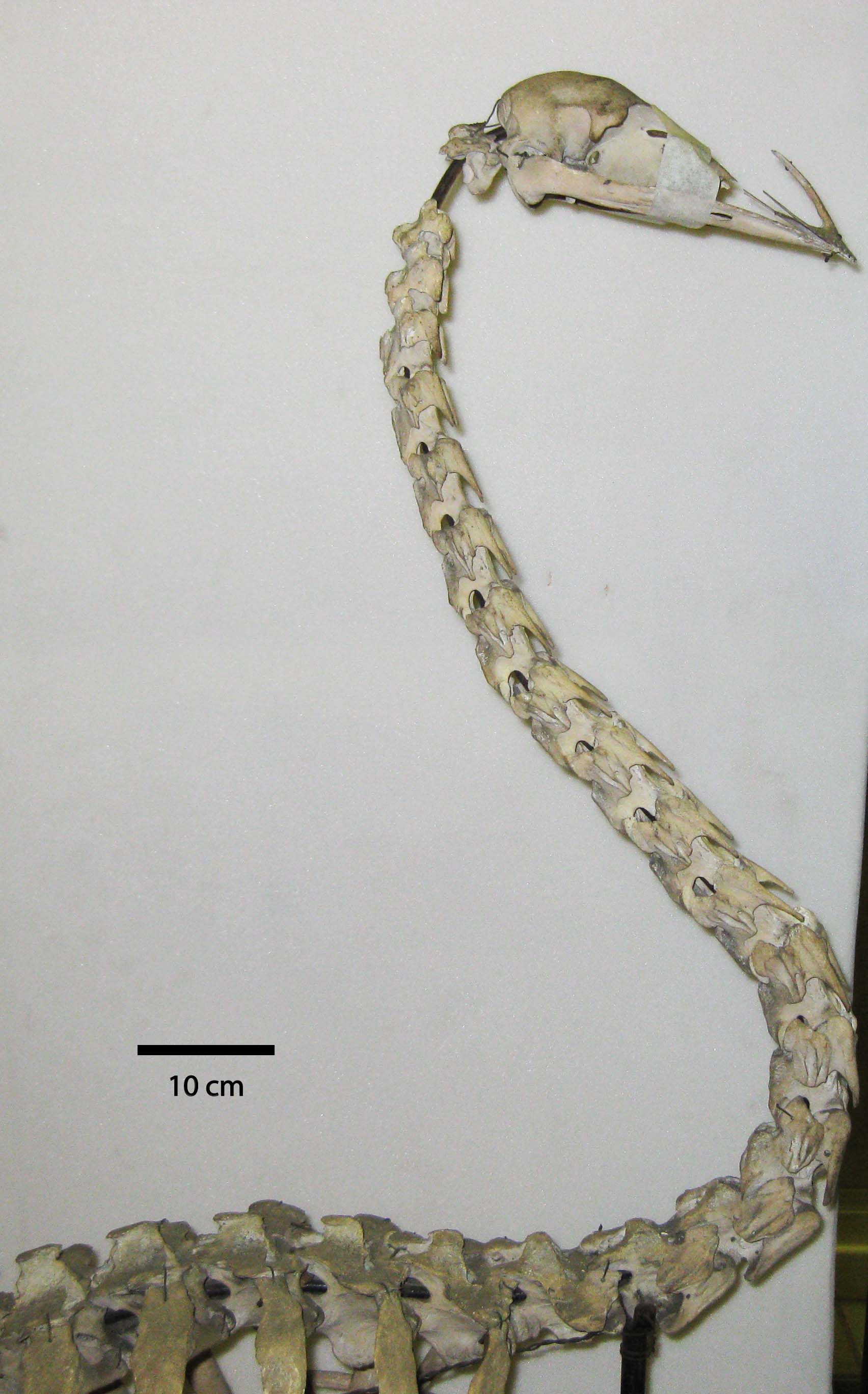

Supplement: S4 Fig — Dromaius novaehollandiae (BRSMG Ab4163). (DOCX) [file pone.0143834.s004.docx]

**Supporting Information**

**S5 Fig. Cassowary.** *Casuarius galeatus* (BRSMG Af963).


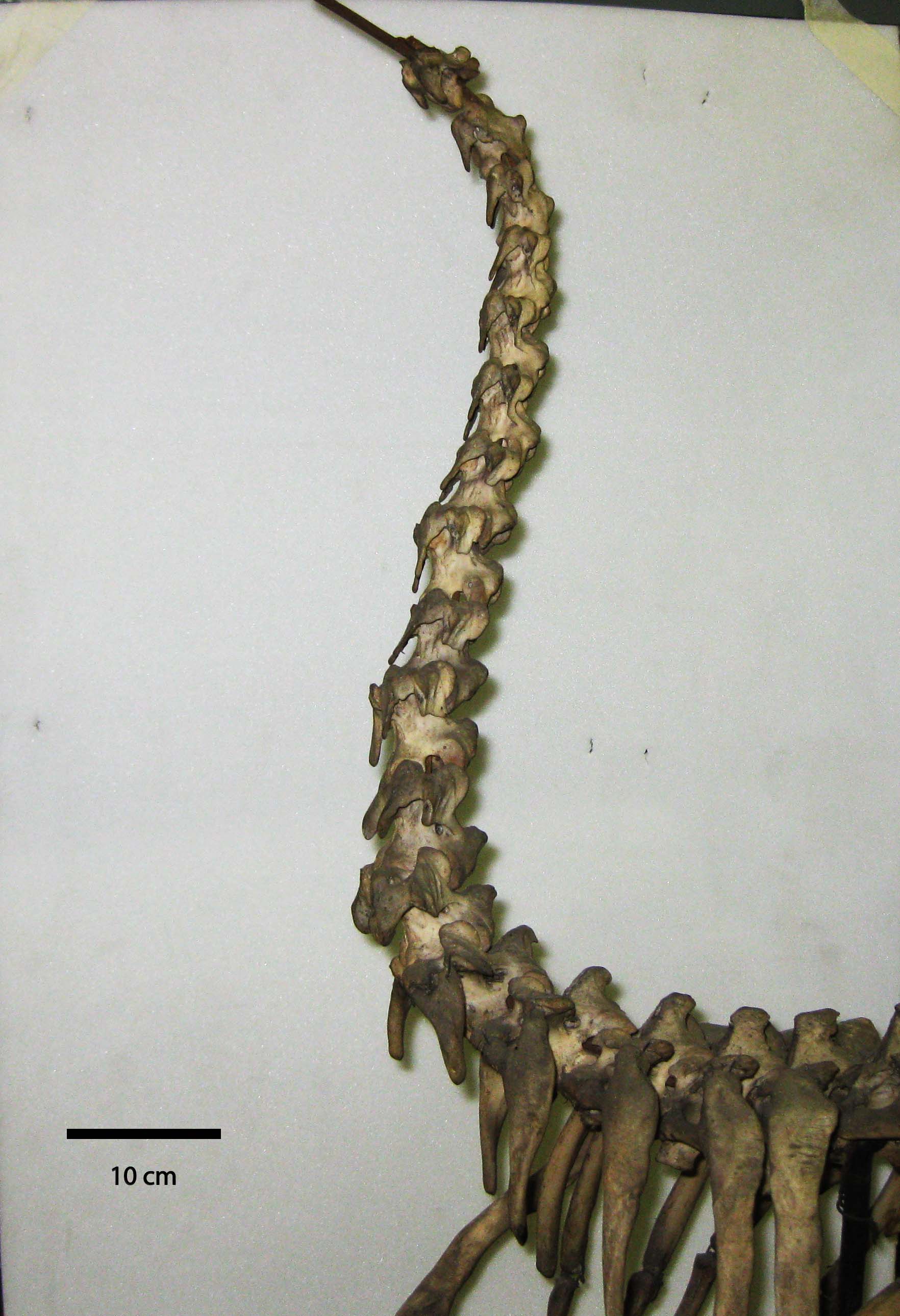

Supplement: S5 Fig — Casuarius galeatus (BRSMG Af963). (DOCX) [file pone.0143834.s005.docx]

**Supporting Information**

**S6 Fig.** **Rhea**. *Rhea americana* (NHMUK 2.5.1).


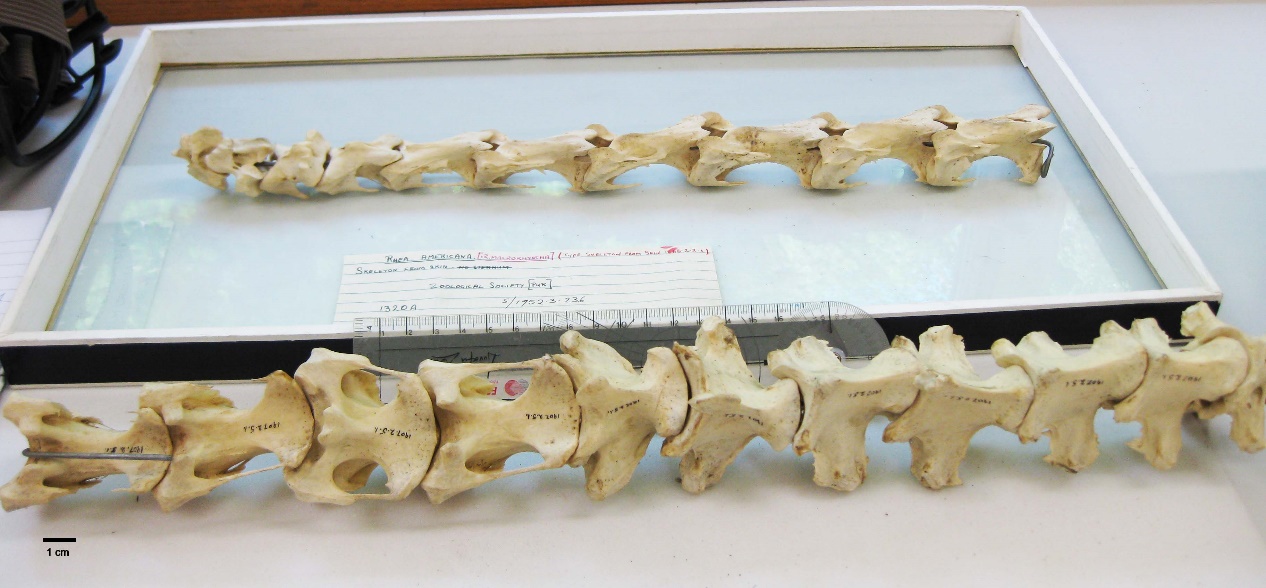

Supplement: S6 Fig — Rhea americana (NHMUK 2.5.1). (DOCX) [file pone.0143834.s006.docx]

**Supporting Information**

**S11 Fig. Loons.** (a) *Gavia adamsii* (NHMUK S/1996.68.1); (b) *Gavia stellata* (NHMUK S/1985.18.1).

(a)

**
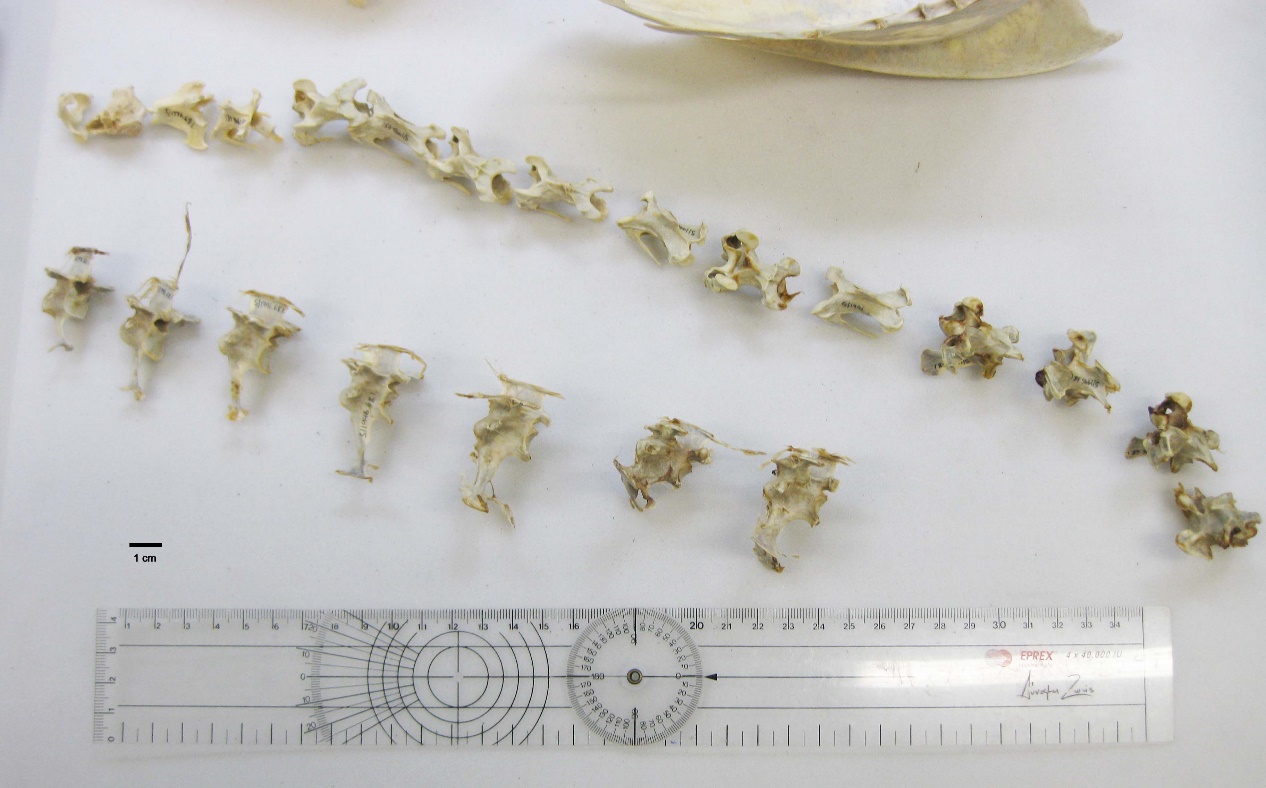
**

(b)


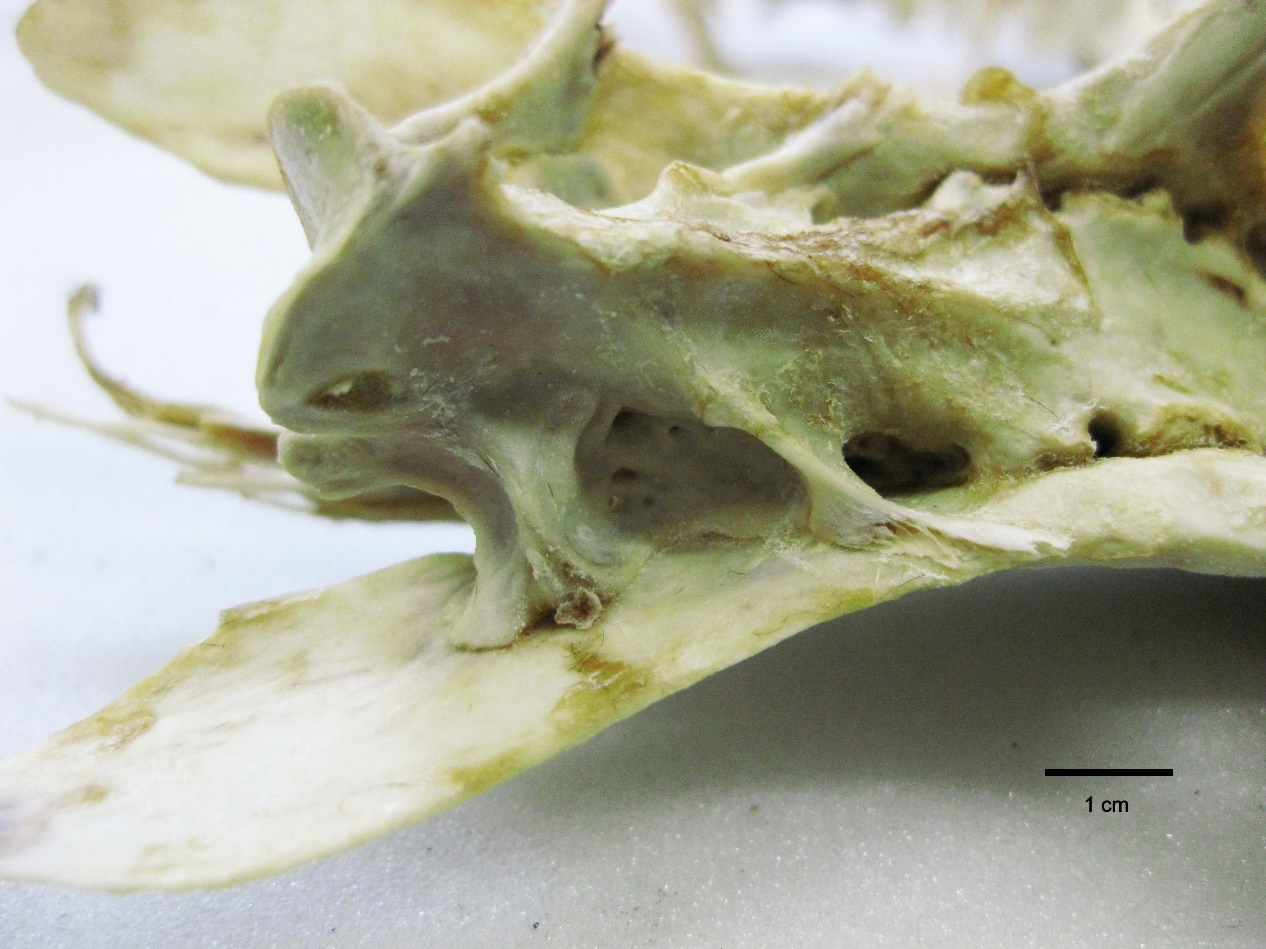

Supplement: S11 Fig — (a) Gavia adamsii (NHMUK S/1996.68.1); (b) Gavia stellata (NHMUK S/1985.18.1). (DOCX) [file pone.0143834.s011.docx]

**Supporting Information**

**S12 Fig. Grebe.** *Podiceps major* (NHMUK S/1952.1.47).


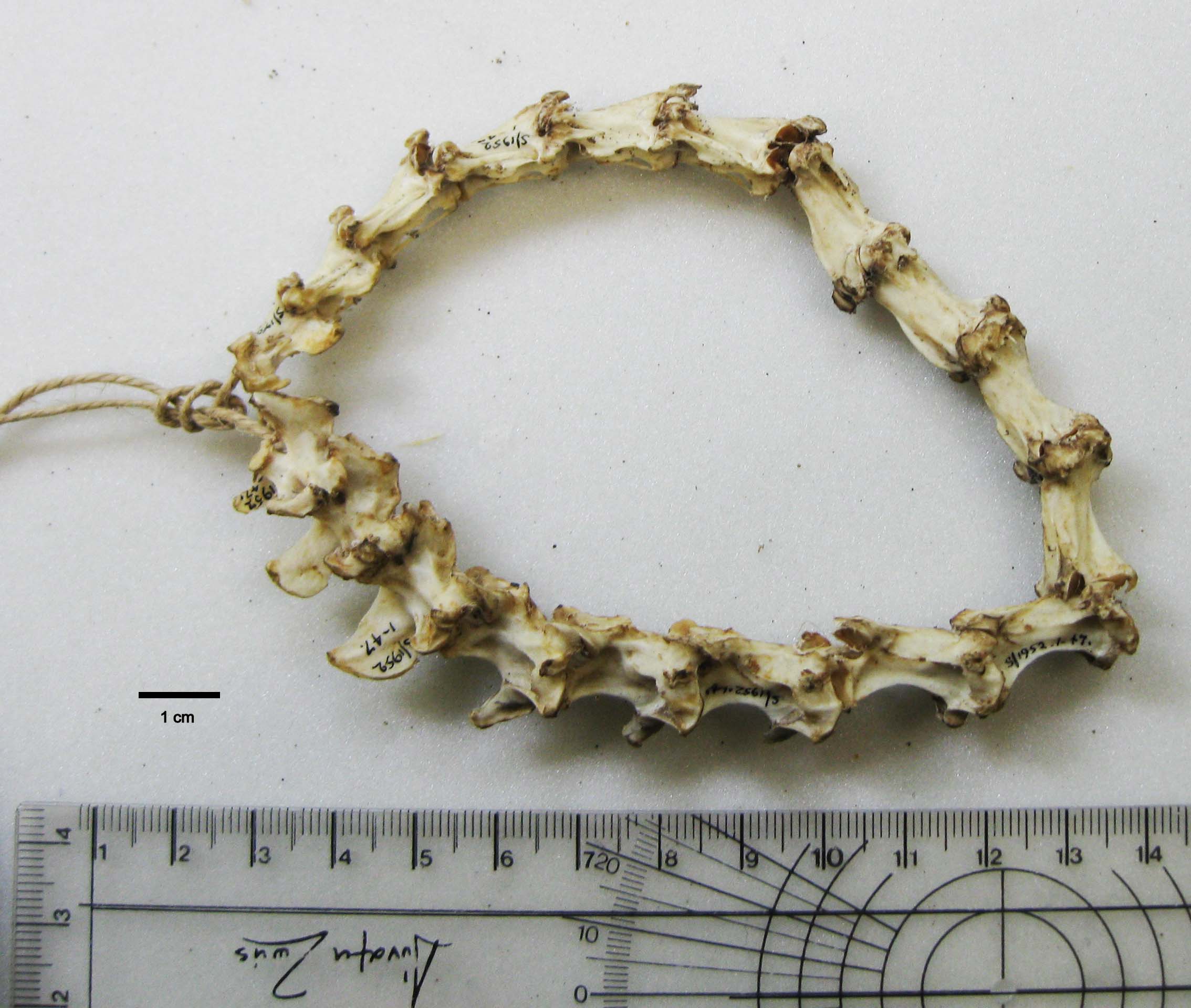

Supplement: S12 Fig — Podiceps major (NHMUK S/1952.1.47). (DOCX) [file pone.0143834.s012.docx]
