# Supplementary material for: Osteological and Soft-Tissue Evidence for Pneumatization in the Cervical Column of the Ostrich (Struthio camelus) and Observations on the Vertebral Columns of Non-Volant, Semi-Volant and Semi-Aquatic Birds
Source: PLoS One. 2015 Dec 9;10(12):e0143834. doi: 10.1371/journal.pone.0143834 (PMC4674062; doi:10.1371/journal.pone.0143834)

**Supporting Information**

**S7 Fig. Tinamous.** (a) *Crypturellus obsoletus* (NHMUK S/1972.1.23)*;* (b) *Crypturellus undulatus* (NHMUK S/1972.2.6.7)*;* (c) *Eudromia elegans* (NHMUK S/1972.1496)*;* (d) *Nothura maculosa* (NHMUK S/1972.3.24.5)*;* (e) *Rhynchotus rufescens* (*NHMUK* S/1972.2.16.62).

(a)

**
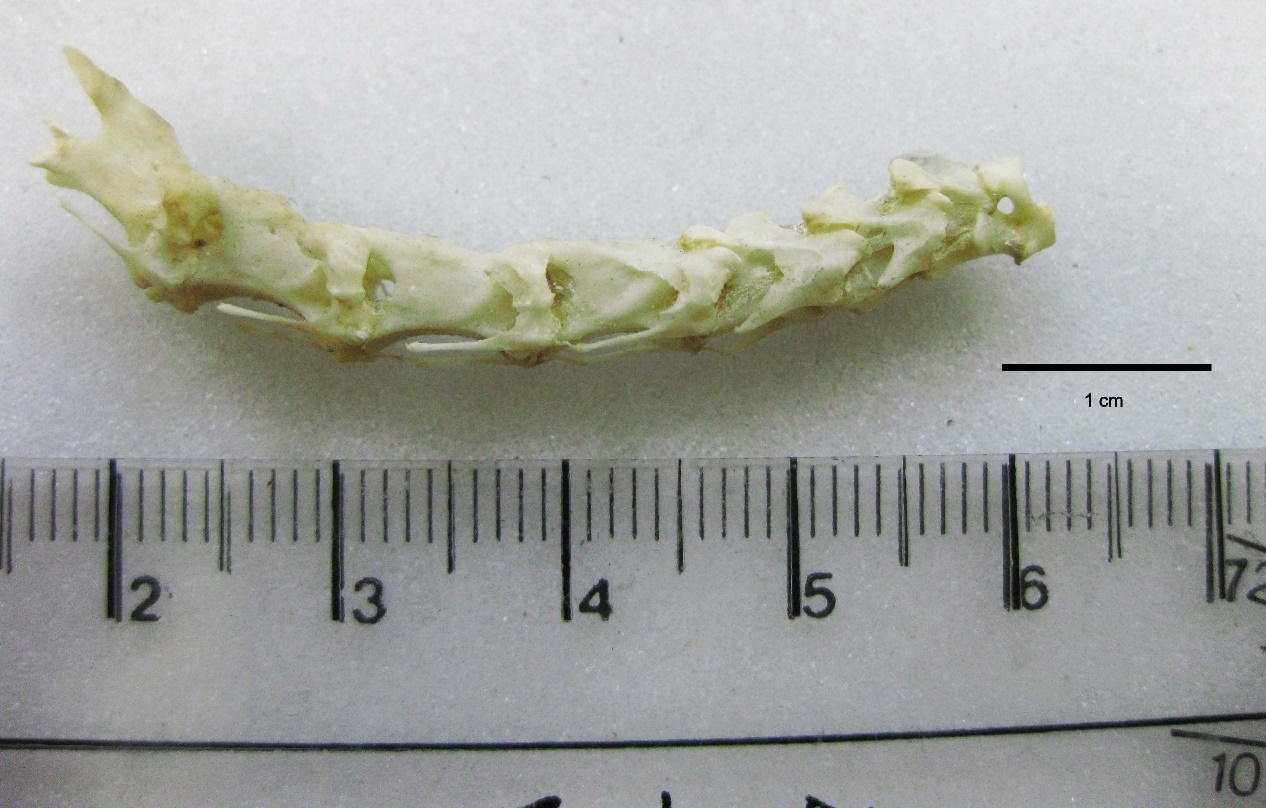
**

(b)


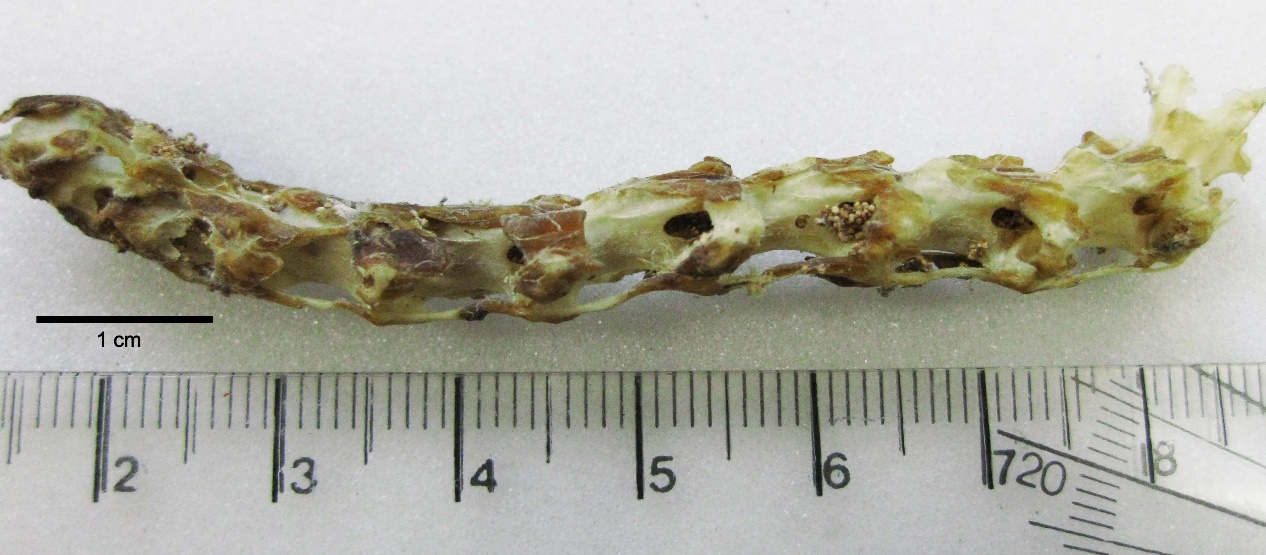


(c)

**
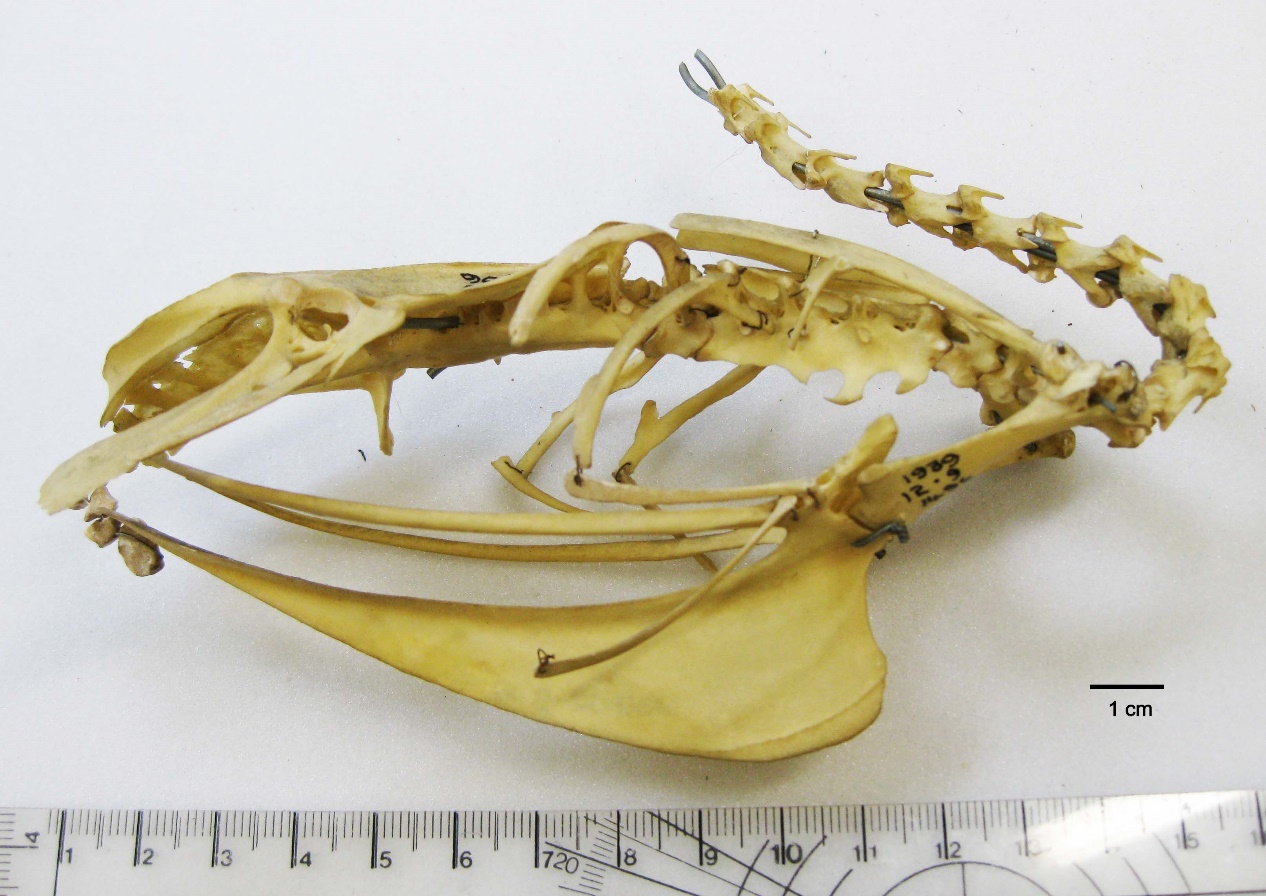
**

(d)


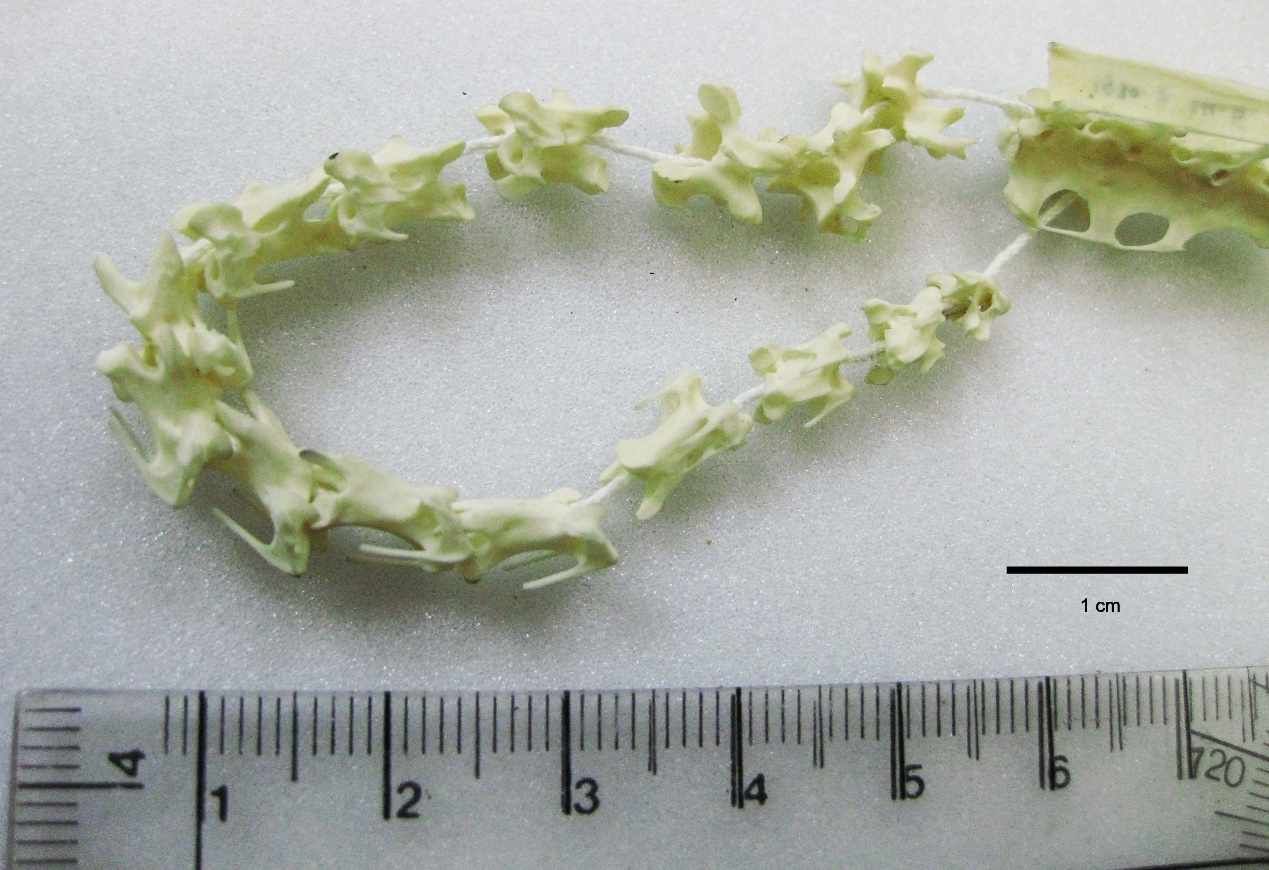


(e)


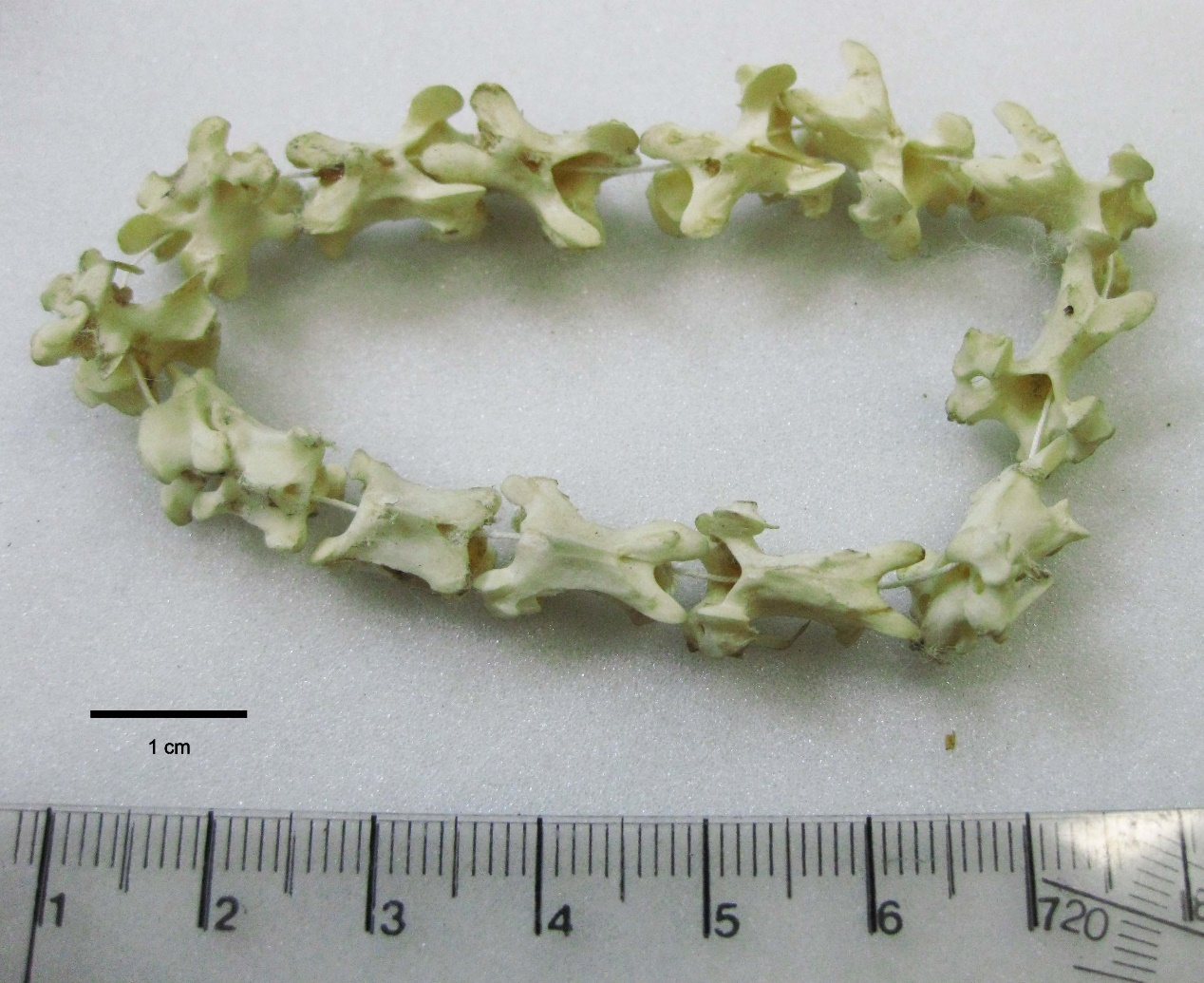

Supplement: S7 Fig — (a) Crypturellus obsoletus (NHMUK S/1972.1.23); (b) Crypturellus undulatus (NHMUK S/1972.2.6.7); (c) Eudromia elegans (NHMUK S/1972.1496); (d) Nothura maculosa (NHMUK S/1972.3.24.5); (e) Rhynchotus rufescens (NHMUK S/1972.2.16.62). (DOCX) [file pone.0143834.s007.docx]
