# Supplementary material for: Osteological and Soft-Tissue Evidence for Pneumatization in the Cervical Column of the Ostrich (Struthio camelus) and Observations on the Vertebral Columns of Non-Volant, Semi-Volant and Semi-Aquatic Birds
Source: PLoS One. 2015 Dec 9;10(12):e0143834. doi: 10.1371/journal.pone.0143834 (PMC4674062; doi:10.1371/journal.pone.0143834)

**Supporting Information**

**S8 Fig. Kiwis.** (a) *Apteryx australis haasti* (NHMUK 1456); (b) *Apteryx australis lawri* (NHMUK 1488); (c) *Apteryx oweni* (NHMUK 1458).

(a)


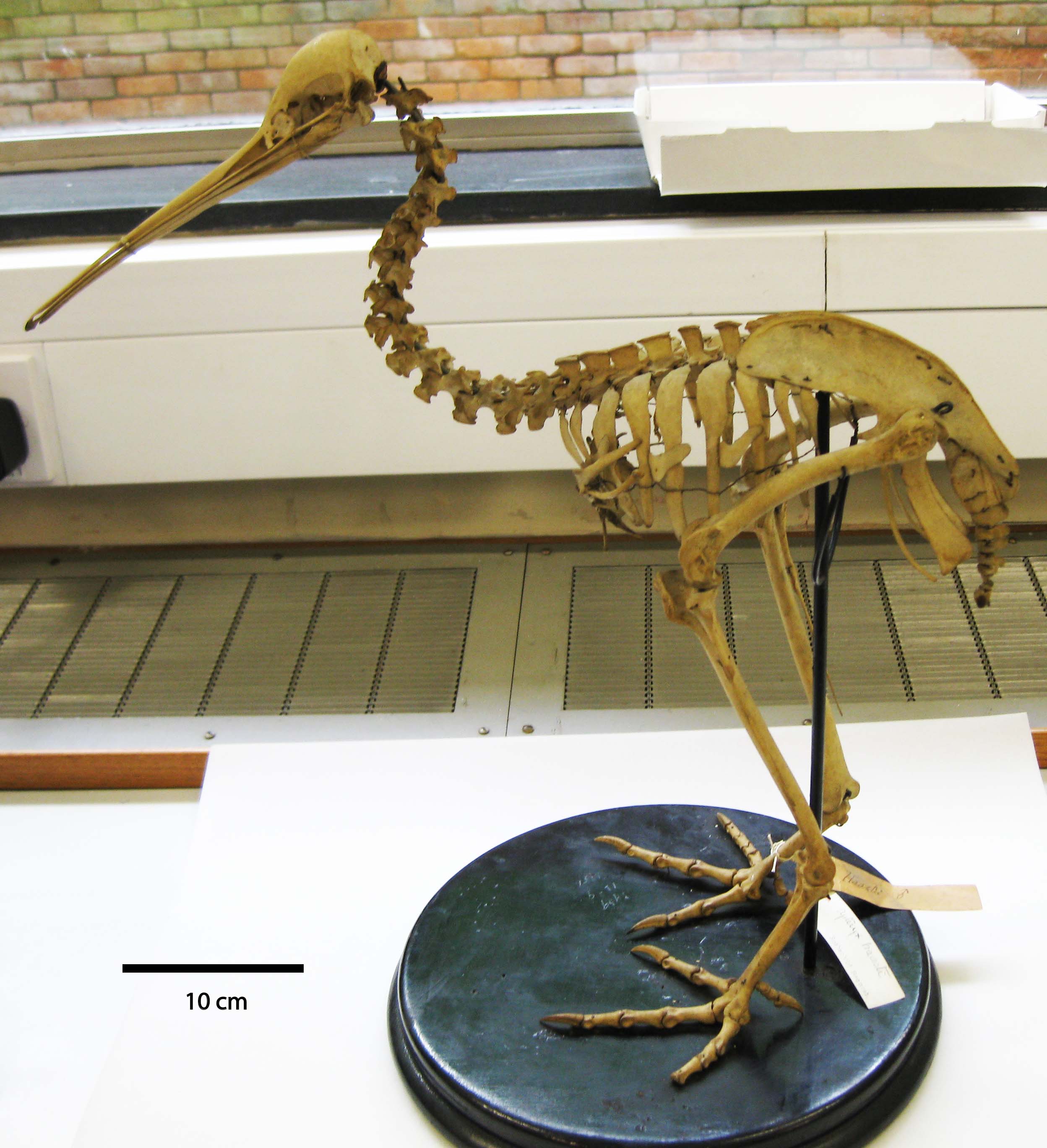


(b) + (c)


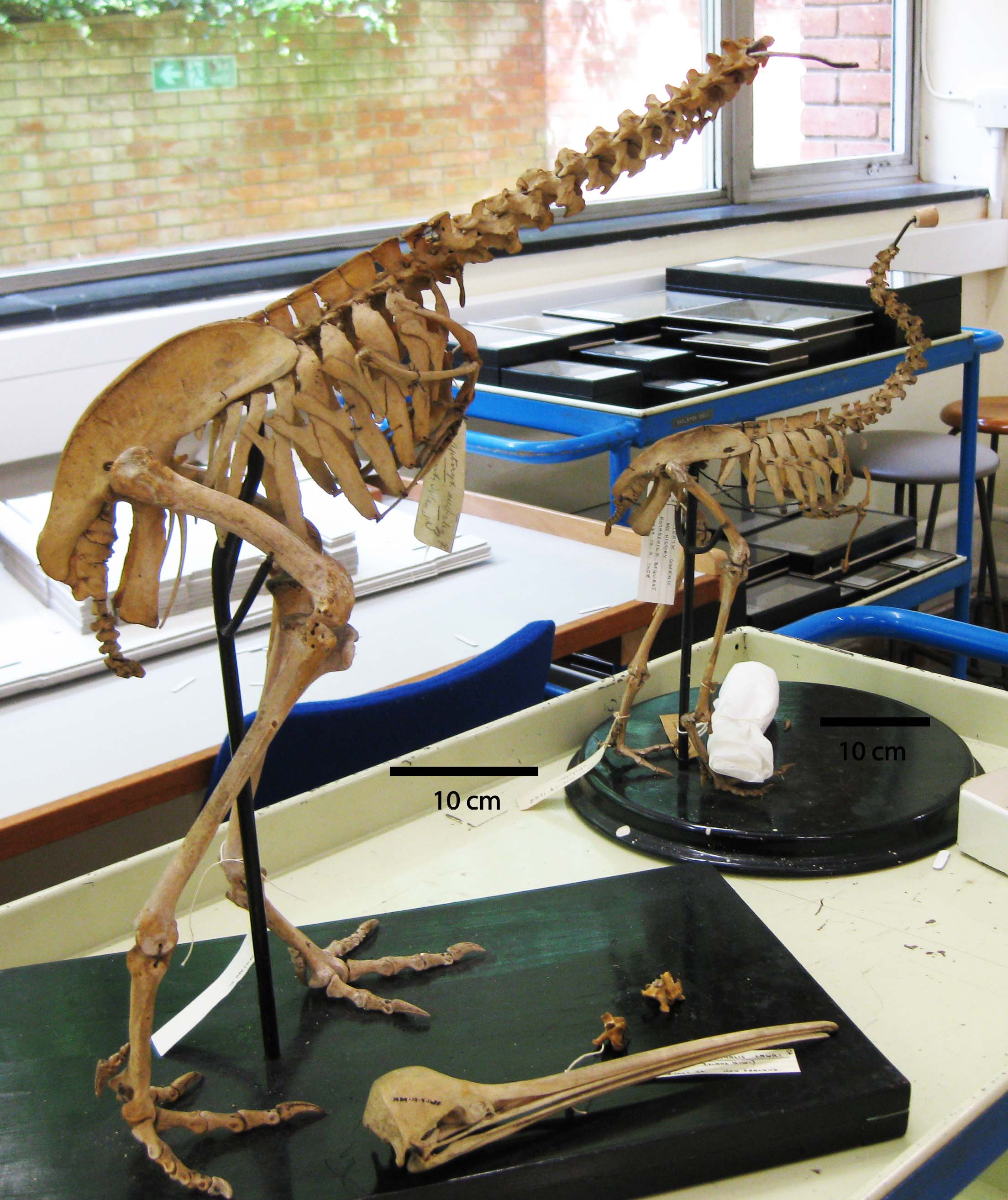

Supplement: S8 Fig — (a) Apteryx australis haasti (NHMUK 1456); (b) Apteryx australis lawri (NHMUK 1488); (c) Apteryx oweni (NHMUK 1458). (DOCX) [file pone.0143834.s008.docx]
