# Supplementary material for: Osteological and Soft-Tissue Evidence for Pneumatization in the Cervical Column of the Ostrich (Struthio camelus) and Observations on the Vertebral Columns of Non-Volant, Semi-Volant and Semi-Aquatic Birds
Source: PLoS One. 2015 Dec 9;10(12):e0143834. doi: 10.1371/journal.pone.0143834 (PMC4674062; doi:10.1371/journal.pone.0143834)

**Supporting Information**

**S9 Fig.** **Ducks.** (a) *Anas* sp. indet. (BRSUV unregistered); (b) *Melanitta* sp. indet. (BRSMG Af974).

(a)


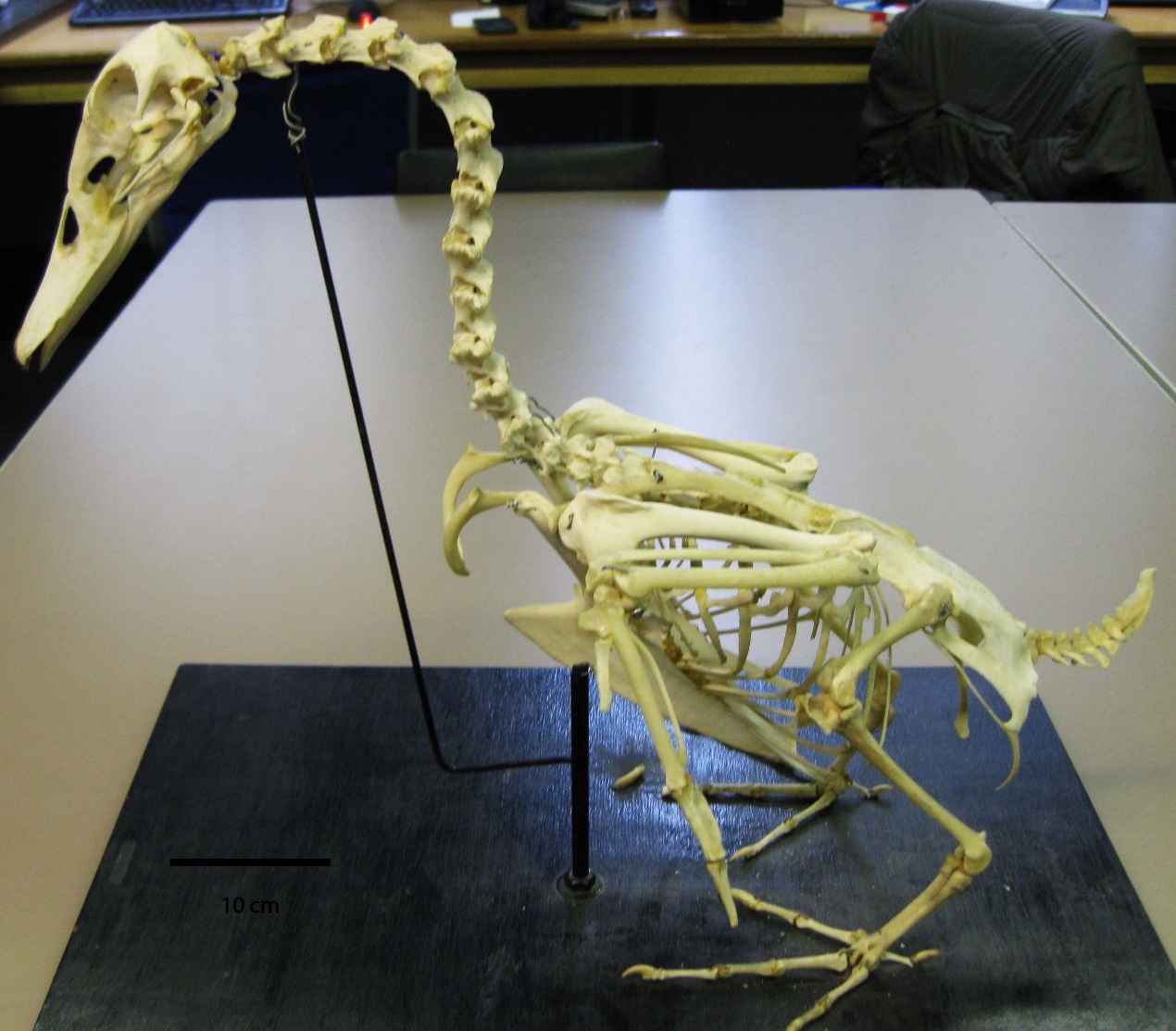


(b)


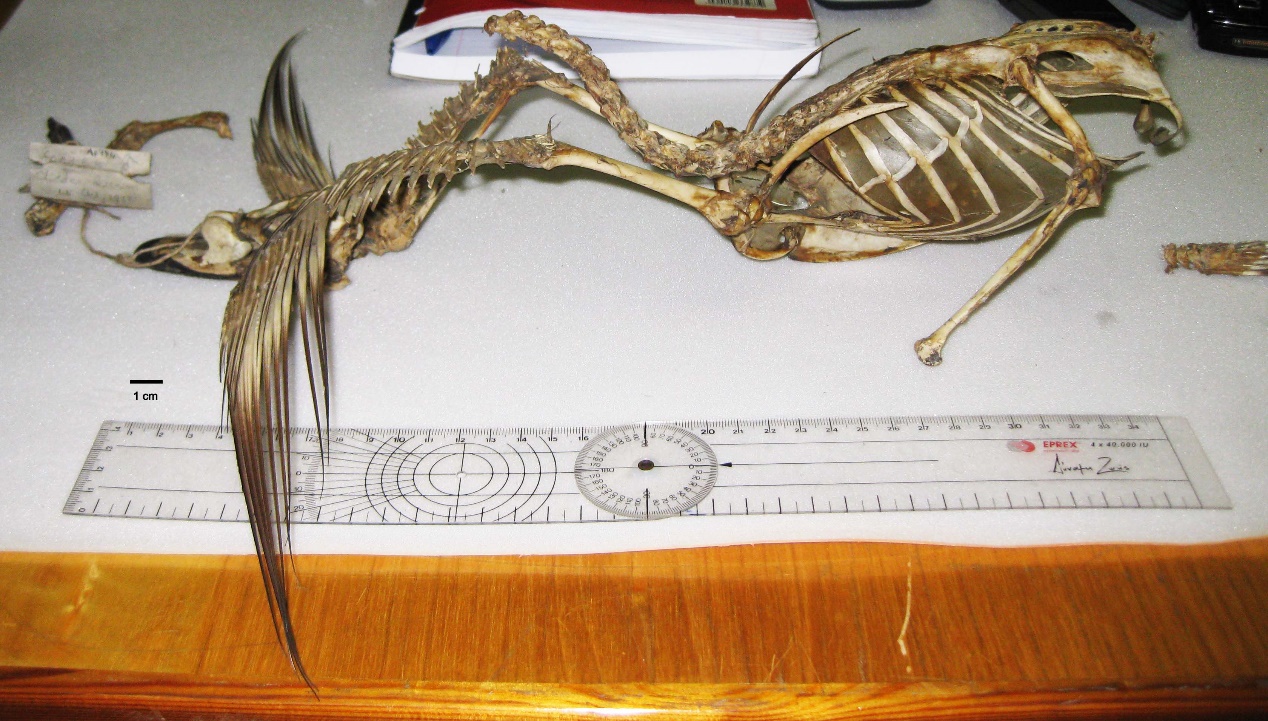

Supplement: S9 Fig — (a) Anas sp. indet. (BRSUV unregistered); (b) Melanitta sp. indet. (BRSMG Af974). (DOCX) [file pone.0143834.s009.docx]
