# Supplementary material for: Osteological and Soft-Tissue Evidence for Pneumatization in the Cervical Column of the Ostrich (Struthio camelus) and Observations on the Vertebral Columns of Non-Volant, Semi-Volant and Semi-Aquatic Birds
Source: PLoS One. 2015 Dec 9;10(12):e0143834. doi: 10.1371/journal.pone.0143834 (PMC4674062; doi:10.1371/journal.pone.0143834)
Supplement: S1 File — (DOCX) [file pone.0143834.s013.docx]

**Supporting Information**

**S1. Air sac system in ostrich**

In the ostrich, the cervical air sacs communicate freely with each other, forming an unpaired structure that extends up the neck as far as the head, and is closely related to the cervical and thoracic vertebrae and the vertebral canal [15]. The paired lateral clavicular air sacs lie ventrolaterally in the cranial thoracic region, with sternal and axillary diverticula [15,8]. The axillary diverticula do not enter the humeri. The left and right medial clavicular air sacs fuse with each other, forming a centrally situated air sac [15,8]. This air sac has diverticula between the large blood vessels above and cranially to the heart, as well as diverticula that extend caudally along the dorsal and ventral surfaces of the esophagus [15,8]. The diverticulum along the ventral surface of the esophagus communicates with a large gastric diverticulum [15,8]. The latter extends along the caudal surface of the proventriculus, curves ventrally around its distal border and into the gizzard. The paired cranial thoracic air sacs are small and lie ventrally to the lungs, against the ribs between the horizontal and oblique septa [15,8]. They are separated from the clavicular and caudal thoracic air sacs by transverse septa [15]. The paired caudal thoracic air sacs are larger than the cranial air sacs and occupy a similar region, but more caudally [15]. The right abdominal air sac is relatively small and lies to the right of the mesentery, dorsally to the liver. The left abdominal air sac is large and lies to the left of the mesentery [15]. Both abdominal air sacs have femoral and perirenal diverticula [15,8]. The femoral diverticulum enters the proximal end of the femur, invades the hip joint and forms an extensive intramuscular diverticulum [15]. The latter diverticulum extends distally along the medial aspect of the limb to the level of the stifle. The perirenal diverticulum extends over and around the kidneys and extends almost to the caudal limit of the abdomen [15]. In the ostrich Baumel et al. [63] recognize 56 vertebrae in three vertebral sections: cervical (19), thoracic (29), and caudal (8). Each vertebra consists of a body with cranial and caudal articular surfaces, an arch with cranial and caudal articular processes, and transverse processes. The development of such morphological landmark parts differ between the vertebrae. The cervical vertebrae increase in size from cranial to caudal. The first 17 cervical vertebrae have large transverse foramina at the bases of the transverse processes. The foramina of the atlas may be open ventrally [15]. The first five thoracic vertebrae are separate and carry vertebral ribs. The rest of the thoracic vertebrae fuse extensively with each other as well as with the pelvis, forming a large synsacrum [15]. For a visual example of the internal structure of the ostrich see **S1 Fig.**
