# Supplementary material for: Osteological and Soft-Tissue Evidence for Pneumatization in the Cervical Column of the Ostrich (Struthio camelus) and Observations on the Vertebral Columns of Non-Volant, Semi-Volant and Semi-Aquatic Birds
Source: PLoS One. 2015 Dec 9;10(12):e0143834. doi: 10.1371/journal.pone.0143834 (PMC4674062; doi:10.1371/journal.pone.0143834)
Supplement: S1 Table — PI%* is derived as the number of pneumatic elements (i.e. vertebral and appendicular elements with pneumatic foramina) over the number of total elements (after [4]). Wherever elements come in pairs in an animal, pneumatization stands present for the whole element even if it is present in one of the two elements in that pair. Abbreviations: HM, humerus; FM, femur; SC, scapula; CC, coracoid; ST, sternum; RB, ribs; FC, furcula; TT, tibiotarsus; TM, tarsometatarsus. (DOCX) [file pone.0143834.s014.docx]

**Supporting Information**

**S1 Table.** Pneumaticity states of appendicular elements of the 11 avian taxa based on the presence (+)/absence (-) of pneumatic foramina. PI%* is derived as the number of pneumatic elements (i.e. vertebral and appendicular elements with pneumatic foramina) over the number of total elements (after [4]). Wherever elements come in pairs in an animal, pneumatization stands present for the whole element even if it is present in one of the two elements in that pair. Abbreviations: HM, humerus; FM, femur; SC, scapula; CC, coracoid; ST, sternum; RB, ribs; FC, furcula; TT, tibiotarsus; TM, tarsometatarsus.

| **Taxa** | **specimens** | **HM** | **FM** | **SC** | **CC** | **ST** | **RB** | **FC** | **TT** | **TM** | **PI%* (vertebrae +appendicular)** |
| --- | --- | --- | --- | --- | --- | --- | --- | --- | --- | --- | --- |
| **Tinamiformes**  **Tinamou** | 1-NHMUK  2-NHMUK  3-NHMUK  4-NHMUK  5-NHMUK | +  +  +  +  + | +  +  +  +  + | +  +  +  +  + | -  -  +  +  + | -  -  +  +  + | +  -  +  +  + | +  +  +  +  + | +  +  +  +  + | +  +  +  +  + | 10/13=77%  9/13=69%  12/13=92%  12/13=92%  12/13=92% |
| **Apterygiformes**  **Kiwi** | 1-NHMUK  2-NHMUK  3-NHMUK | +  +  - | +  +  - | +  -  - | +  -  - | +  +  - | +  +  - | -  +  - | +  -  - | -  -  - | 10/13=77%  8/13=61%  3/13=23% |
| **Dinornithiformes**  **Moa** | 1-BRSMG | N/A | + | N/A | N/A | + | + | - | + | - | 7/10=70% |
| **Casuariiformes**  **Cassowary** | 1-BRSMG | + | + | - | + | + | + | - | - | - | 7/13=54% |
| **Dromaiformes**  **Emu** | 1-BRSMG | - | + | + | - | + | + | + | - | + | 10/13=77% |
| **Struthioniformes**  **Ostrich** | 1-BRSMG | + | + | + | + | + | + | + | - | - | 11/13=85% |
| **Rheiformes**  **Rhea** | 1-NHMUK | + | + | + | + | + | + | + | + | + | 13/13=100% |
| **Anseriformes**  **Duck** | 1-BRSUV  2-BRSMG | +  + | +  + | -  - | +  + | +  - | -  - | -  - | +  + | +  + | 10/13=77% |
| **Gaviiformes**  **Loon** | 1-NHMUK  2-NHMUK | +  + | +  + | -  - | +  + | -  - | -  - | -  - | +  + | -  - | 6/13=46%  6/13=46% |
| **Podicipediformes**  **Grebe** | 1-NHMUK | + | + | - | + | - | - | - | + | + | 8/13=61% |
| **Sphenisciformes**  **Penguin** | 1-BRSUV  2-NHMUK | +  + | +  + | -  - | -  + | -  - | -  + | -  + | +  + | -  - | 3/13=23%  9/13=69% |
