# Supplementary material for: Osteological and Soft-Tissue Evidence for Pneumatization in the Cervical Column of the Ostrich (Struthio camelus) and Observations on the Vertebral Columns of Non-Volant, Semi-Volant and Semi-Aquatic Birds
Source: PLoS One. 2015 Dec 9;10(12):e0143834. doi: 10.1371/journal.pone.0143834 (PMC4674062; doi:10.1371/journal.pone.0143834)
Supplement: S2 Table — The measurements are in centimeters (cm). For the mounted specimens, total height corresponds to the straight distance from top of the skull to the ground and total length is the straight distance from anterior (i.e. tip of the beak) to posterior (i.e. the end of the last caudal/pygostyle) of the animal. In loose specimens (i.e. disarticulated/not mounted) the measured length is an approximation. Ostrich* refers only to the BRSMG mounted specimen. (DOCX) [file pone.0143834.s015.docx]

**Supporting Information**

**S2 Table.** Size measurements (total height and total length) obtained from the 11 avian taxa. The measurements are in centimeters (cm). For the mounted specimens, total height corresponds to the straight distance from top of the skull to the ground and total length is the straight distance from anterior (i.e. tip of the beak) to posterior (i.e. the end of the last caudal/pygostyle) of the animal. In loose specimens (i.e. disarticulated/not mounted) the measured length is an approximation. Ostrich* refers only to the BRSMG mounted specimen.

| **Taxa** | **specimens** | **St.pr.con.** | **Ontogenetic stage** | **Total animal height**  **(cm)** | **Total animal length**  **(cm)** |
| --- | --- | --- | --- | --- | --- |
| **Tinamiformes**  **Tinamou** | 1  2  3  4  5 | Complete,  loose | Adults | **-**  **-**  **-**  **-**  **-** | **15**  **20**  **20**  **15**  **20** |
| **Apterygiformes**  **Kiwi** | 1  2  3 | Complete,  articulated | Adult  Adult  Subadult | **43**  **46**  **40** | **45**  **48**  **42** |
| **Dinornithiformes**  **Moa** | 1 | Complete,  articulated | Subadult | **136** | **89** |
| **Casuariiformes**  **Cassowary** | 1 | Complete  articulated, skull missing | Adult | **130** | **68** |
| **Dromaiformes**  **Emu** | 1 | Complete,  articulated | Subadult | **155** | **70** |
| **Struthioniformes**  **Ostrich*** | 1 | Complete,  articulated | Subadults | **220** | **130** |
| **Rheiformes**  **Rhea** | 1 | Complete,  loose | Adult | **-** | **100** |
| **Anseriformes**  **Duck** | 1  2 | Complete,  articulated | Subadults | **42**  **18** | **60**  **36** |
| **Gaviiformes**  **Loon** | 1  2 | Complete,  loose | Adults | **-**  **-** | **50**  **45** |
| **Podicipediformes**  **Grebe** | 1 | Complete | Adult | **-** | **30** |
| **Sphenisciformes**  **Penguin** | 1  2 | Complete,  articulated skeletons | Adults | **40**  **46** | **68**  **46** |
