# Supplementary material for: Osteological and Soft-Tissue Evidence for Pneumatization in the Cervical Column of the Ostrich (Struthio camelus) and Observations on the Vertebral Columns of Non-Volant, Semi-Volant and Semi-Aquatic Birds
Source: PLoS One. 2015 Dec 9;10(12):e0143834. doi: 10.1371/journal.pone.0143834 (PMC4674062; doi:10.1371/journal.pone.0143834)
Supplement: S6 Table — (DOCX) [file pone.0143834.s019.docx]

**Supporting Information**

**S6 Table.** Proportion of vertebrae exhibiting pneumatic features in cassowary (*Casuarius galeatus -* BRSMG Af963).

| **Cervical pneumatic features** | | | | | | |
| --- | --- | --- | --- | --- | --- | --- |
| **Pneumatic foramina** | **Laminae** | **Fossae** | **PF+L** | **F + L** | **PF+F** | **Septated**  **PF** |
| All vertebrae and on/proximal to their ribs (variably expressed) | All vertebrae | All vertebrae | All vertebrae (variably expressed) | All vertebrae (variably expressed) | From mid-anterior to posterior vertebrae | All vertebrae (variably expressed) |
| **Thoracic pneumatic features** | | | | | | |
| **Pneumatic foramina** | **Laminae** | **Fossae** | **PF+L** | **F + L** | **PF+F** | **Septated**  **PF** |
| All vertebrae and on/proximal to their ribs (variably expressed) | All vertebrae | Anterior and middle vertebrae | All vertebrae, especially the posterior (variably expressed) | Anterior and middle vertebrae | Middle to posterior vertebrae | All vertebrae especially the posterior(variably expressed) |
| **Synsacral pneumatic features** | | | | | | |
| **Pneumatic foramina** | **Laminae** | **Fossae** | **PF+L** | **F + L** | **PF+F** | **Septated**  **PF** |
| - | - | Anterior vertebrae | Anterior vertebrae | Anterior vertebrae | Anterior vertebrae | - |
| **Caudal pneumatic features** | | | | | | |
| **Pneumatic foramina** | **Laminae** | **Fossae** | **PF+L** | **F + L** | **PF+F** | **Septated**  **PF** |
| - | - | - | - | - | - | - |
