# Supplementary material for: Osteological and Soft-Tissue Evidence for Pneumatization in the Cervical Column of the Ostrich (Struthio camelus) and Observations on the Vertebral Columns of Non-Volant, Semi-Volant and Semi-Aquatic Birds
Source: PLoS One. 2015 Dec 9;10(12):e0143834. doi: 10.1371/journal.pone.0143834 (PMC4674062; doi:10.1371/journal.pone.0143834)
Supplement: S7 Table — (DOCX) [file pone.0143834.s020.docx]

**Supporting Information**

**S7 Table.** Proportion of vertebrae exhibiting pneumatic features in rhea (*Rhea americana -* NHMUK 2.5.1).

| **Cervical pneumatic features** | | | | | | |
| --- | --- | --- | --- | --- | --- | --- |
| **Pneumatic foramina** | **Laminae** | **Fossae** | **PF+L** | **F + L** | **PF+F** | **Septated**  **PF** |
| All vertebrae (variably expressed) | All vertebrae (variably expressed) | All vertebrae (variably expressed) | Middle and posterior vertebrae | Middle and posterior vertebrae | Middle and posterior vertebrae | Middle and posterior vertebrae |
| **Thoracic pneumatic features** | | | | | | |
| **Pneumatic foramina** | **Laminae** | **Fossae** | **PF+L** | **F + L** | **PF+F** | **Septated**  **PF** |
| Mostly anterior and posterior vertebrae | All vertebrae (variably expressed) | All vertebrae (variably expressed) | All vertebrae (variably expressed) | - | All vertebrae (variably expressed) | - |
| **Synsacral pneumatic features** | | | | | | |
| **Pneumatic foramina** | **Laminae** | **Fossae** | **PF+L** | **F + L** | **PF+F** | **Septated**  **PF** |
| - | Anterior and posterior vertebrae | - | Anterior and posterior vertebrae | Posterior vertebrae | Posterior vertebrae | Anterior and posterior vertebrae |
| **Caudal pneumatic features** | | | | | | |
| **Pneumatic foramina** | **Laminae** | **Fossae** | **PF+L** | **F + L** | **PF+F** | **Septated**  **PF** |
| All vertebrae | - | - | - | - | - | All vertebrae |
