# Supplementary material for: Osteological and Soft-Tissue Evidence for Pneumatization in the Cervical Column of the Ostrich (Struthio camelus) and Observations on the Vertebral Columns of Non-Volant, Semi-Volant and Semi-Aquatic Birds
Source: PLoS One. 2015 Dec 9;10(12):e0143834. doi: 10.1371/journal.pone.0143834 (PMC4674062; doi:10.1371/journal.pone.0143834)
Supplement: S9 Table — (DOCX) [file pone.0143834.s022.docx]

**Supporting Information**

**S9 Table.** Proportion of vertebrae exhibiting pneumatic features in tinamous (*Crypturellus obsoletus -* NHMUK S/1972.1.23*; Crypturellus undulatus -* NHMUK S/1972.2.6.7*; Eudromia elegans -* NHMUK S/1972.1496*; Nothura maculosa -* NHMUK S/1972.3.24.5*; Rhynchotus rufescens -* NHMUK S/1972.2.16.62).

| **Cervical pneumatic features** | | | | | | |
| --- | --- | --- | --- | --- | --- | --- |
| **Pneumatic foramina** | **Laminae** | **Fossae** | **PF+L** | **F + L** | **PF+F** | **Septated**  **PF** |
| Middle to posterior vertebrae (variably expressed) | All vertebrae (variably expressed) | Middle to posterior vertebrae (variably expressed) | - | - | Anterior and middle vertebrae | - |
| **Thoracic pneumatic features** | | | | | | |
| **Pneumatic foramina** | **Laminae** | **Fossae** | **PF+L** | **F + L** | **PF+F** | **Septated**  **PF** |
| All vertebrae (variably expressed) | All vertebrae (variably expressed) | Anterior and middle vertebrae (rarely expressed) | All vertebrae (variably expressed) | - | - | Middle and posterior vertebrae (variably expressed) |
| **Synsacral pneumatic features** | | | | | | |
| **Pneumatic foramina** | **Laminae** | **Fossae** | **PF+L** | **F + L** | **PF+F** | **Septated**  **PF** |
| All vertebrae (variably expressed) | - | - | - | - | - | - |
| **Caudal pneumatic features** | | | | | | |
| **Pneumatic foramina** | **Laminae** | **Fossae** | **PF+L** | **F + L** | **PF+F** | **Septated**  **PF** |
| - | - | - | - | - | - | - |
