# Supplementary material for: Osteological and Soft-Tissue Evidence for Pneumatization in the Cervical Column of the Ostrich (Struthio camelus) and Observations on the Vertebral Columns of Non-Volant, Semi-Volant and Semi-Aquatic Birds
Source: PLoS One. 2015 Dec 9;10(12):e0143834. doi: 10.1371/journal.pone.0143834 (PMC4674062; doi:10.1371/journal.pone.0143834)
Supplement: S14 Table — (DOCX) [file pone.0143834.s027.docx]

**Supporting Information**

**S14 Table.** List of lamina abbreviations used in the main text (following [55], [56])

| **Lamina** | **Abbreviation** |
| --- | --- |
| Spinoprezygapophyseal | sprl |
| Spinopostzygapophyseal | spol |
| Centropostzygapophyseal | cpol |
| Centrohypapophyseal | chpl |
| Centrodiapophyseal | cdpl |
| Prezygodiapophyseal | prdl |
| Prezygoparapophyseal | prpl |
| Postzygodiapophyseal | podl |
